# Supplementary material for: Diversity and Ecology of Caudoviricetes Phages with Genome Terminal Repeats in Fecal Metagenomes from Four Dutch Cohorts
Source: Viruses. 2022 Oct 20;14(10):2305. doi: 10.3390/v14102305 (PMC9610469; doi:10.3390/v14102305)
Supplement: Supplementary file 1 [file viruses-14-02305-s001.zip › Figure_S1.pdf]

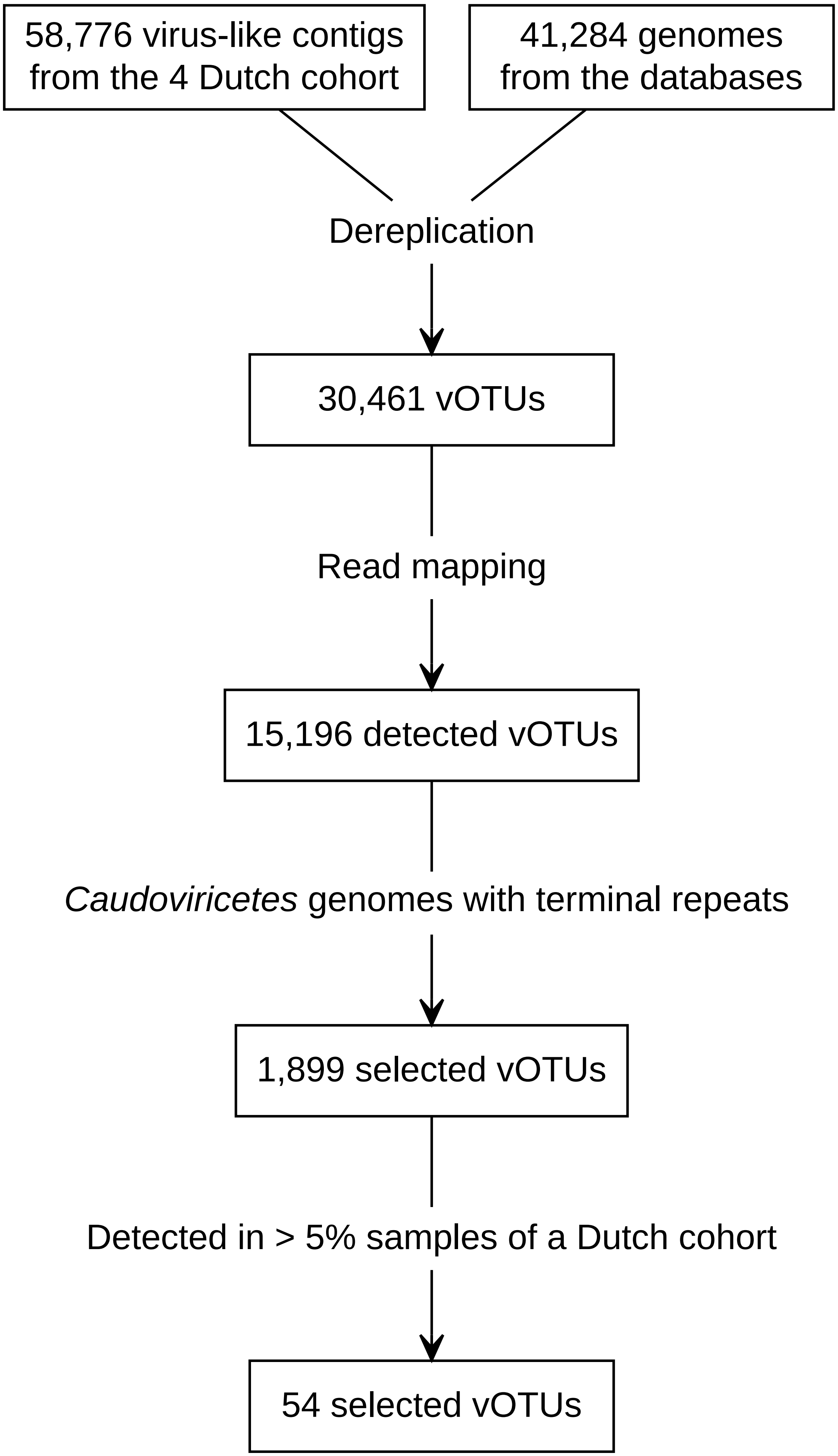
58,776 virus-like contigs from the 4 Dutch cohort
41,284 genomes from the databases
Dereplication
30,461 vOTUs
Read mapping
15,196 detected vOTUs
Caudoviricetes genomes with terminal repeats
1,899 selected vOTUs
Detected in > 5% samples of a Dutch cohort
54 selected vOTUs
