# Supplementary material for: Diversity and Ecology of Caudoviricetes Phages with Genome Terminal Repeats in Fecal Metagenomes from Four Dutch Cohorts
Source: Viruses. 2022 Oct 20;14(10):2305. doi: 10.3390/v14102305 (PMC9610469; doi:10.3390/v14102305)
Supplement: Supplementary file 1 [file viruses-14-02305-s001.zip › Material_S1.pdf]

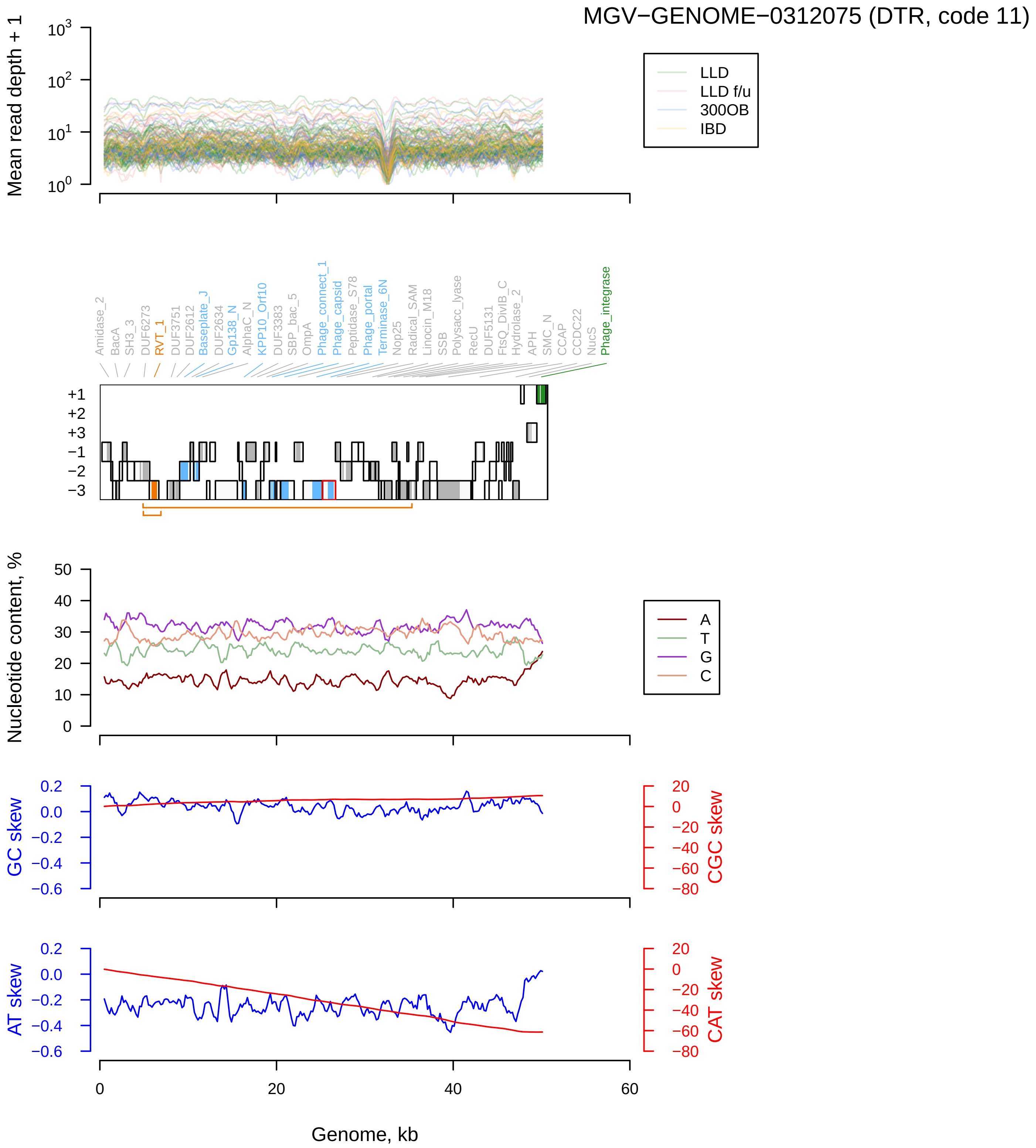

MGV−GENOME−0312075 (DTR, code 11)
Mean read depth + 1
LLD
LLD f/u
300OB
IBD
Amidase_2
BacA
SH3_3
DUF6273
RVT_1
DUF3751
DUF2612
Baseplate_J
DUF2634
Gp138_N
AlphaC_N
KPP10_Orf10
DUF3383
SBP_bac_5
OmpA
Phage_connect_1
Phage_capsid
Peptidase_S78
Phage_portal
Terminase_6N
Nop25
Radical_SAM
Linocin_M18
SSB
Polysacc_lyase
RecU
DUF5131
FtsQ_DivIB_C
Hydrolase_2
APH
SMC_N
CCAP
CCDC22
NucS
Phage_integrase
Nucleotide content, %
A
T
G
C
GC skew
CGC skew
AT skew
CAT skew
Genome, kb

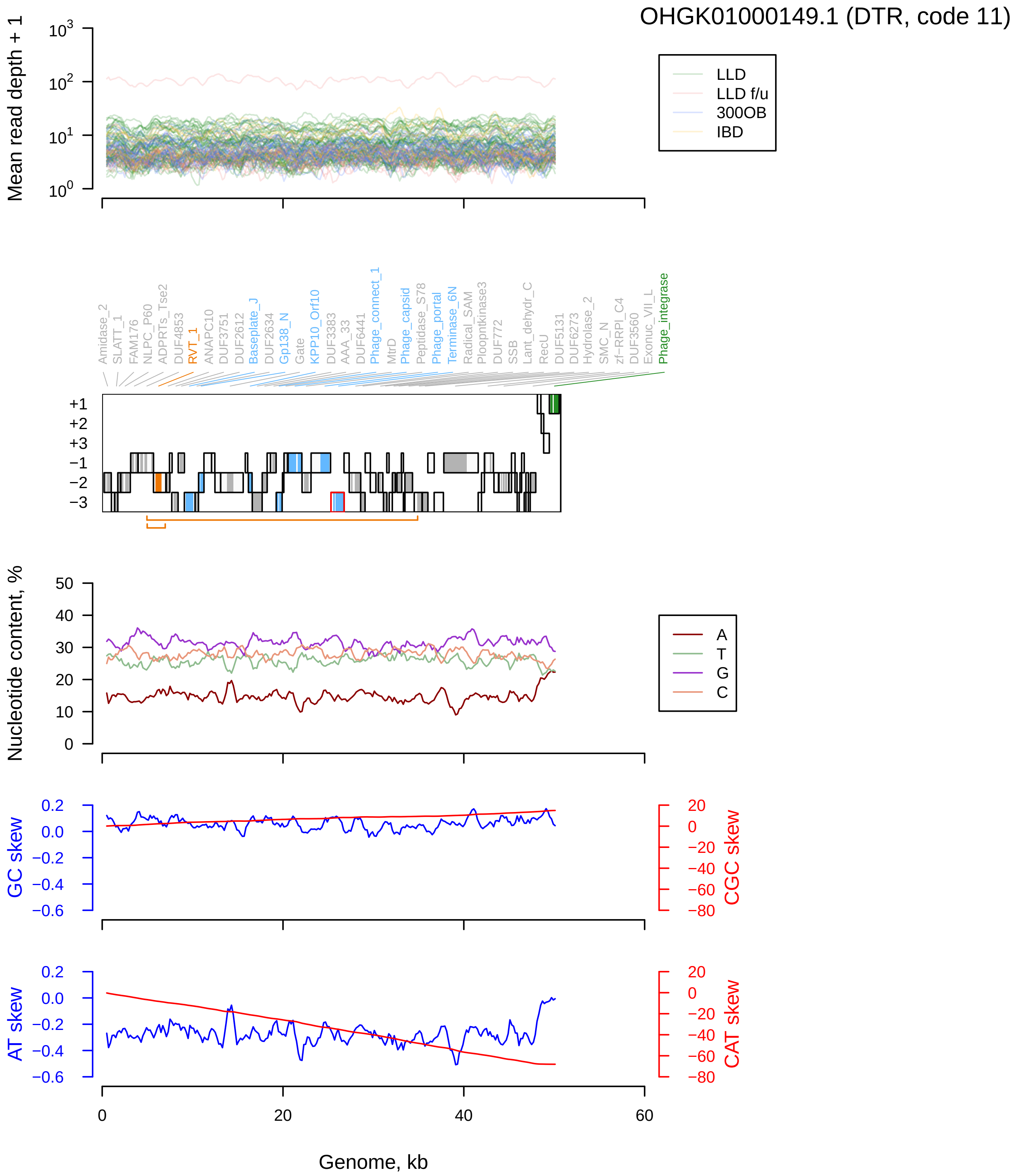
OHGK01000149.1 (DTR, code 11)
Mean read depth + 1
LLD
LLD f/u
300OB
IBD
Amidase_2
SLATT_1
FAM176
NLPC_P60
ADPRTs_Tse2
DUF4853
RVT_1
ANAPC10
DUF3751
DUF2612
Baseplate_J
DUF2634
Gp138_N
Gate
KPP10_Orf10
DUF3383
AAA_33
DUF6441
Phage_connect_1
MtrD
Phage_capsid
Peptidase_S78
Phage_portal
Terminase_6N
Radical_SAM
Ploopntkinase3
DUF772
SSB
Lant_dehydr_C
RecU
DUF5131
DUF6273
Hydrolase_2
SMC_N
zf-RRPI_C4
DUF3560
Exonuc_VII_L
Phage_integrase
Nucleotide content, %
A
T
G
C
GC skew
CGC skew
AT skew
CAT skew
Genome, kb

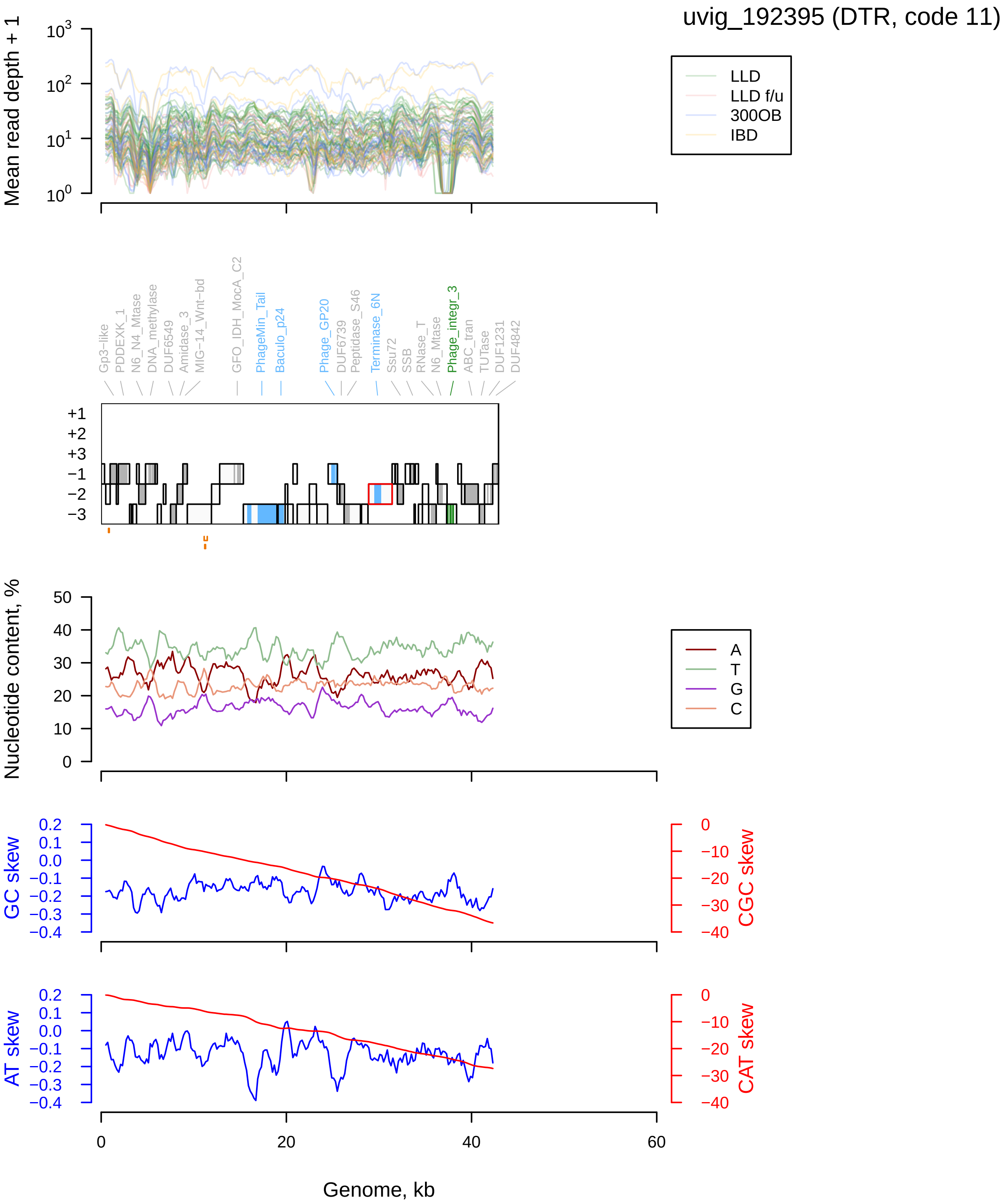
uvig_192395 (DTR, code 11)
Mean read depth + 1
LLD
LLD f/u
300OB
IBD
Gp3−like
PDDEXK_1
N6_N4_Mtase
DNA_methylase
DUF6549
Amidase_3
MIG−14_Wnt−bd
GFO_IDH_MocA_C2
PhageMin_Tail
Baculo_p24
Phage_GP20
DUF6739
Peptidase_S46
Terminase_6N
Ssu72
SSB
RNase_T
N6_Mtase
Phage_integr_3
ABC_tran
TUTase
DUF1231
DUF4842
+1
+2
+3
−1
−2
−3
Nucleotide content, %
A
T
G
C
GC skew
CGC skew
AT skew
CAT skew
0
20
40
60
Genome, kb

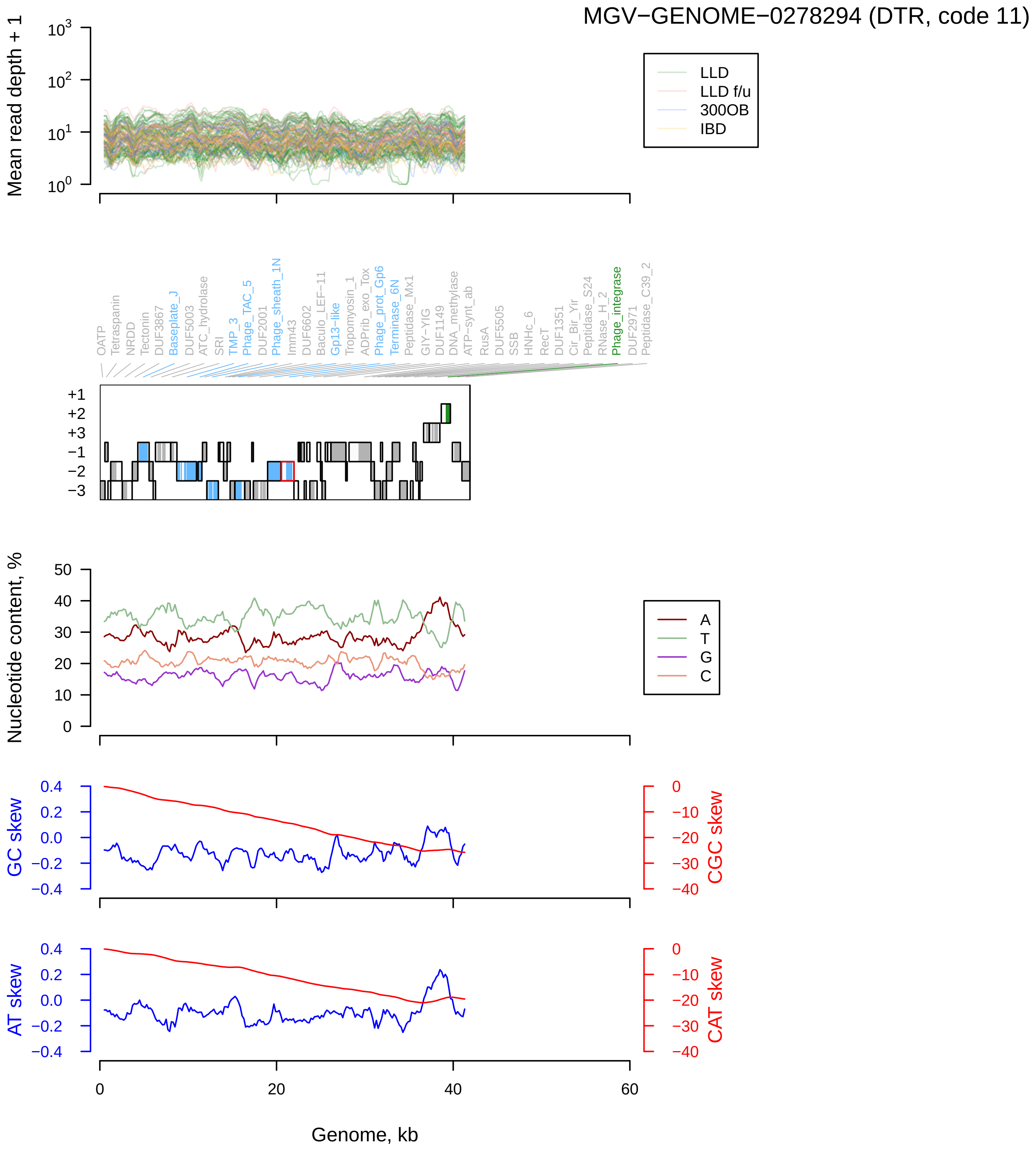
MGV−GENOME−0278294 (DTR, code 11)
Mean read depth + 1
LLD
LLD f/u
300OB
IBD
OATP
Tetraspanin
NRDD
Tectonin
DUF3867
Baseplate_J
DUF5003
ATC_hydrolase
SRI
TMP_3
Phage_TAC_5
DUF2001
Phage_sheath_1N
Imm43
DUF6602
Baculo_LEF−11
Gp13−like
Tropomyosin_1
ADPrib_exo_Tox
Phage_prot_Gp6
Terminase_6N
Peptidase_Mx1
GIY−YIG
DUF1149
DNA_methylase
ATP−synt_ab
RusA
DUF5505
SSB
HNHc_6
RecT
DUF1351
Cir_Bir_Yir
Peptidase_S24
RNase_H_2
Phage_integrase
DUF2971
Peptidase_C39_2
Nucleotide content, %
A
T
G
C
GC skew
CGC skew
AT skew
CAT skew
Genome, kb

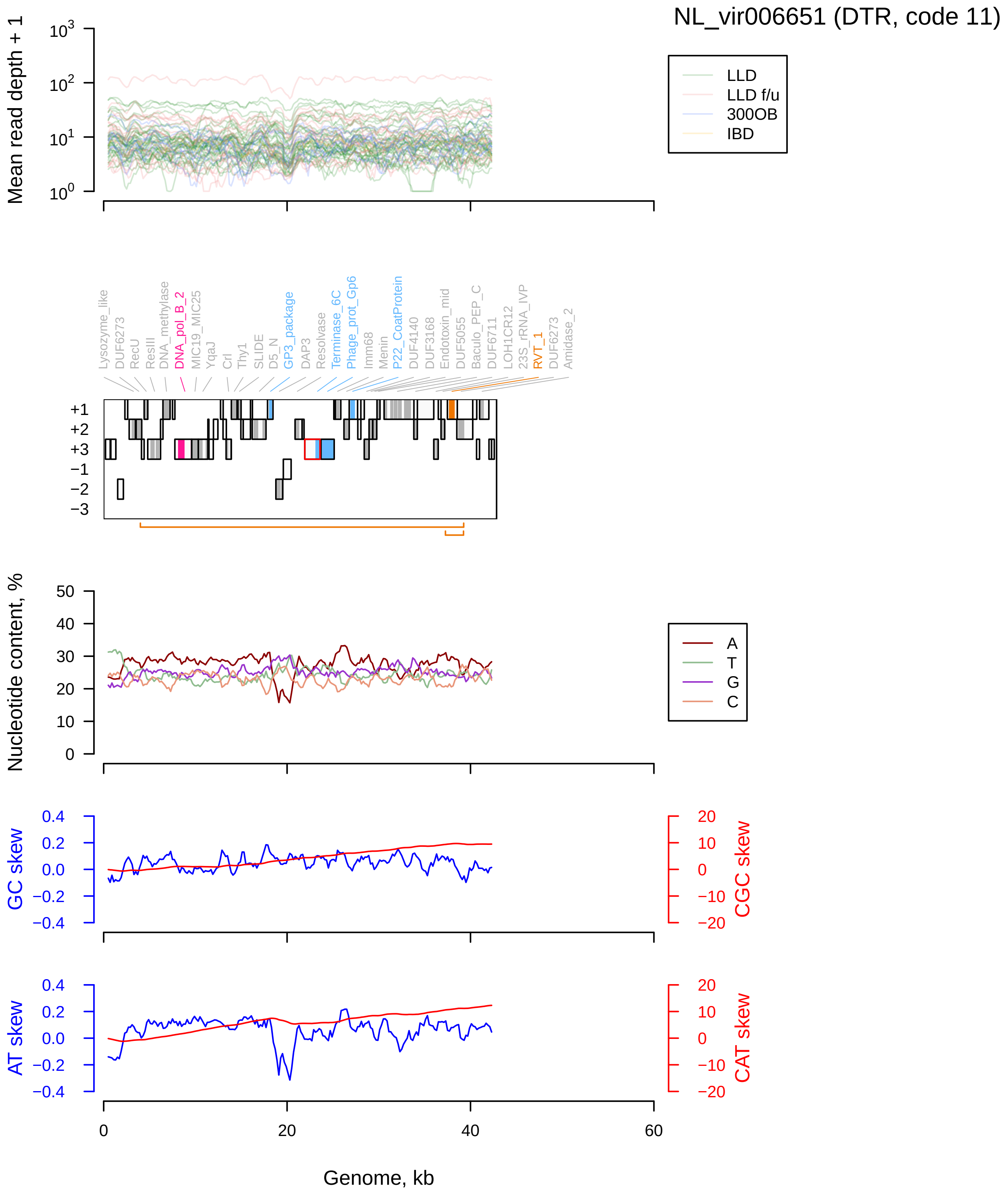

NL_vir006651 (DTR, code 11)
Mean read depth + 1
LLD
LLD f/u
300OB
IBD
Lysozyme_like
DUF6273
RecU
ResIII
DNA_methylase
DNA_pol_B_2
MIC19_MIC25
YqaJ
Crl
Thy1
SLIDE
D5_N
GP3_package
DAP3
Resolvase
Terminase_6C
Phage_prot_Gp6
Imm68
Menin
P22_CoatProtein
DUF4140
DUF3168
Endotoxin_mid
DUF5055
Baculo_PEP_C
DUF6711
LOH1CR12
23S_rRNA_IVP
RVT_1
DUF6273
Amidase_2
+1
+2
+3
−1
−2
−3
Nucleotide content, %
A
T
G
C
GC skew
CGC skew
AT skew
CAT skew
Genome, kb

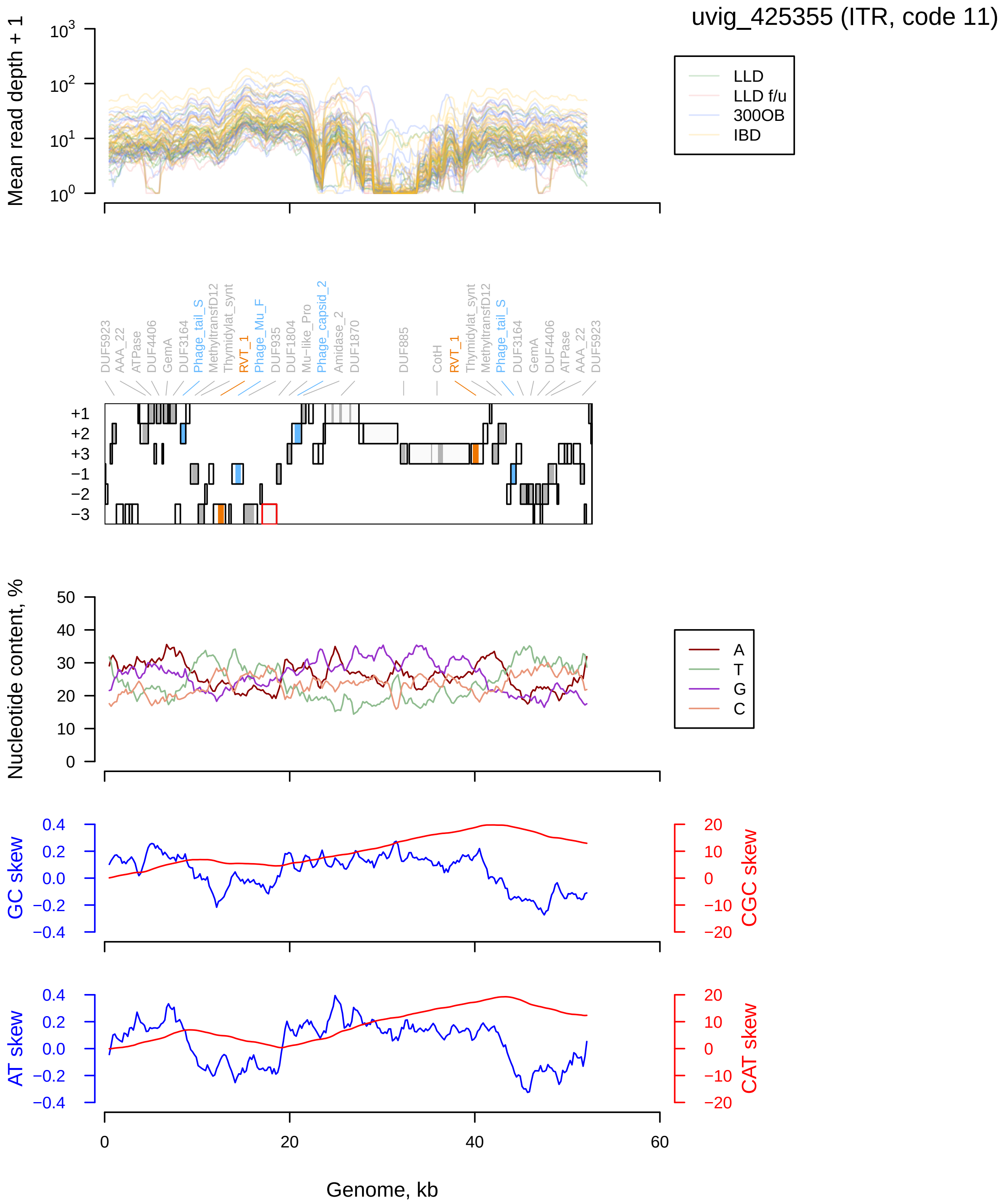
uvig_425355 (ITR, code 11)
Mean read depth + 1
LLD
LLD f/u
300OB
IBD
DUF5923
AAA_22
ATPase
DUF4406
GemA
DUF3164
Phage_tail_S
MethyltransfD12
Thymidylat_synt
RVT_1
Phage_Mu_F
DUF935
DUF1804
Mu-like_Pro
Phage_capsid_2
Amidase_2
DUF1870
DUF885
CotH
Nucleotide content, %
A
T
G
C
GC skew
CGC skew
AT skew
CAT skew
Genome, kb

MGV−GENOME−0272770 (ITR, code 15)

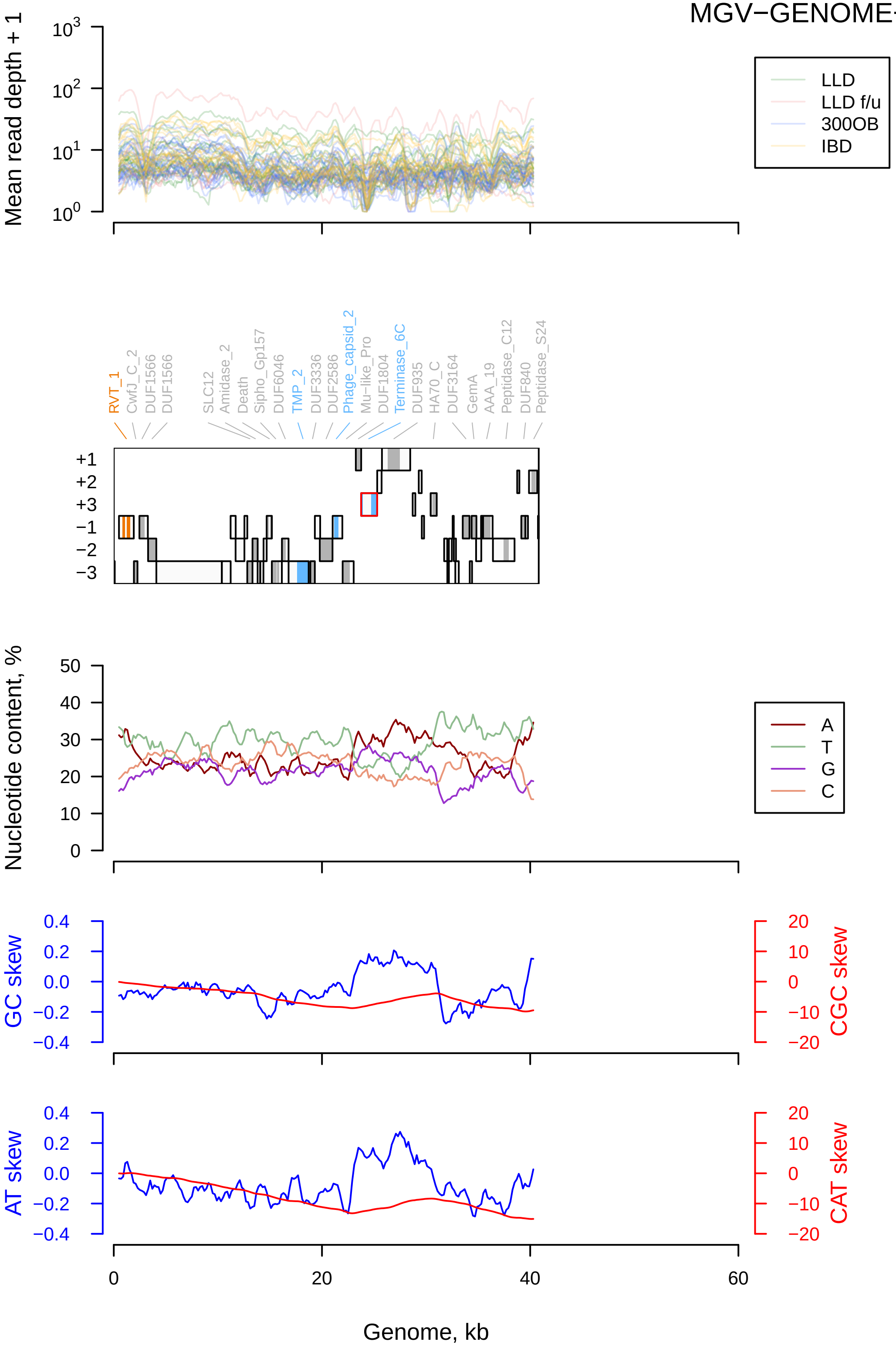

OLSK01002610.1 (DTR, code 11)

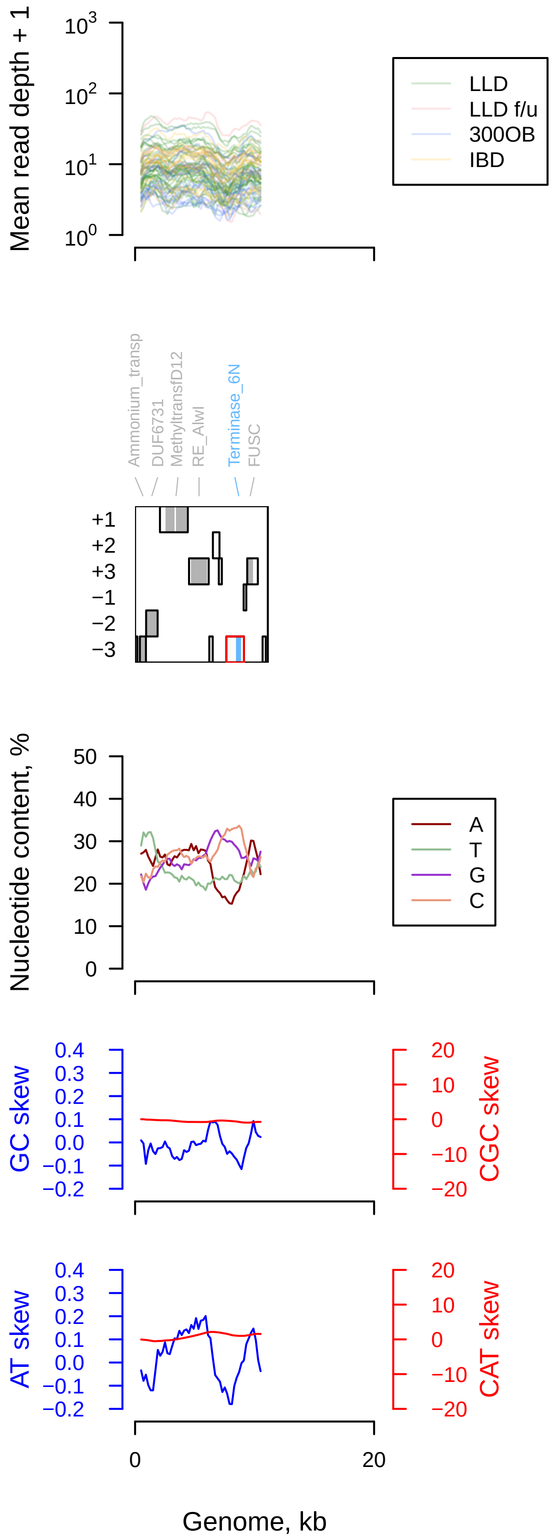

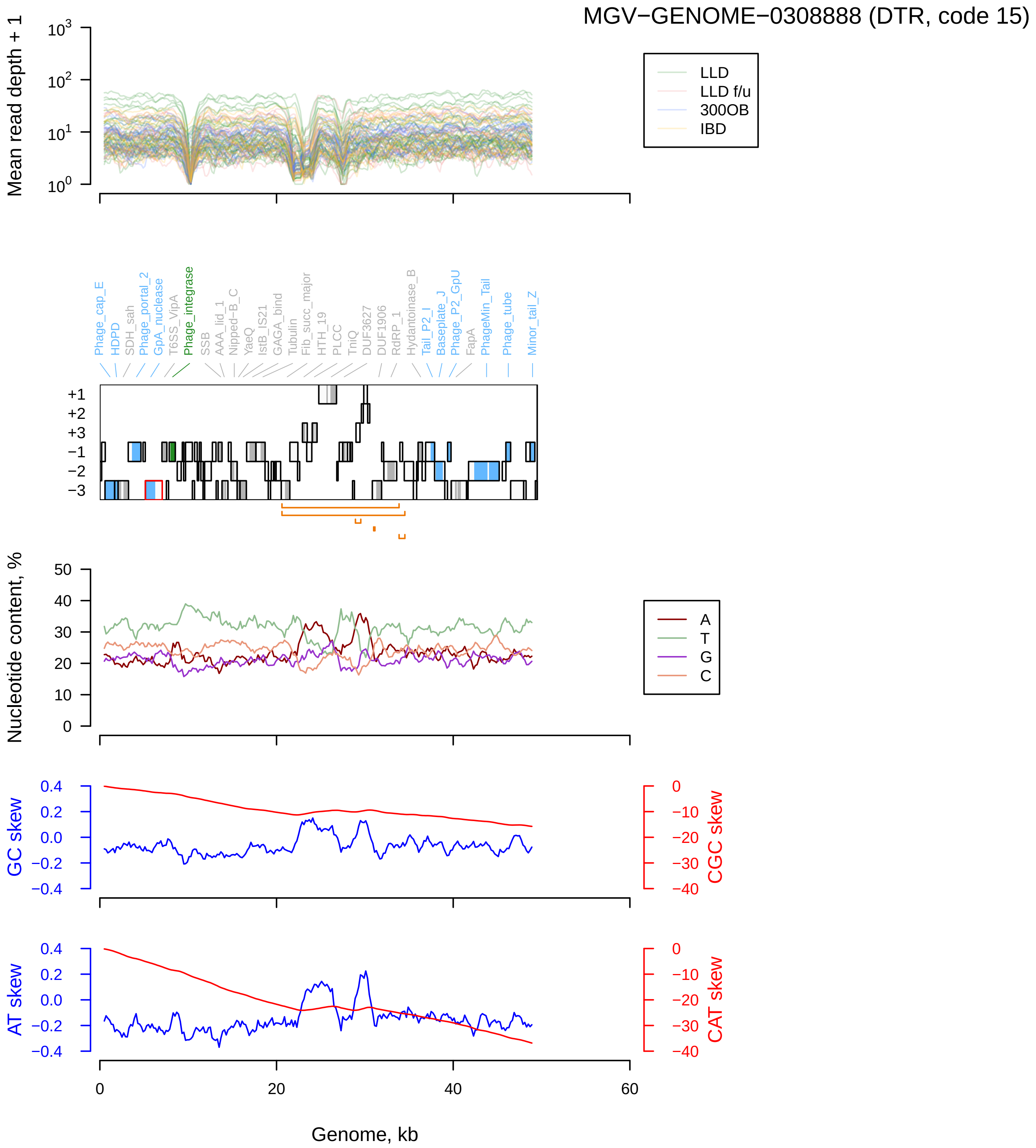

MGV−GENOME−0308888 (DTR, code 15)
Mean read depth + 1
LLD
LLD f/u
300OB
IBD
Phage_cap_E
HDPD
SDH_sah
Phage_portal_2
GpA_nuclease
T6SS_VipA
Phage_integrase
SSB
AAA_lid_1
Nipped−B_C
YaeQ
IstB_IS21
GAGA_bind
Tubulin
Fib_succ_major
HTH_19
PLCC
TniQ
DUF3627
DUF1906
RdRP_1
Hydantoinase_B
Tail_P2_I
Baseplate_J
Phage_P2_GpU
FapA
PhageMin_Tail
Phage_tube
Minor_tail_Z
Nucleotide content, %
A
T
G
C
GC skew
CGC skew
AT skew
CAT skew
Genome, kb

MGV−GENOME−0297536 (DTR, code 11)

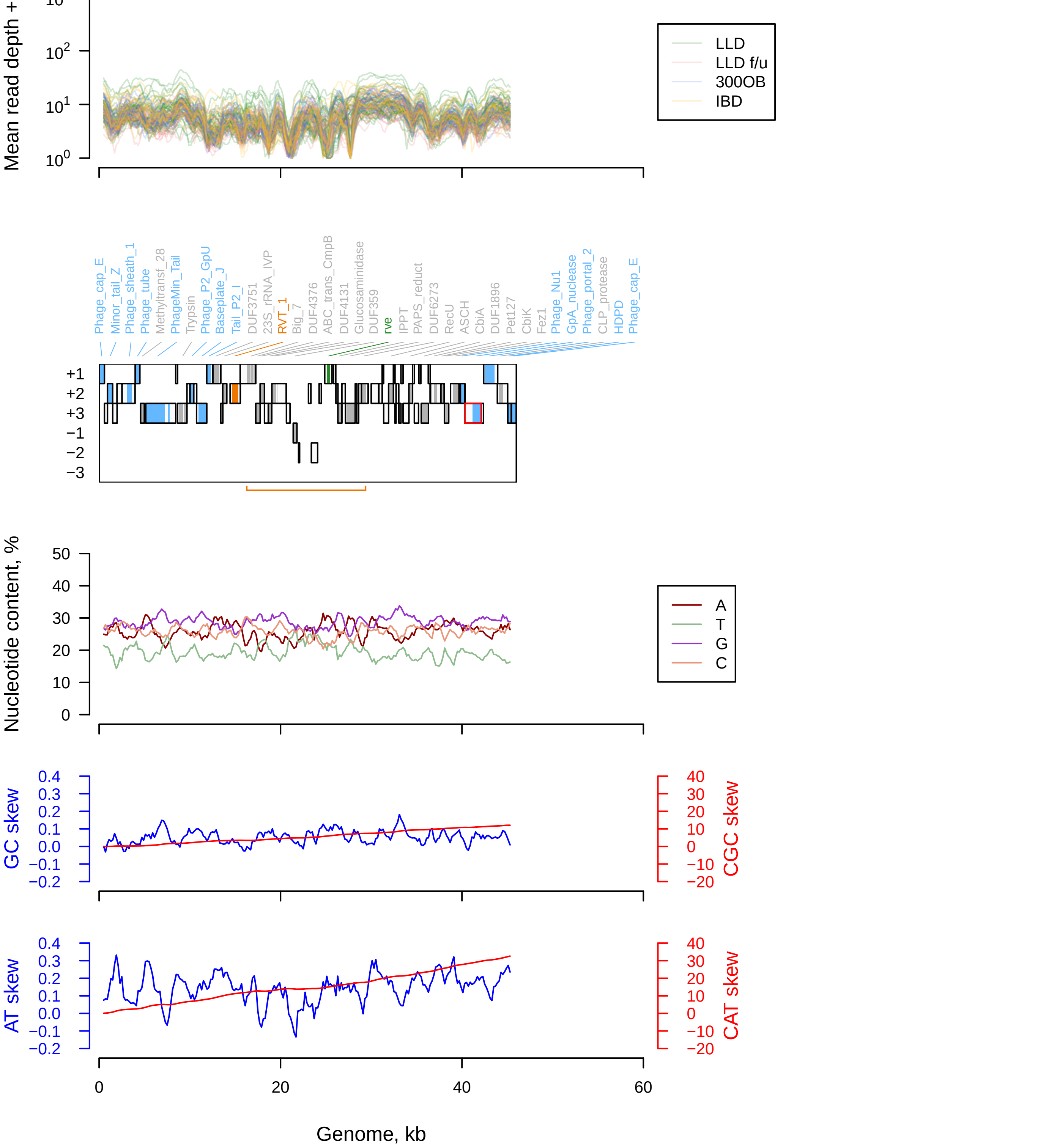

MGV−GENOME−0310879 (DTR, code 11)

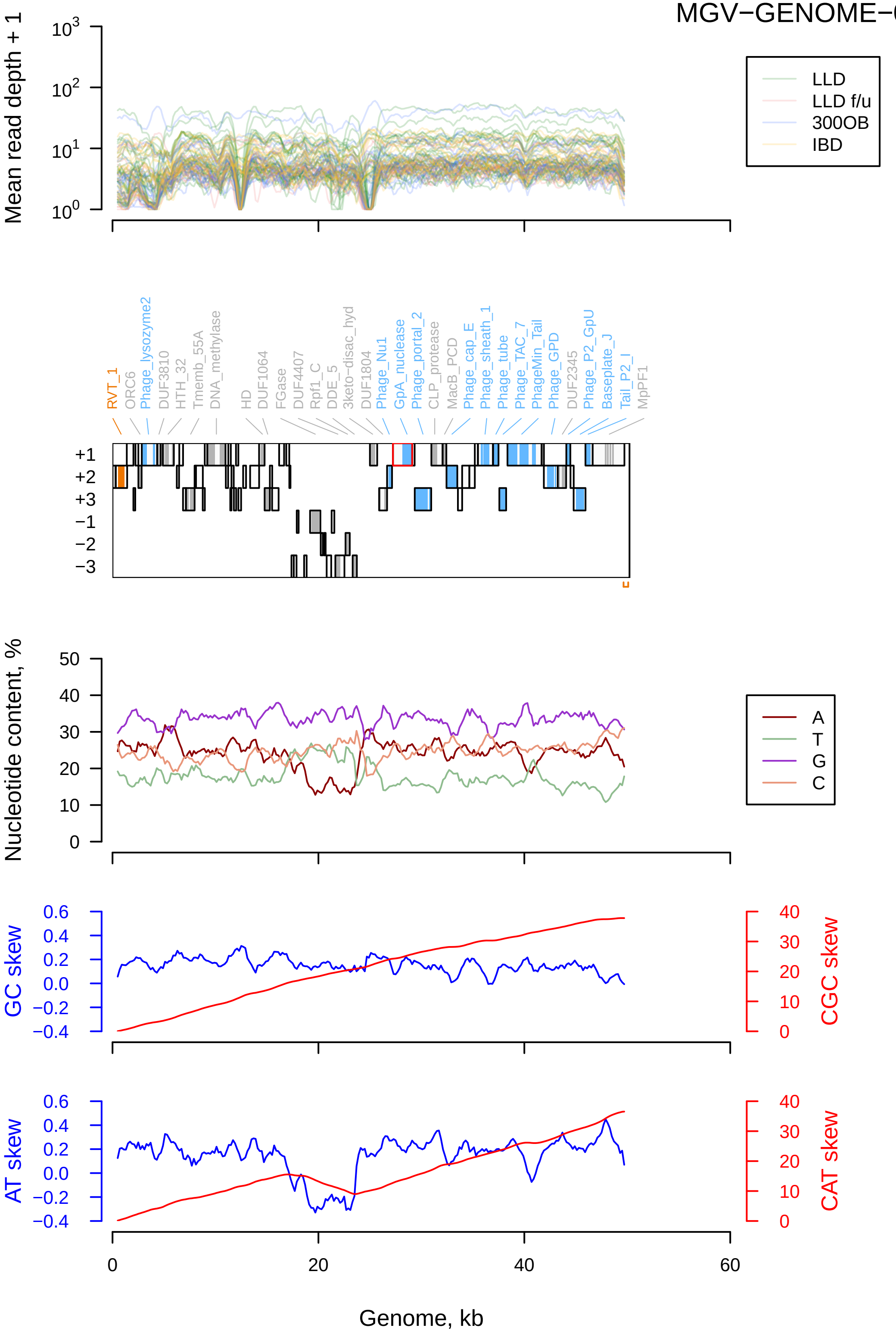

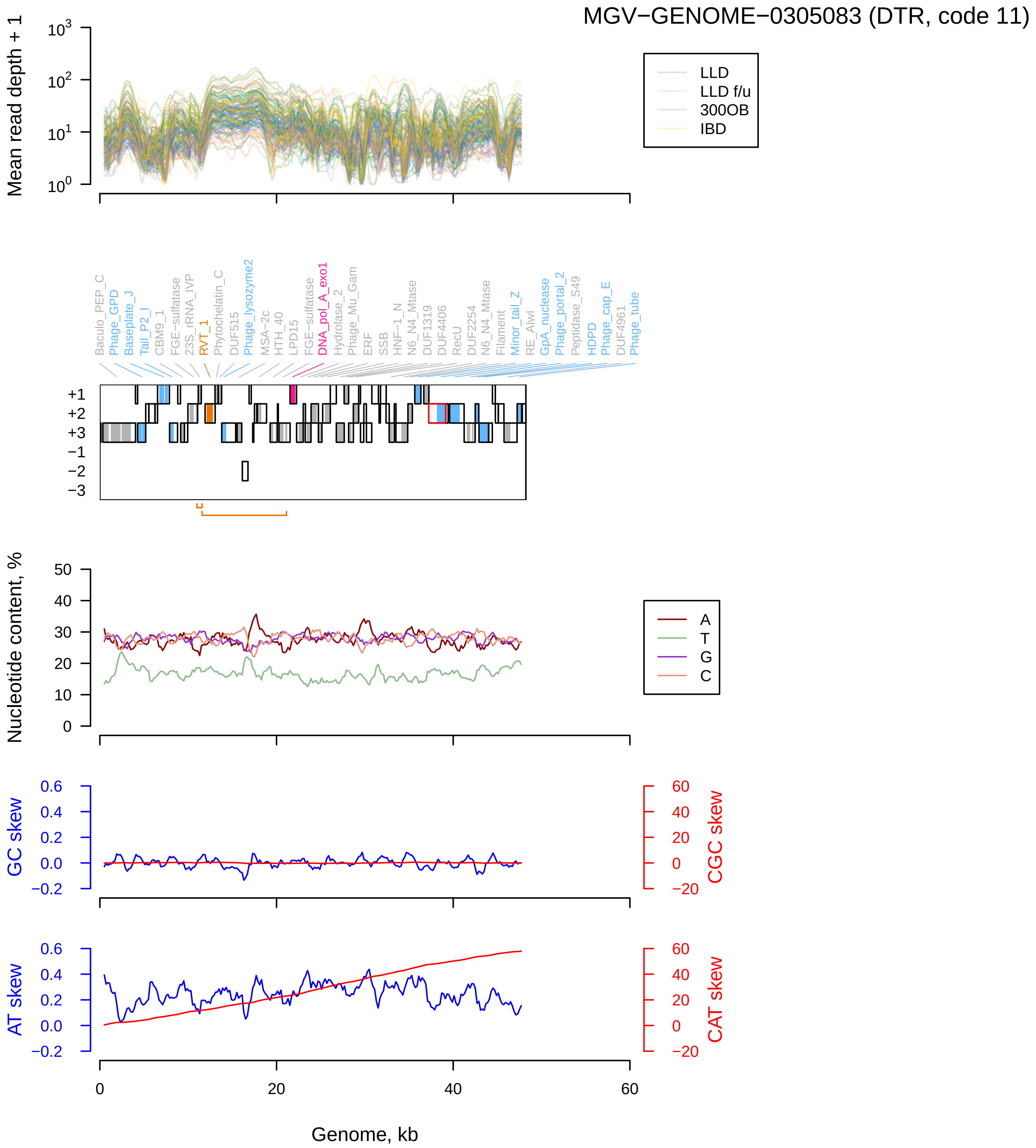

MGV−GENOME−0305083 (DTR, code 11)
Mean read depth + 1
LLD
LLD f/u
300OB
IBD
Baculo_PEP_C
Phage_GPD
Baseplate_J
Tail_P2_I
CBM9_1
FGE−sulfatase
23S_rRNA_IVP
RVT_1
Phytochelatin_C
DUF515
Phage_lysozyme2
MSA−2c
HTH_40
LPD15
FGE−sulfatase
DNA_pol_A_exo1
Hydrolase_2
Phage_Mu_Gam
ERF
SSB
HNF−1_N
N6_N4_Mtase
DUF1319
DUF4406
RecU
DUF2254
N6_N4_Mtase
Filament
Minor_tail_Z
RE_AlwI
GpA_nuclease
Phage_portal_2
Peptidase_S49
HDPD
Phage_cap_E
DUF4961
Phage_tube
Nucleotide content, %
A
T
G
C
GC skew
CGC skew
AT skew
CAT skew
Genome, kb

OMFQ01000250.1 (DTR, code 11)

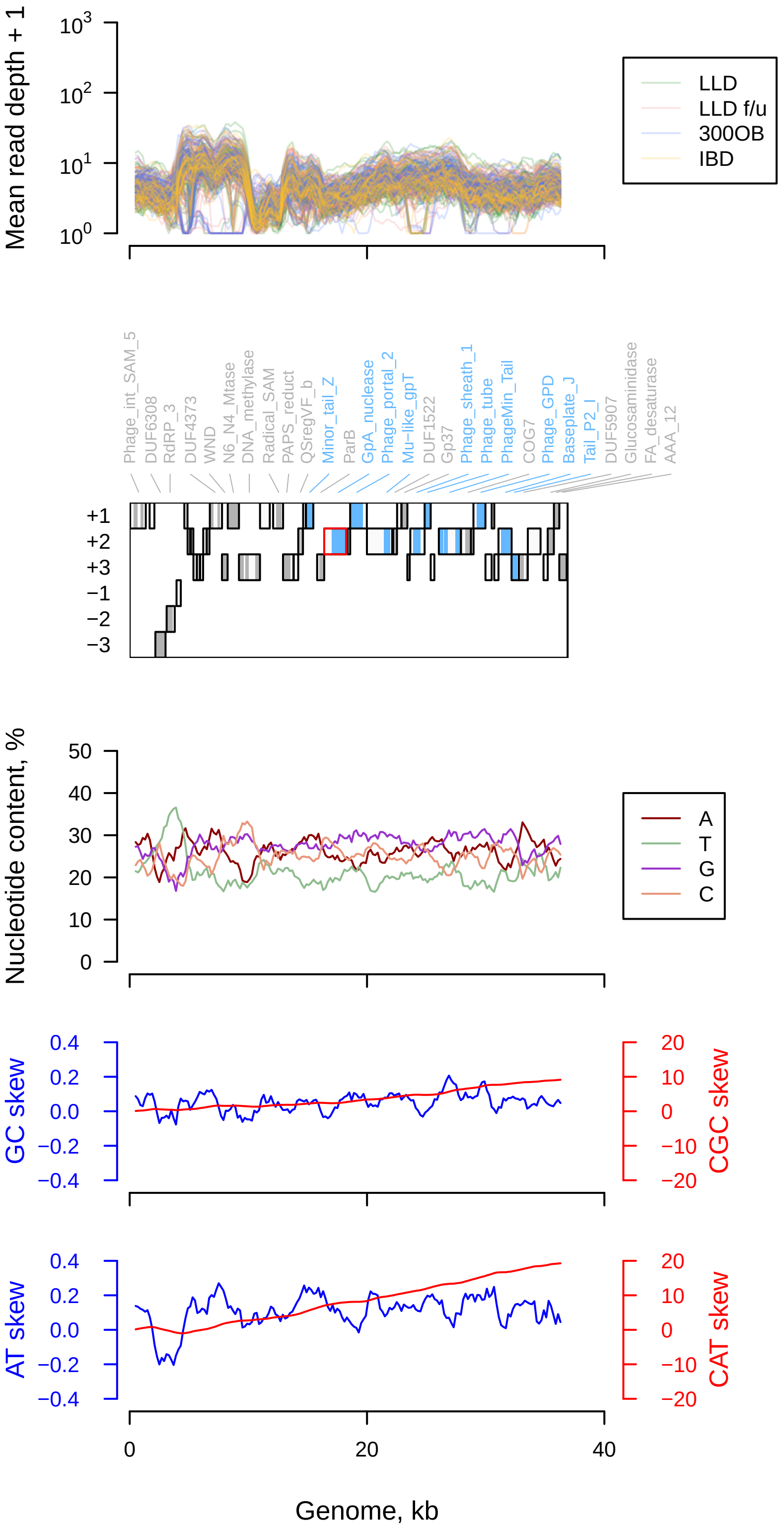

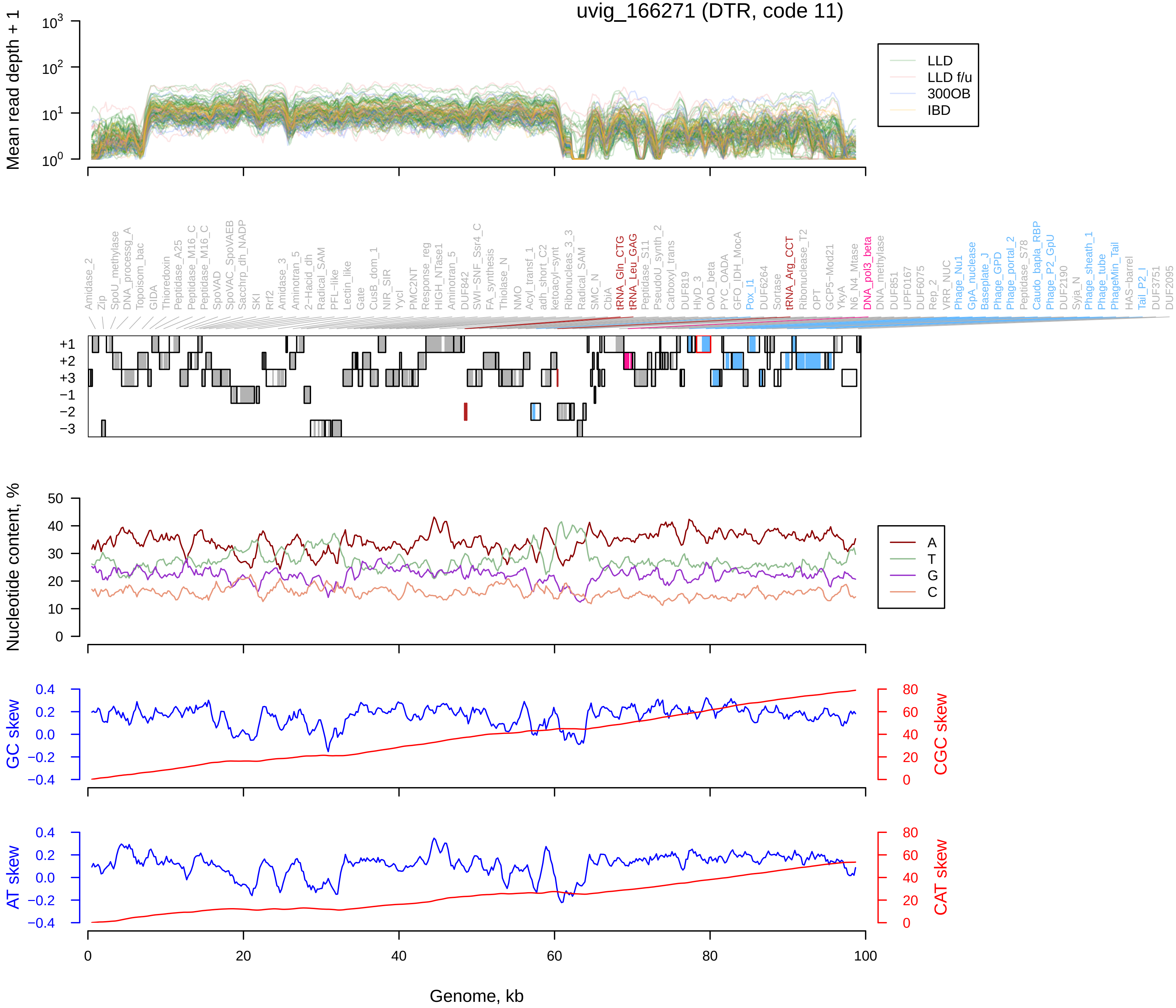

uvig_166271 (DTR, code 11)
Mean read depth + 1
LLD
LLD f/u
300OB
IBD
Amidase_2
Zip
SpoU_methylase
DNA_processg_A
Topoisom_bac
GIDA
Thioredoxin
Peptidase_A25
Peptidase_M16_C
Peptidase_M16_C
SpoVAD
SpoVAC_SpoVAEB
Sacchrp_dh_NADP
SKI
Rrf2
Amidase_3
Aminotran_5
2-Hacid_dh
Radical_SAM
PFL-like
Lectin_like
Gate
CusB_dom_1
NIR_SIR
YycI
PMC2NT
Response_reg
HIGH_NTase1
Aminotran_5
DUF842
SWI-SNF_Ssr4_C
FA_synthesis
Thiolase_N
NMO
Acyl_transf_1
adh_short_C2
ketoacyl-synt
Ribonucleas_3_3
Radical_SAM
SMC_N
CbiA
tRNA_Gln_CTG
tRNA_Leu_GAG
Peptidase_S11
PseudoU_synth_2
Carboxyl_trans
DUF819
HlyD_3
OAD_beta
PYC_OADA
GFO_IDH_MocA
Pox_I1
DUF6264
Sortase
tRNA_Arg_CCT
Ribonuclease_T2
OPT
GCP5-Mod21
YkyA
N6_N4_Mtase
DNA_pol3_beta
DNA_methylase
DUF851
UPF0167
DUF6075
Rep_2
VRR_NUC
Phage_Nu1
GpA_nuclease
Baseplate_J
Phage_GPD
Phage_portal_2
Peptidase_S78
Caudo_bapla_RBP
Phage_P2_GpU
DUF2190
Syja_N
Phage_sheath_1
Phage_tube
PhageMin_Tail
HAS-barrel
Tail_P2_I
DUF3751
DUF2095
+1
+2
+3
−1
−2
−3
Nucleotide content, %
A
T
G
C
GC skew
CGC skew
AT skew
CAT skew
Genome, kb

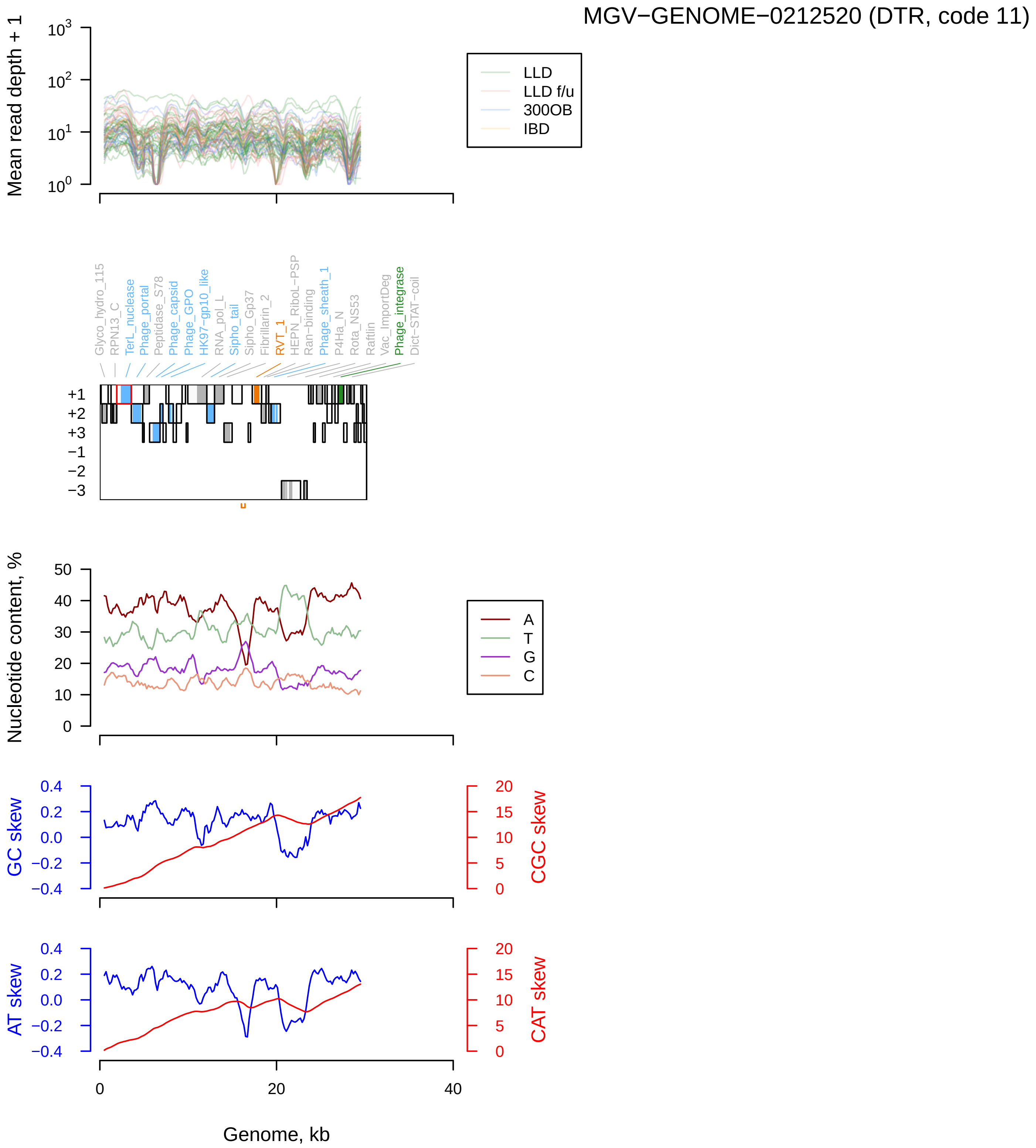

MGV−GENOME−0212520 (DTR, code 11)
Mean read depth + 1
LLD
LLD f/u
300OB
IBD
Glyco_hydro_115
RPN13_C
TerL_nuclease
Phage_portal
Peptidase_S78
Phage_capsid
Phage_GPO
HK97−gp10_like
RNA_pol_L
Sipho_tail
Sipho_Gp37
Fibrillarin_2
RVT_1
HEPN_RiboL−PSP
Ran−binding
Phage_sheath_1
P4Ha_N
Rota_NS53
Raftlin
Vac_ImportDeg
Phage_integrase
Dict−STAT−coil
Nucleotide content, %
A
T
G
C
GC skew
CGC skew
AT skew
CAT skew
Genome, kb

Han_2018_ERR1398212_NODE_689_length_32728_cov_10.495210 (DTR, code 11)

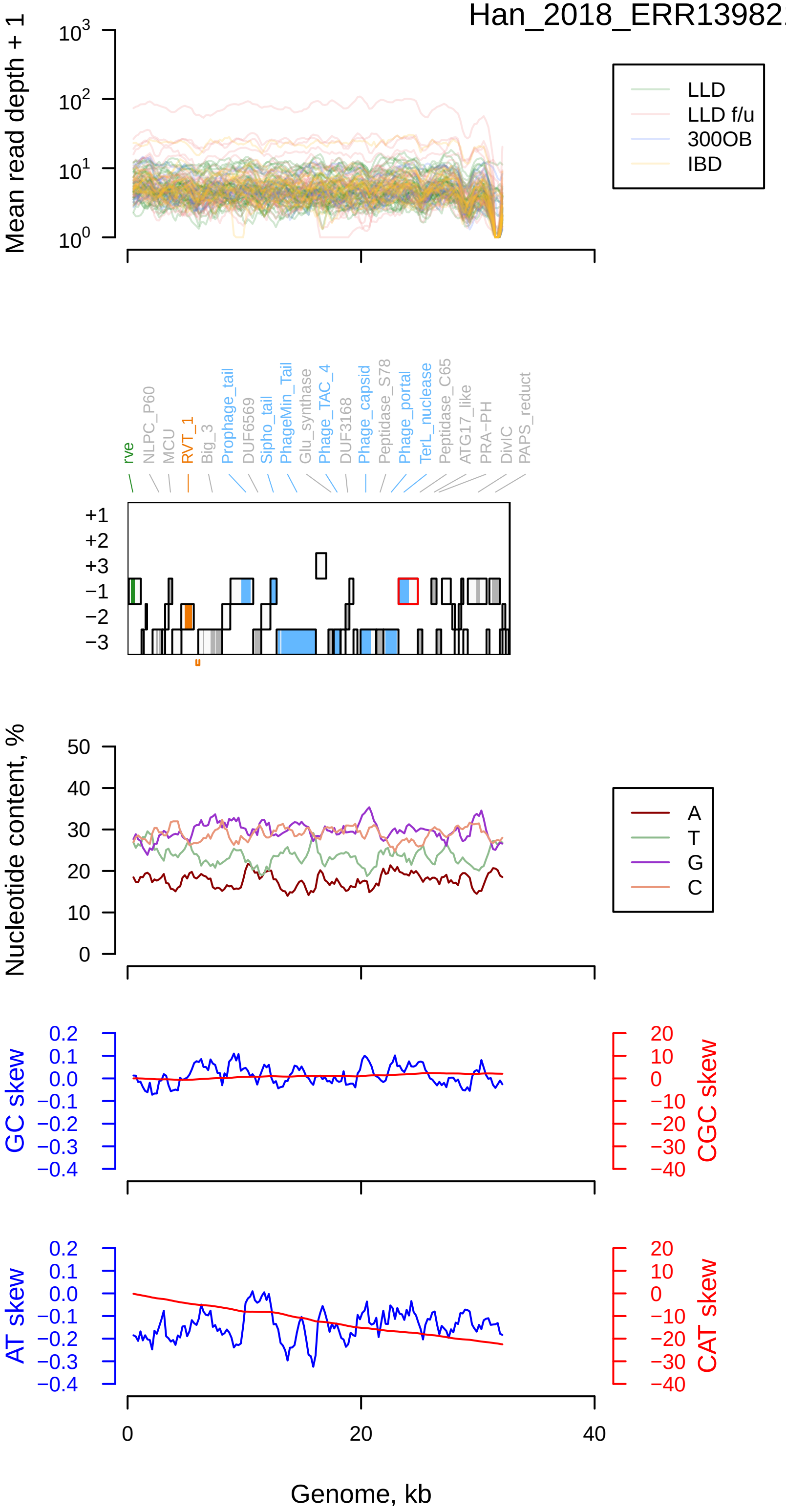

MGV−GENOME−0221889 (DTR, code 11)

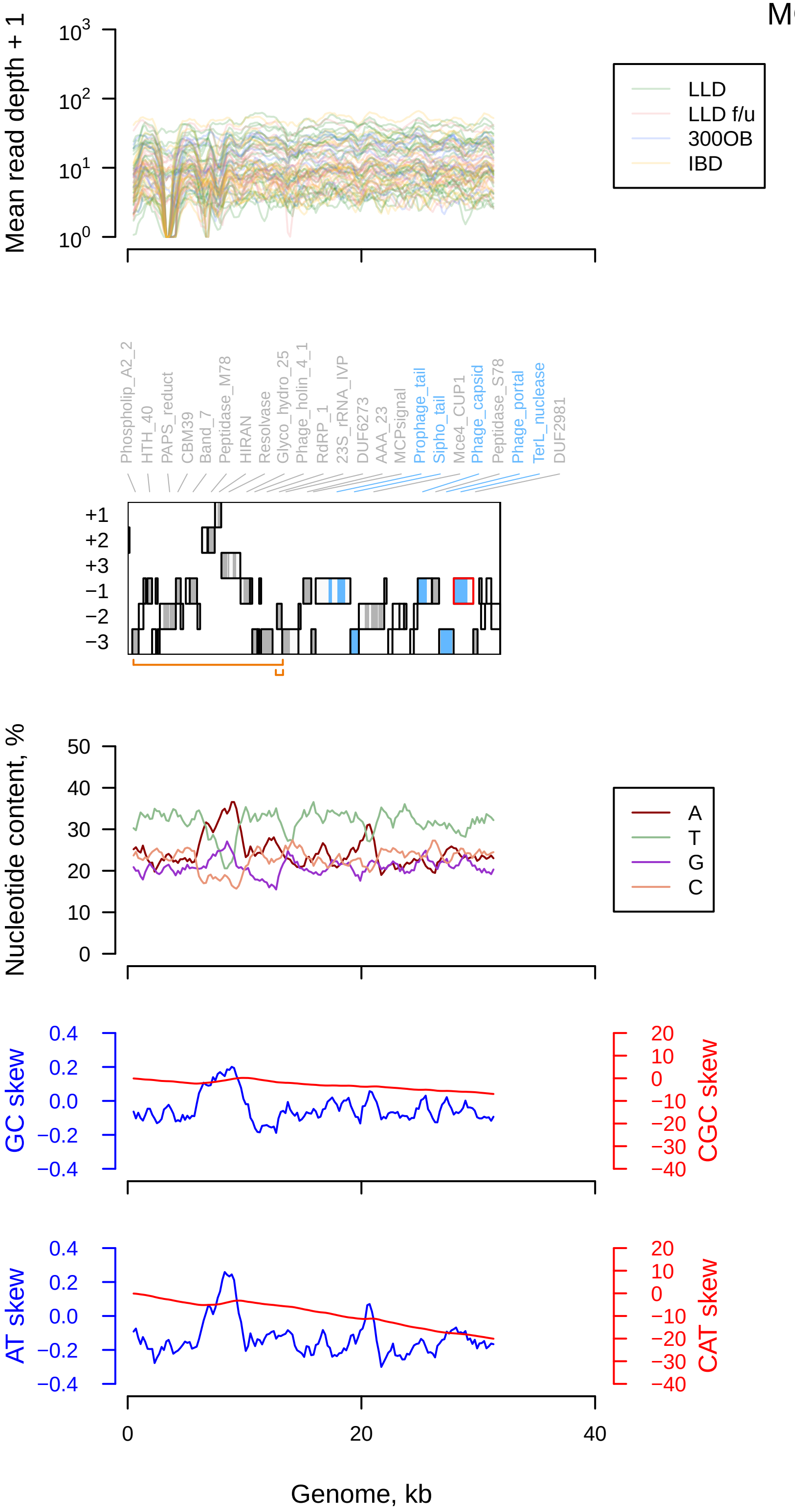

MGV−GENOME−0163922 (ITR, code 11)

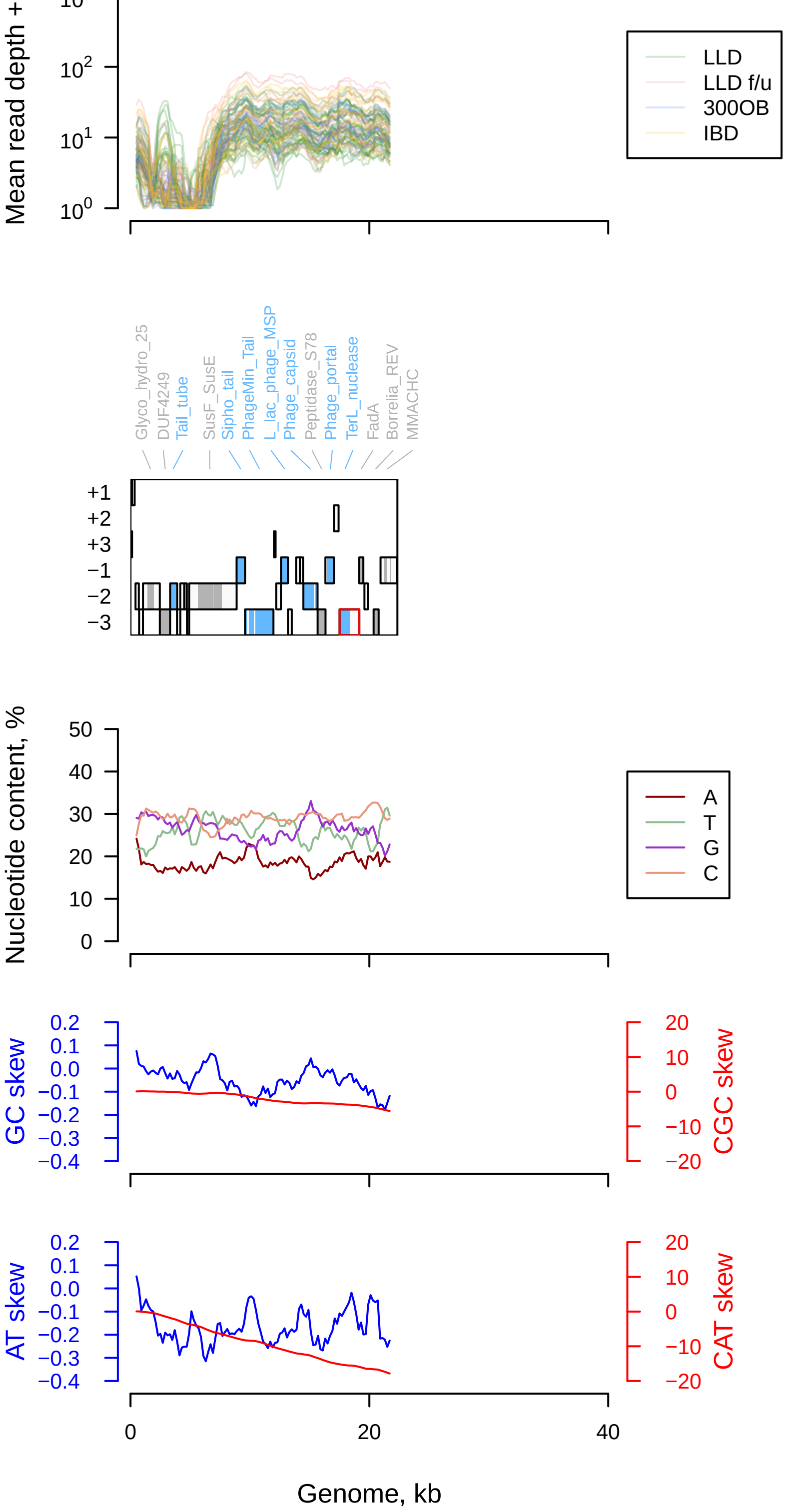

MGV−GENOME−0244886 (DTR, code 15)

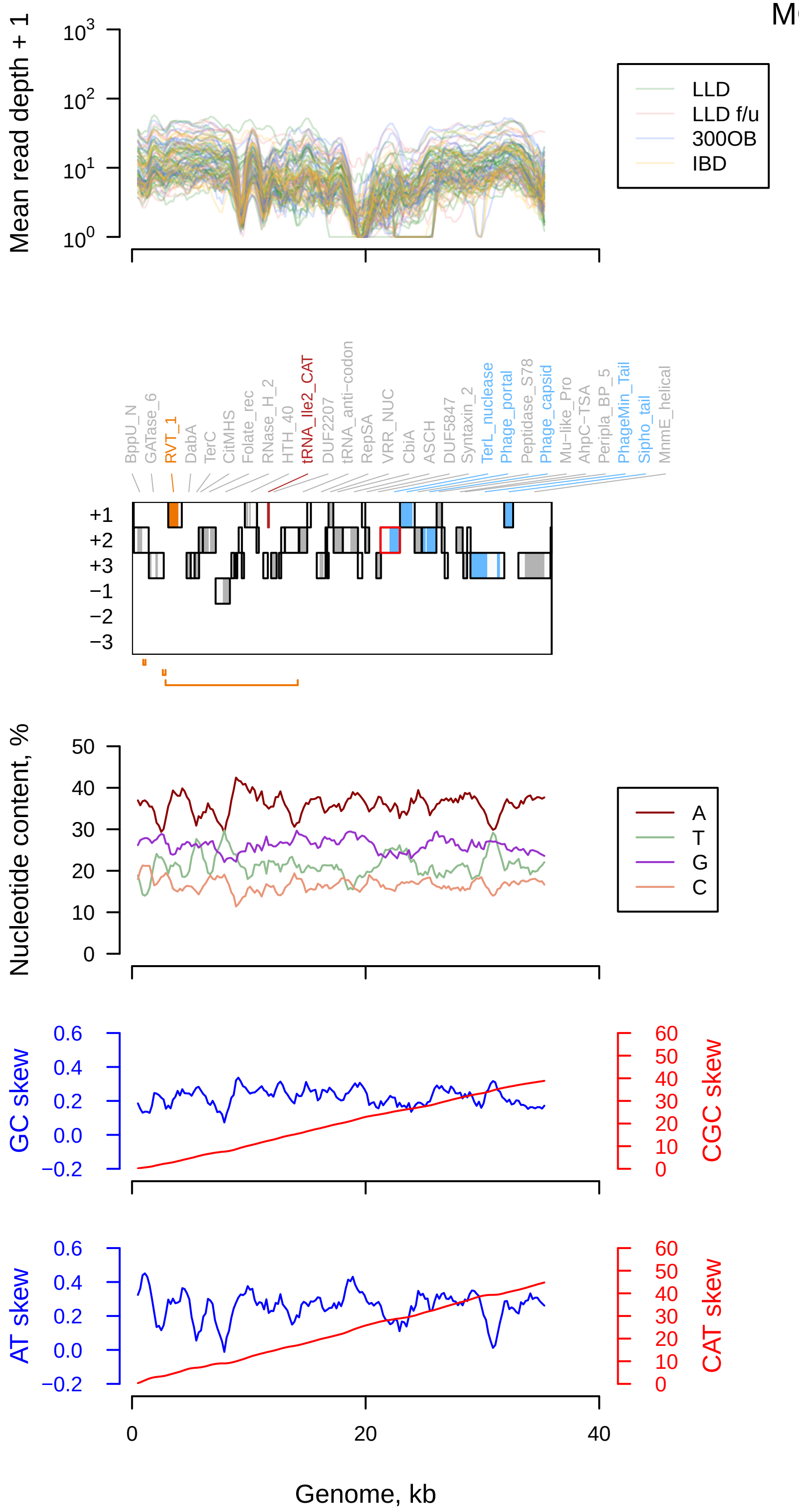

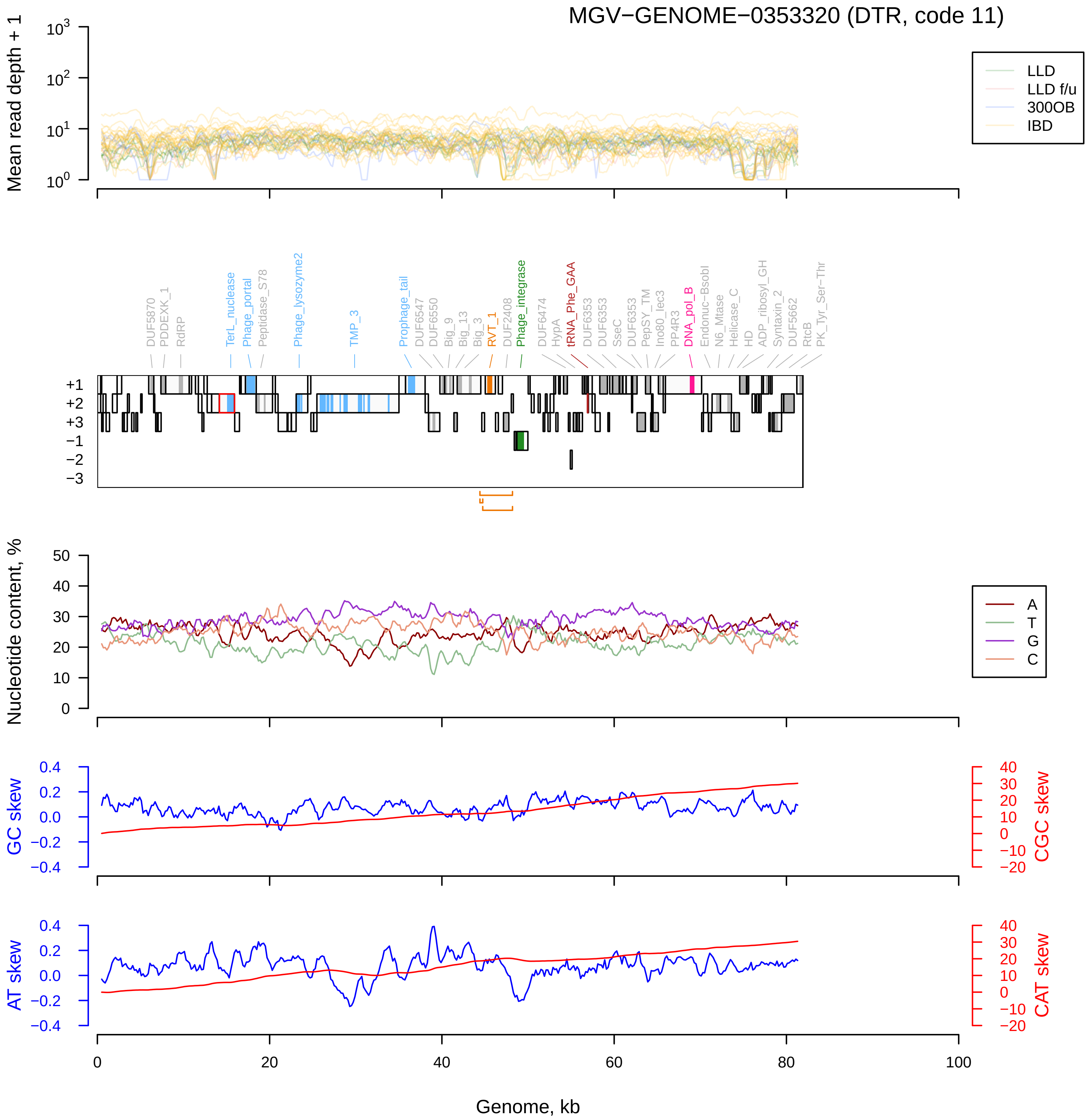
MGV−GENOME−0353320 (DTR, code 11)
Mean read depth + 1
LLD
LLD f/u
300OB
IBD
DUF5870
PDDEXK_1
RdRP
TerL_nuclease
Phage_portal
Peptidase_S78
Phage_lysozyme2
TMP_3
Prophage_tail
DUF6547
DUF6550
Big_9
Big_13
Big_3
RVT_1
DUF2408
Phage_integrase
DUF6474
HypA
tRNA_Phe_GAA
DUF6353
DUF6353
SseC
DUF6353
PepSY_TM
Ino80_Iec3
PP4R3
DNA_pol_B
Endonuc−BsobI
N6_Mtase
Helicase_C
HD
ADP_ribosyl_GH
Syntaxin_2
DUF5662
RtcB
PK_Tyr_Ser−Thr
Nucleotide content, %
A
T
G
C
GC skew
CGC skew
AT skew
CAT skew
Genome, kb

uvig_205988 (DTR, code 11)

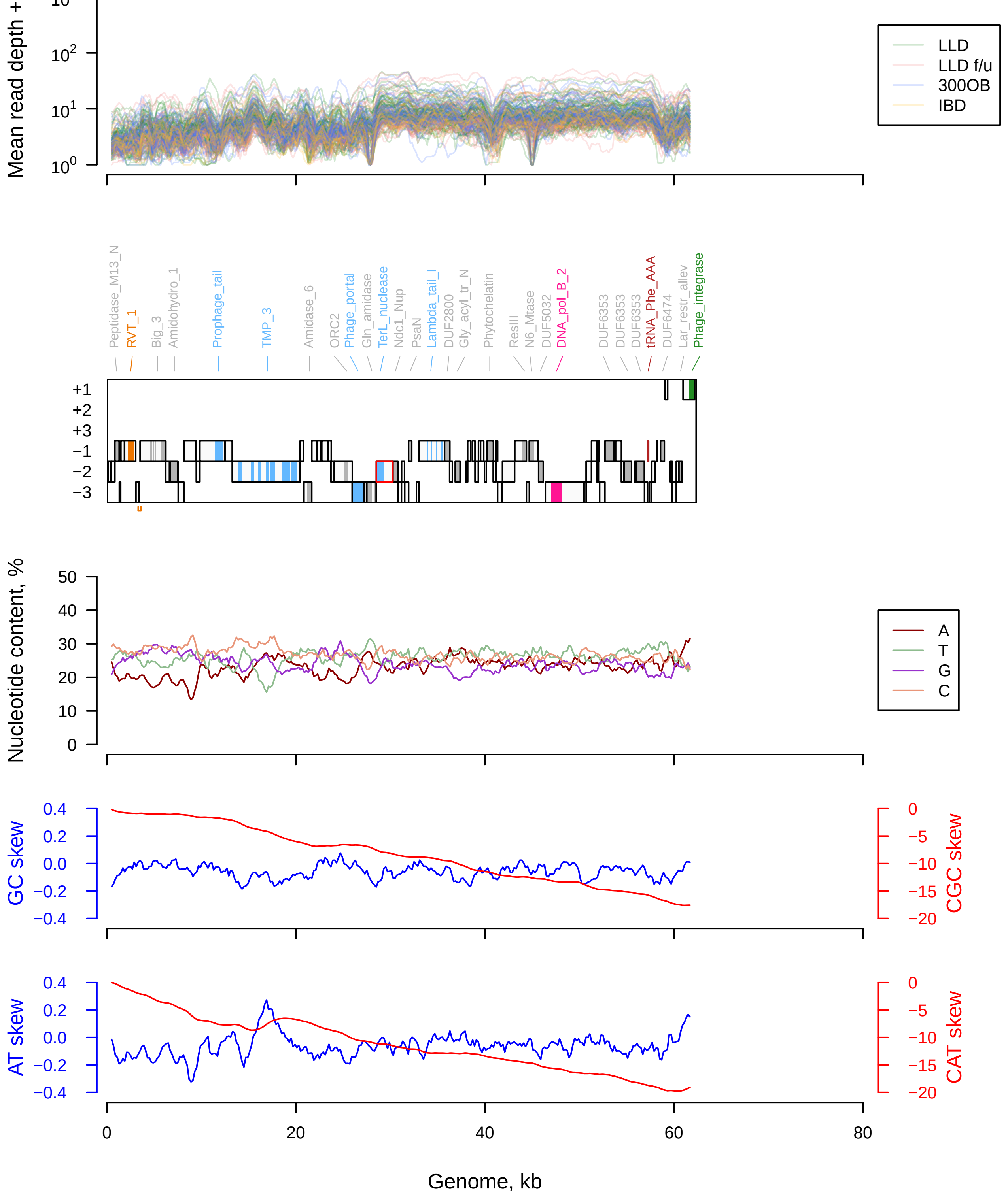

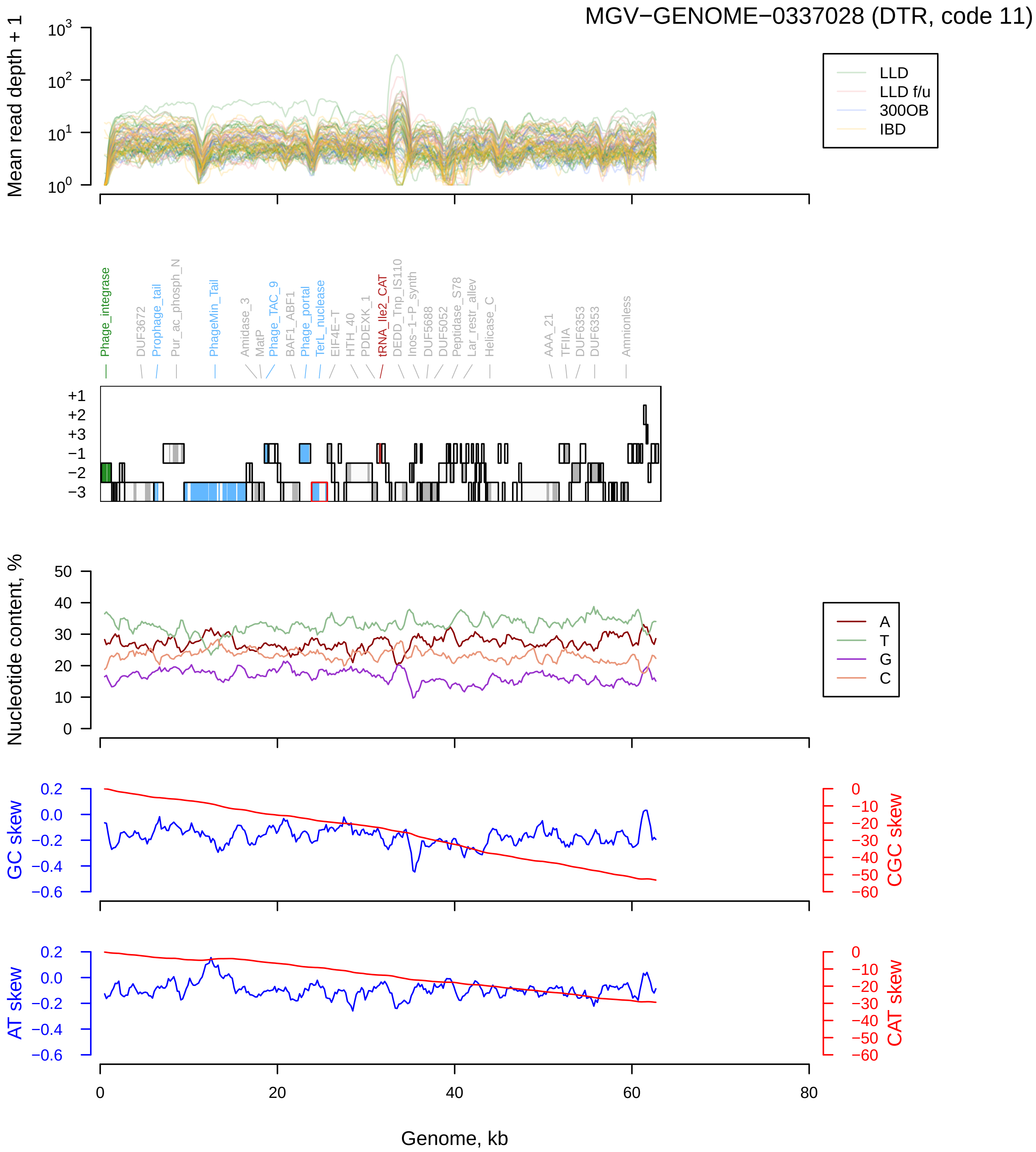
MGV−GENOME−0337028 (DTR, code 11)
Mean read depth + 1
LLD
LLD f/u
300OB
IBD
Phage_integrase
DUF3672
Prophage_tail
Pur_ac_phosph_N
PhageMin_Tail
Amidase_3
MatP
Phage_TAC_9
BAF1_ABF1
Phage_portal
TerL_nuclease
EIF4E-T
HTH_40
PDDEXK_1
tRNA_Ile2_CAT
DEDD_Tnp_IS110
Inos-1-P_synth
DUF5688
DUF5052
Peptidase_S78
Lar_restr_allev
Helicase_C
AAA_21
TFIIA
DUF6353
DUF6353
Amnionless
Nucleotide content, %
A
T
G
C
GC skew
CGC skew
AT skew
CAT skew
Genome, kb

MGV−GENOME−0138828 (ITR, code 11)

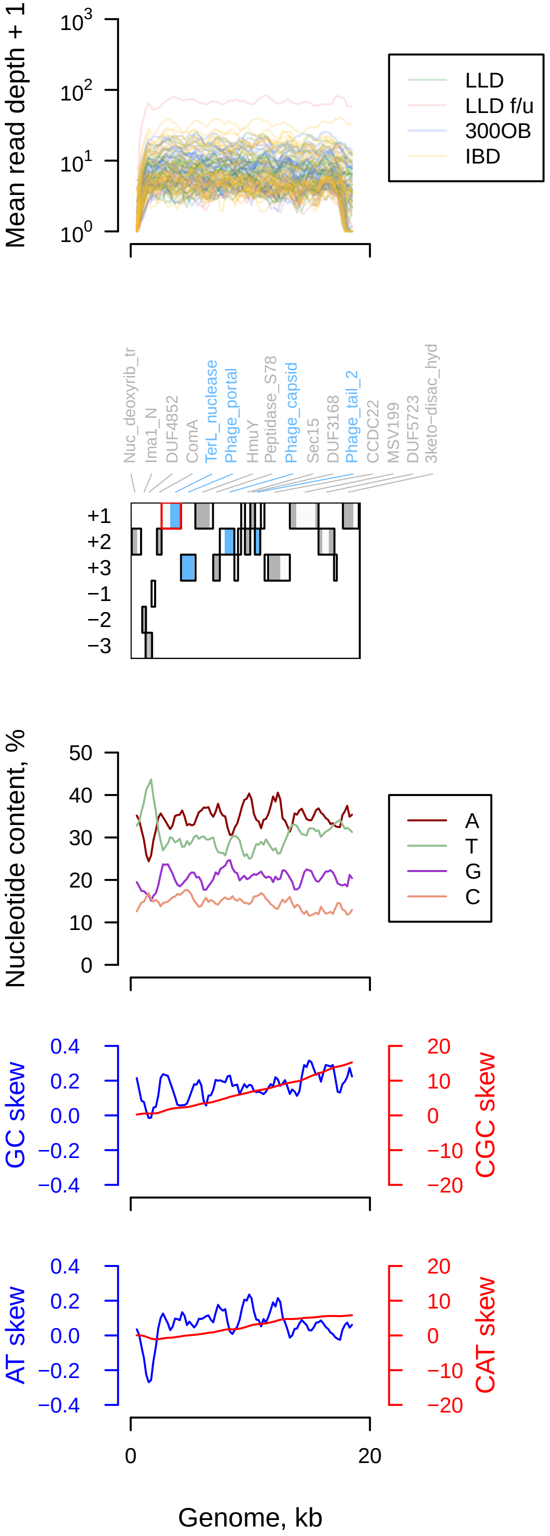

MGV−GENOME−0109615 (ITR, code 11)

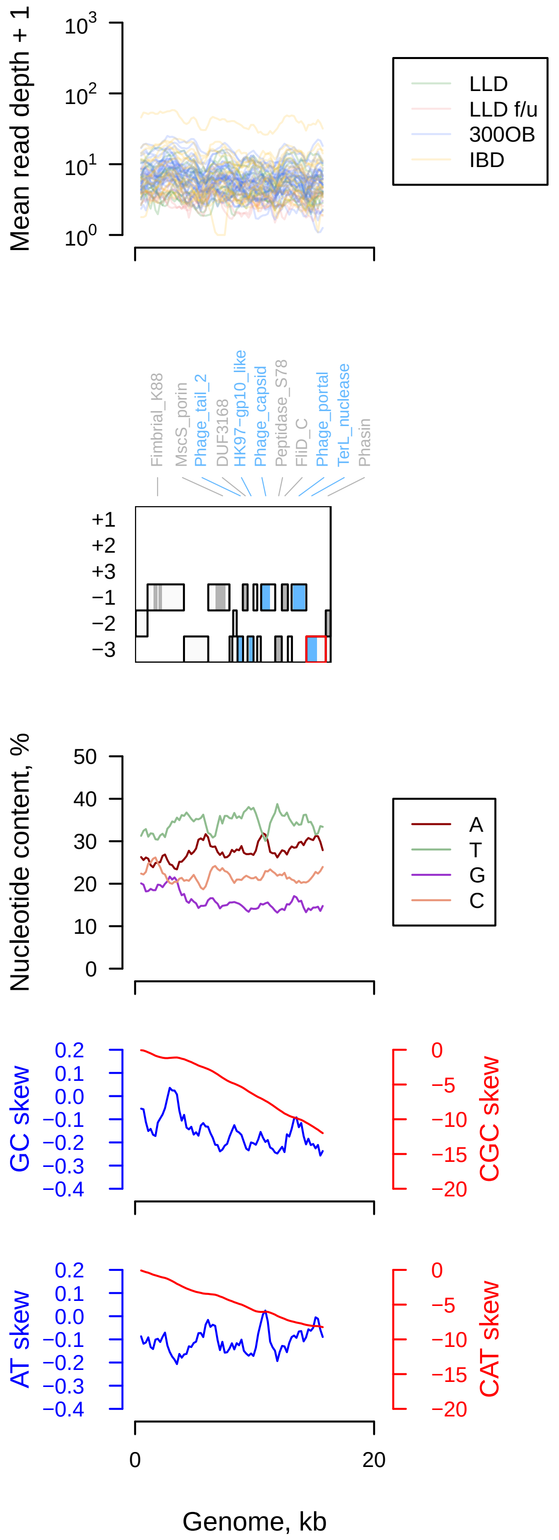

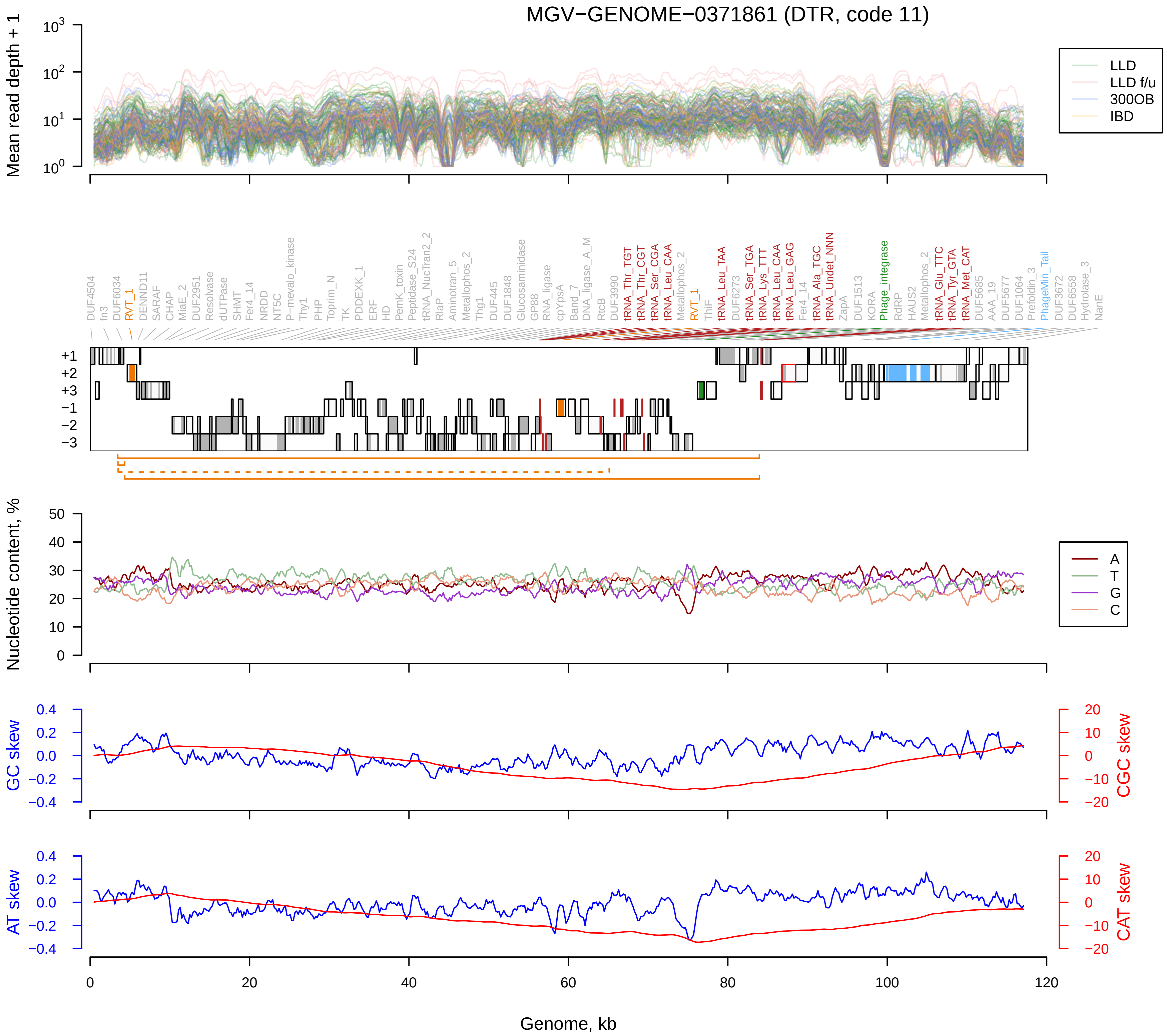
MGV−GENOME−0371861 (DTR, code 11)
Mean read depth + 1
LLD
LLD f/u
300OB
IBD
DUF4504
fn3
DUF6034
RVT_1
DENND11
SARAF
CHAP
MiaE_2
DUF2951
Resolvase
dUTPase
SHMT
Fer4_14
NRDD
NT5C
P−mevalo_kinase
Thy1
PHP
Toprim_N
TK
PDDEXK_1
ERF
HD
PemK_toxin
Peptidase_S24
tRNA_NucTran2_2
RlaP
Aminotran_5
Metallophos_2
Thg1
DUF445
DUF1848
Glucosaminidase
GP88
RNA_ligase
cpYpsA
Band_7
DNA_ligase_A_M
RtcB
DUF3990
tRNA_Thr_TGT
tRNA_Thr_CGT
tRNA_Ser_CGA
tRNA_Leu_CAA
Metallophos_2
RVT_1
ThiF
tRNA_Leu_TAA
DUF6273
tRNA_Ser_TGA
tRNA_Lys_TTT
tRNA_Leu_CAA
tRNA_Leu_GAG
Fer4_14
tRNA_Ala_TGC
tRNA_Undet_NNN
ZapA
DUF1513
KORA
Phage_integrase
RdRP
HAUS2
Metallophos_2
tRNA_Glu_TTC
tRNA_Tyr_GTA
tRNA_Met_CAT
DUF5685
AAA_19
DUF5677
DUF1064
Prefoldin_3
PhageMin_Tail
DUF3672
DUF6558
Hydrolase_3
NanE
+1
+2
+3
−1
−2
−3
Nucleotide content, %
A
T
G
C
GC skew
CGC skew
AT skew
CAT skew
Genome, kb

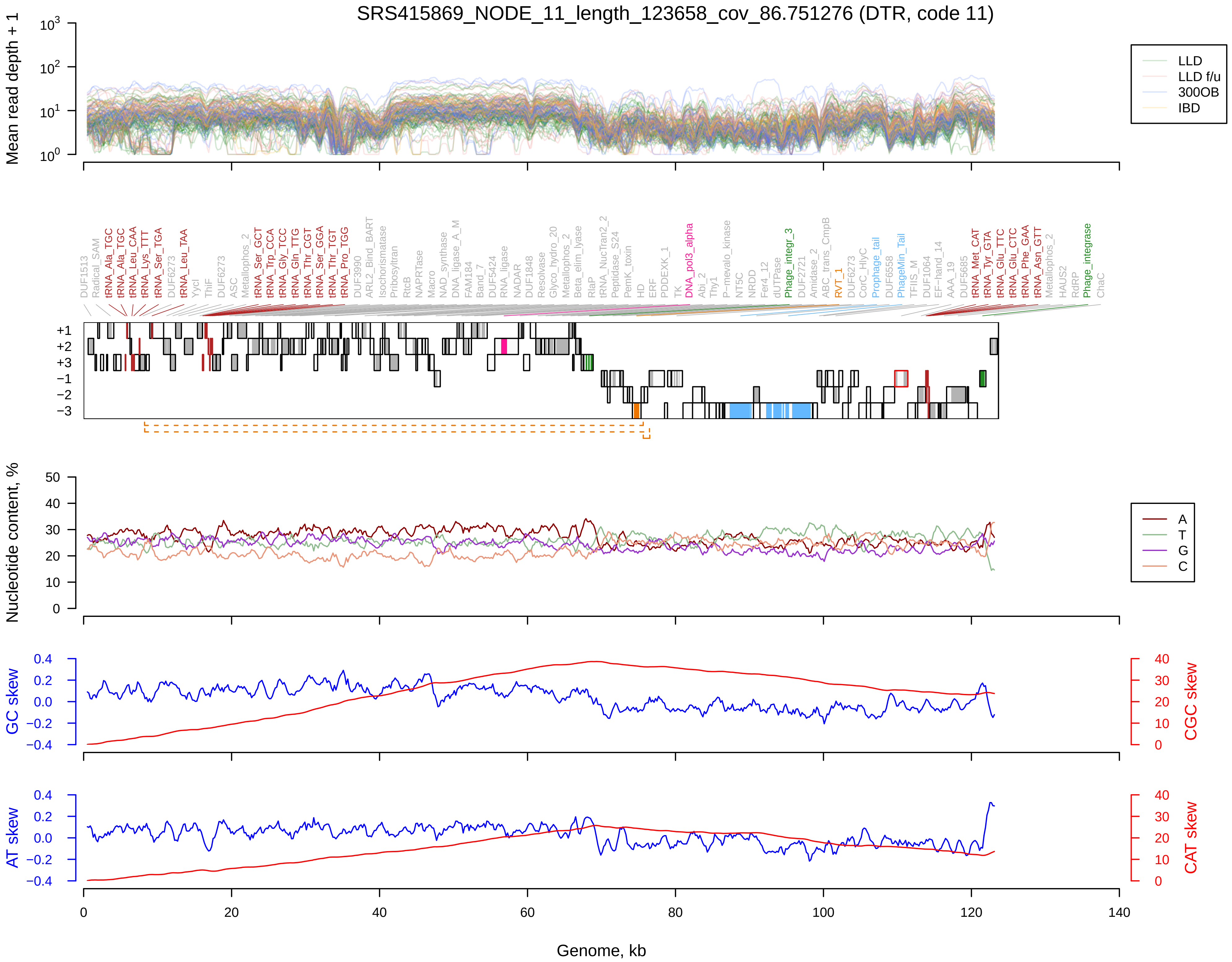
SRS415869_NODE_11_length_123658_cov_86.751276 (DTR, code 11)
Mean read depth + 1
LLD
LLD f/u
300OB
IBD
DUF1513
Radical_SAM
tRNA_Ala_TGC
tRNA_Ala_TGC
tRNA_Leu_CAA
tRNA_Lys_TTT
tRNA_Ser_TGA
DUF6273
tRNA_Leu_TAA
YycI
ThiF
DUF6273
ASC
Metallophos_2
tRNA_Ser_GCT
tRNA_Trp_CCA
tRNA_Gly_TCC
tRNA_Gln_TTG
tRNA_Thr_CGT
tRNA_Ser_GGA
tRNA_Thr_TGT
tRNA_Pro_TGG
DUF3990
ARL2_Bind_BART
Isochorismatase
Pribosyltran
RtcB
NAPRTase
Macro
NAD_synthase
DNA_ligase_A_M
FAM184
Band_7
DUF5424
RNA_ligase
NADAR
DUF1848
Resolvase
Glyco_hydro_20
Metallophos_2
Beta_elim_lyase
RlaP
tRNA_NucTran2_2
Peptidase_S24
PemK_toxin
HD
ERF
PDDEXK_1
TK
DNA_pol3_alpha
Abi_2
Thy1
P-mevalo_kinase
NT5C
NRDD
Fer4_12
dUTPase
Phage_integr_3
DUF2721
Amidase_2
ABC_trans_CmpB
RVT_1
DUF6273
CorC_HlyC
Prophage_tail
DUF6558
PhageMin_Tail
TFIIS_M
DUF1064
EF-hand_14
AAA_19
DUF5685
tRNA_Met_CAT
tRNA_Tyr_GTA
tRNA_Glu_TTC
tRNA_Glu_CTC
tRNA_Phe_GAA
tRNA_Asn_GTT
Metallophos_2
HAUS2
RdRP
Phage_integrase
ChaC
+1
+2
+3
−1
−2
−3
Nucleotide content, %
A
T
G
C
GC skew
CGC skew
AT skew
CAT skew
Genome, kb
0
20
40
60
80
100
120
140

Manrique_Person1_NODE_54_length_54658_cov_29.442924 (DTR, code 11)

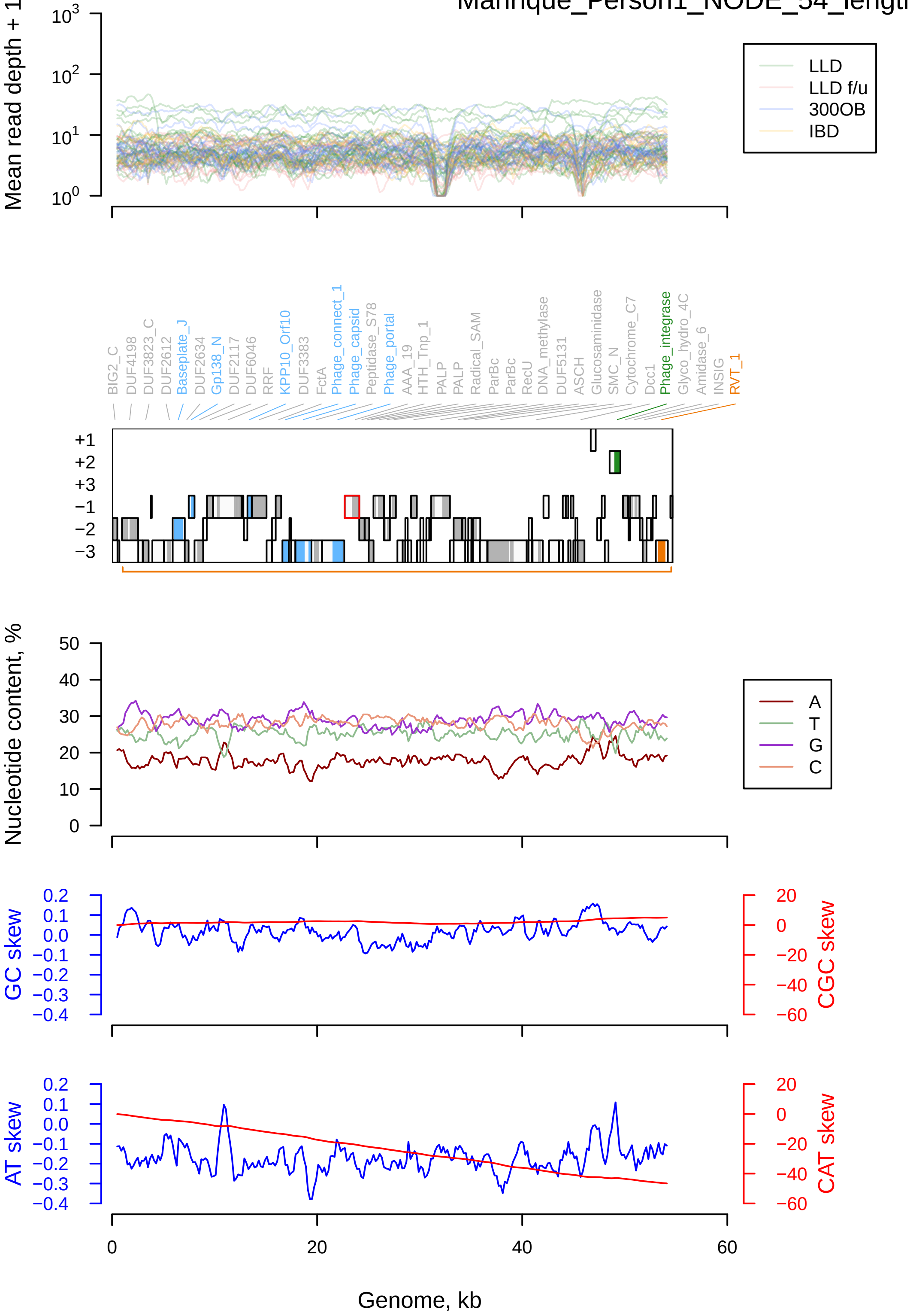

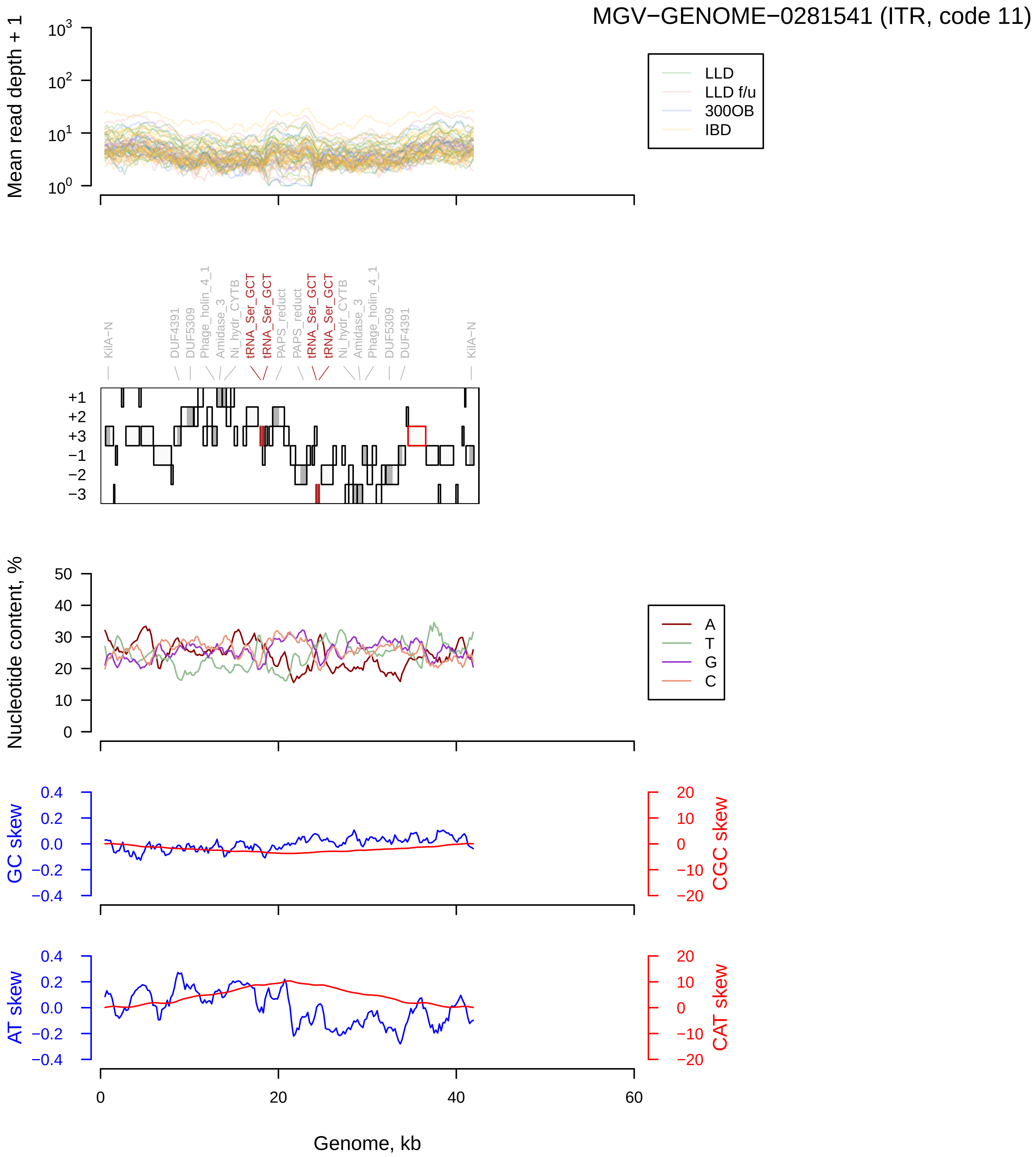

MGV−GENOME−0281541 (ITR, code 11)
Mean read depth + 1
LLD
LLD f/u
300OB
IBD
KilA−N
DUF4391
DUF5309
Phage_holin_4_1
Amidase_3
Ni_hydr_CYTB
tRNA_Ser_GCT
tRNA_Ser_GCT
PAPS_reduct
PAPS_reduct
tRNA_Ser_GCT
tRNA_Ser_GCT
Ni_hydr_CYTB
Amidase_3
Phage_holin_4_1
DUF5309
DUF4391
KilA−N
Nucleotide content, %
A
T
G
C
GC skew
CGC skew
AT skew
CAT skew
Genome, kb

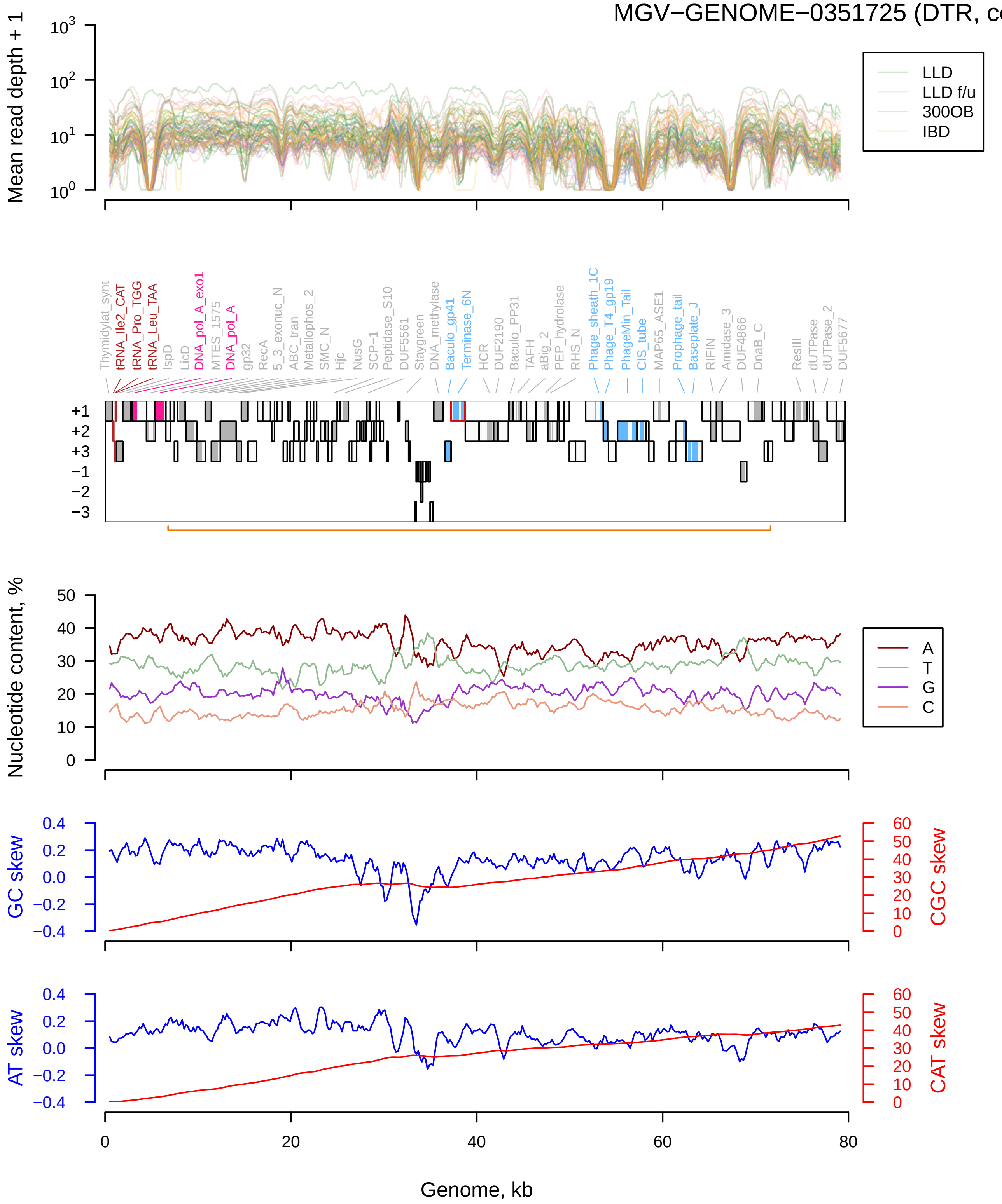

MGV−GENOME−0351725 (DTR, code 11)
Mean read depth + 1
LLD
LLD f/u
300OB
IBD
Thymidylat_synt
tRNA_Ile2_CAT
tRNA_Pro_TGG
tRNA_Leu_TAA
IspD
LicD
DNA_pol_A_exo1
MTES_1575
DNA_pol_A
gp32
RecA
5_3_exonuc_N
ABC_tran
Metallophos_2
SMC_N
Hjc
NusG
SCP-1
Peptidase_S10
DUF5561
Staygreen
DNA_methylase
Baculo_gp41
Terminase_6N
HCR
DUF2190
Baculo_PP31
TAFH
aBig_2
PEP_hydrolase
RHS_N
Phage_sheath_1C
Phage_T4_gp19
PhageMin_Tail
CIS_tube
MAP65_ASE1
Prophage_tail
Baseplate_J
RIFIN
Amidase_3
DUF4866
DnaB_C
ResIII
dUTPase
dUTPase_2
DUF5677
Nucleotide content, %
A
T
G
C
GC skew
CGC skew
AT skew
CAT skew
Genome, kb

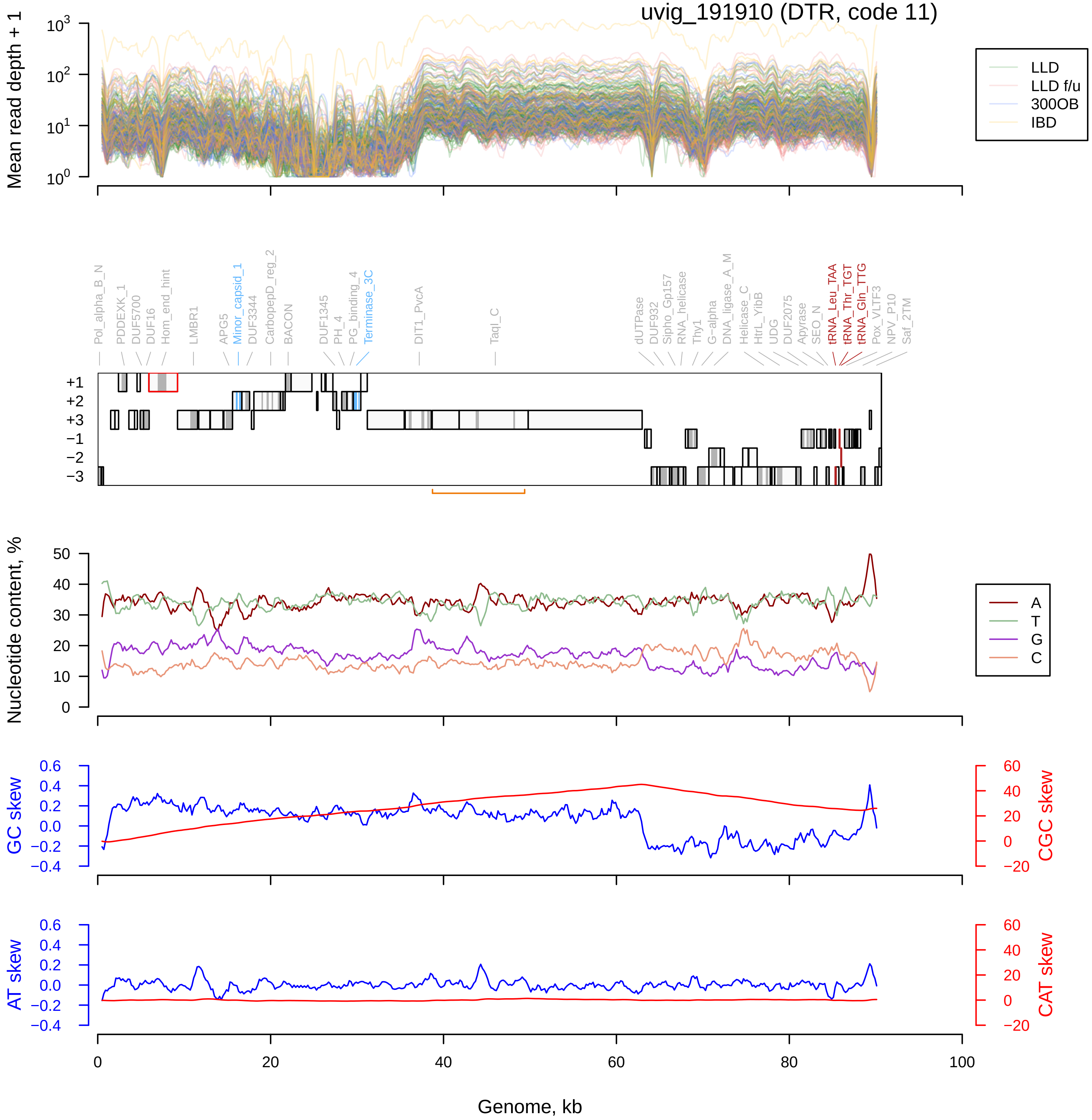
uvig_191910 (DTR, code 11)
Mean read depth + 1
LLD
LLD f/u
300OB
IBD
Pol_alpha_B_N
PDDEXK_1
DUF5700
DUF16
Hom_end_hint
LMBR1
APG5
Minor_capsid_1
DUF3344
CarbopepD_reg_2
BACON
DUF1345
PH_4
PG_binding_4
Terminase_3C
DIT1_PvcA
TaqI_C
dUTPase
DUF932
Sipho_Gp157
RNA_helicase
Thy1
G-alpha
DNA_ligase_A_M
Helicase_C
HtrL_YibB
UDG
DUF2075
Apyrase
SEO_N
tRNA_Leu_TAA
tRNA_Thr_TGT
tRNA_Gln_TTG
Pox_VLTF3
NPV_P10
Saf_2TM
+1
+2
+3
−1
−2
−3
Nucleotide content, %
A
T
G
C
GC skew
CGC skew
AT skew
CAT skew
Genome, kb

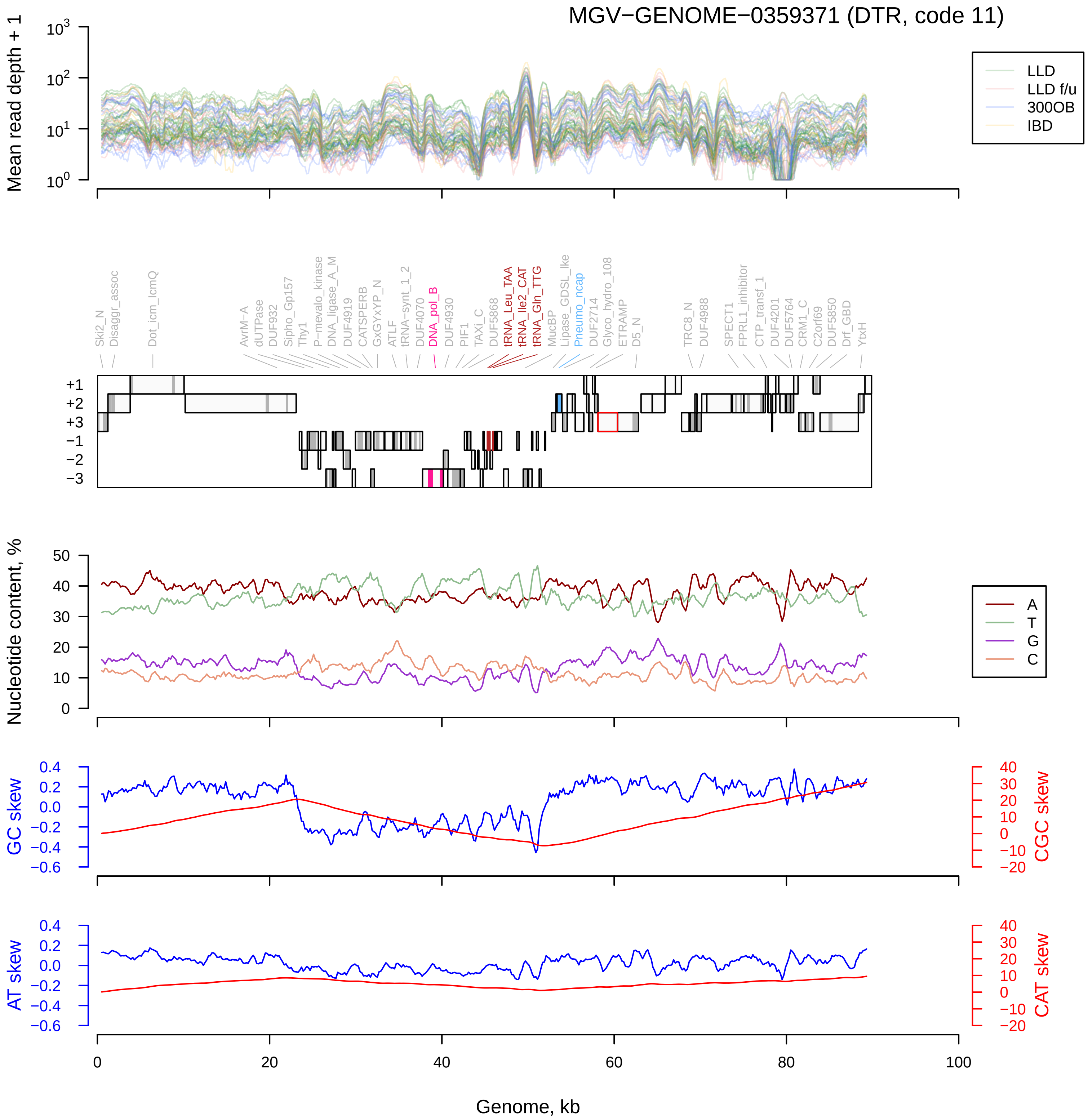

MGV−GENOME−0359371 (DTR, code 11)
Mean read depth + 1
LLD
LLD f/u
300OB
IBD
Ski2_N
Disaggr_assoc
Dot_icm_IcmQ
AvrM−A
dUTPase
DUF932
Sipho_Gp157
Thy1
P−mevalo_kinase
DNA_ligase_A_M
DUF4919
CATSPERB
GxGYxYP_N
ATLF
tRNA−synt_1_2
DUF4070
DNA_pol_B
DUF4930
PIF1
TAXi_C
DUF5868
tRNA_Leu_TAA
tRNA_Ile2_CAT
tRNA_Gln_TTG
MucBP
Lipase_GDSL_lke
Pneumo_ncap
DUF2714
Glyco_hydro_108
ETRAMP
D5_N
TRC8_N
DUF4988
SPECT1
FPRL1_inhibitor
CTP_transf_1
DUF4201
DUF5764
CRM1_C
C2orf69
DUF5850
Drf_GBD
YtxH
Nucleotide content, %
A
T
G
C
GC skew
CGC skew
AT skew
CAT skew
Genome, kb

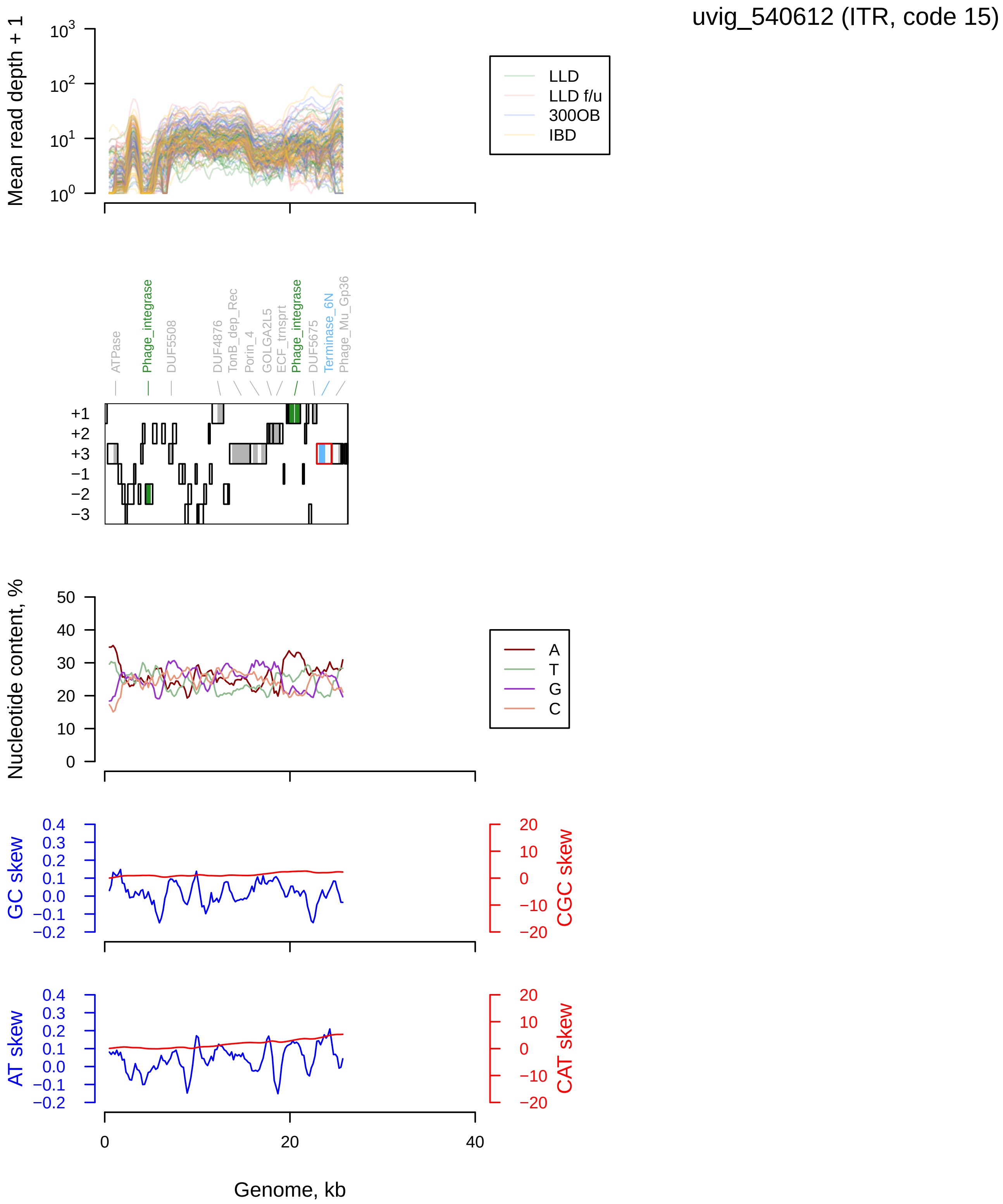
uvig_540612 (ITR, code 15)
Mean read depth + 1
LLD
LLD f/u
300OB
IBD
ATPase
Phage_integrase
DUF5508
DUF4876
TonB_dep_Rec
Porin_4
GOLGA2L5
ECF_trnsprt
Phage_integrase
DUF5675
Terminase_6N
Phage_Mu_Gp36
Nucleotide content, %
A
T
G
C
GC skew
CGC skew
AT skew
CAT skew
Genome, kb

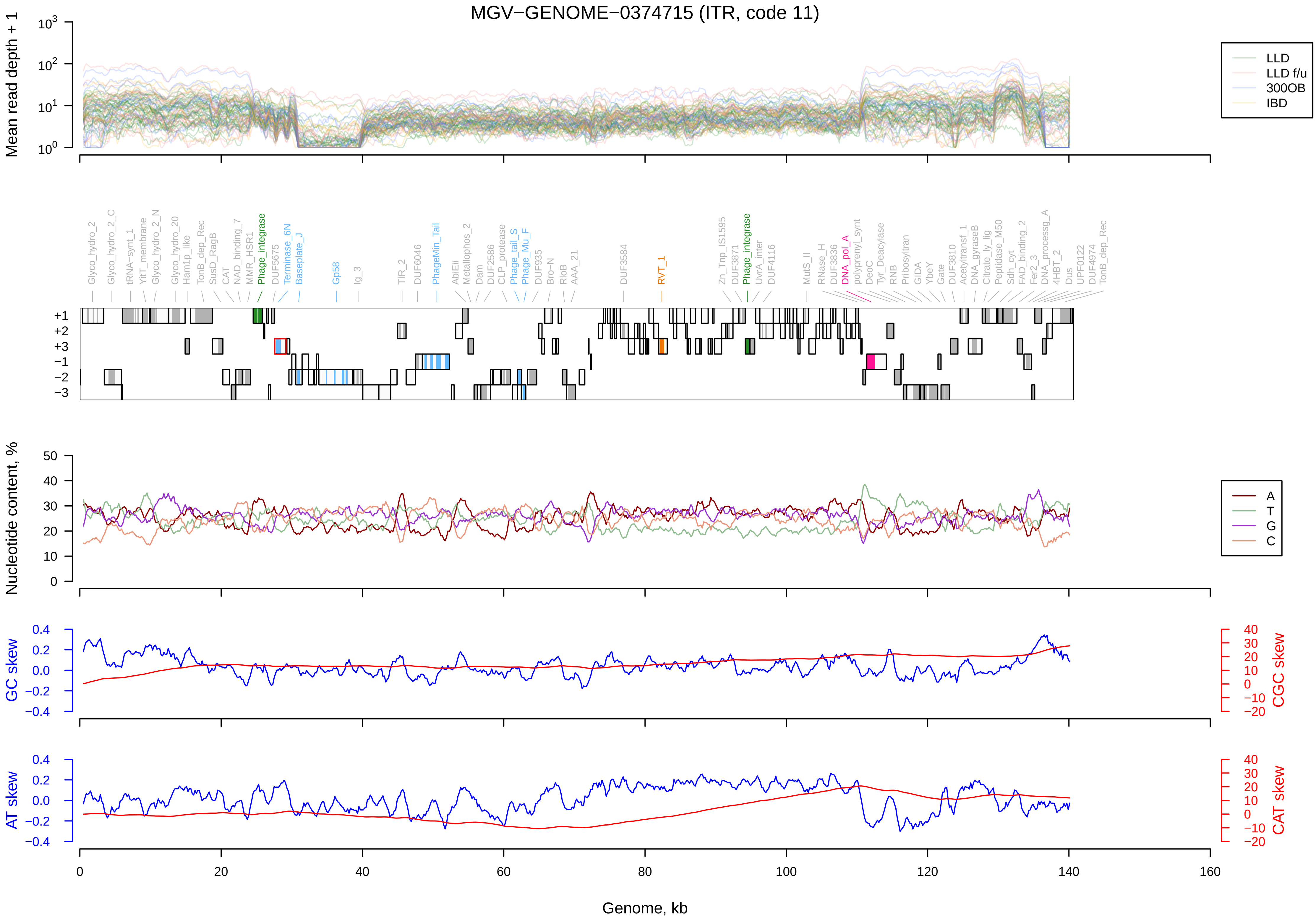

MGV−GENOME−0374715 (ITR, code 11)
Mean read depth + 1
LLD
LLD f/u
300OB
IBD
Nucleotide content, %
A
T
G
C
GC skew
CGC skew
AT skew
CAT skew
Genome, kb

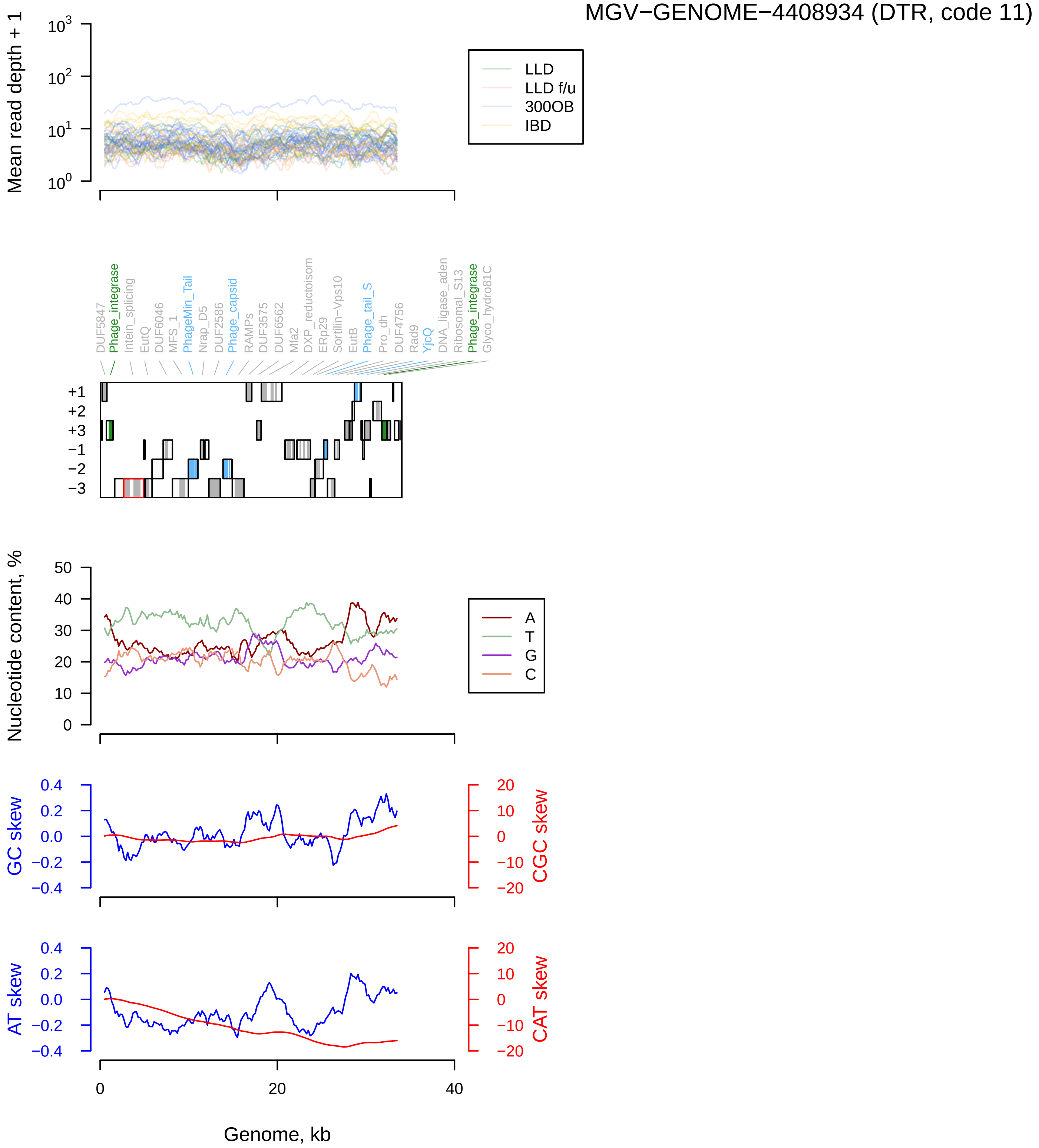

MGV−GENOME−4408934 (DTR, code 11)
Mean read depth + 1
LLD
LLD f/u
300OB
IBD
DUF5847
Phage_integrase
Intein_splicing
EutQ
DUF6046
MFS_1
PhageMin_Tail
Nrap_D5
DUF2586
Phage_capsid
RAMPs
DUF3575
DUF6562
Mfa2
DXP_reductoisom
ERp29
Sortilin−Vps10
EutB
Phage_tail_S
Pro_dh
DUF4756
Rad9
YjcQ
DNA_ligase_aden
Ribosomal_S13
Phage_integrase
Glyco_hydro81C
Nucleotide content, %
A
T
G
C
GC skew
CGC skew
AT skew
CAT skew
Genome, kb

MGV−GENOME−0310268 (DTR, code 11)

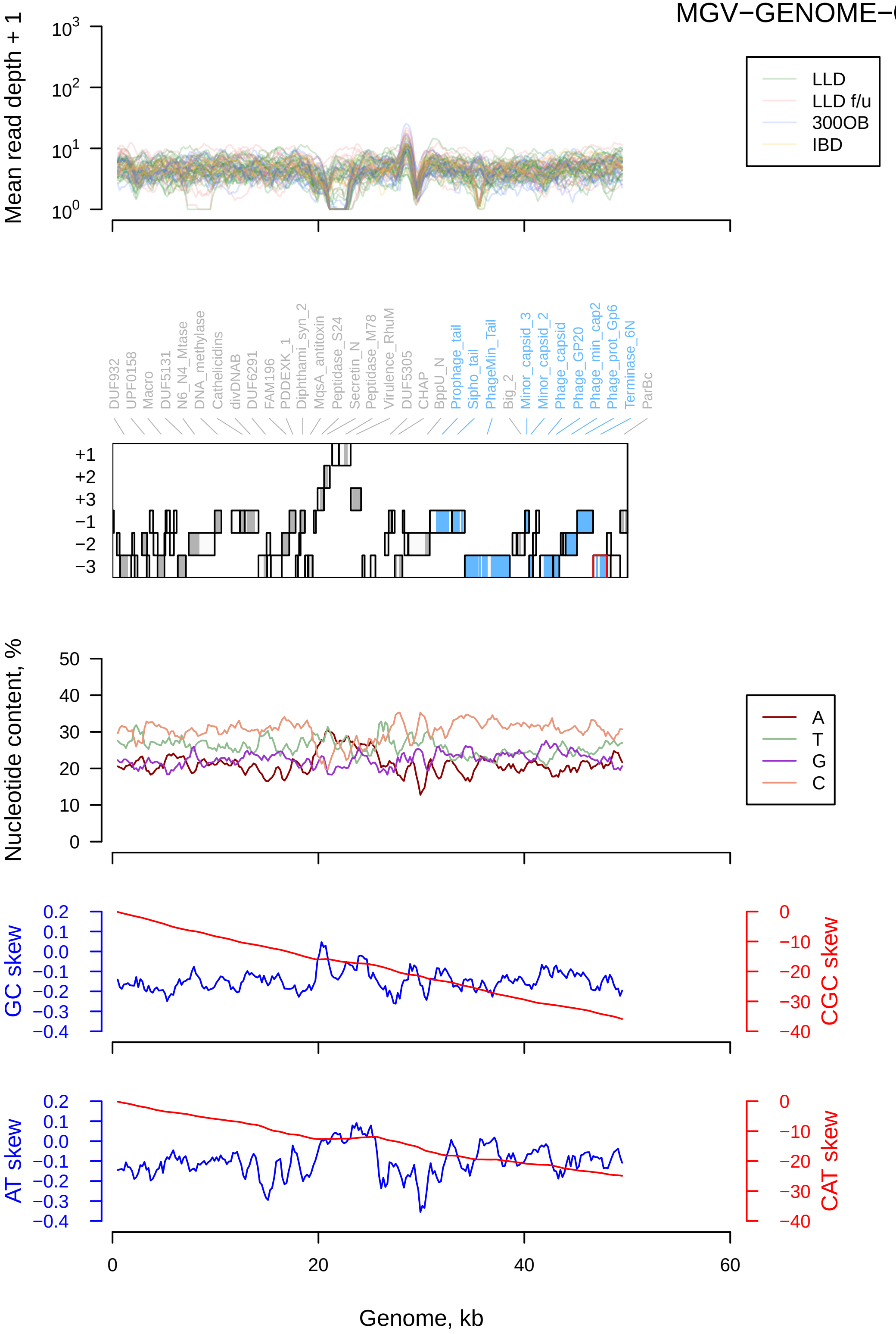

MGV−GENOME−0321287 (DTR, code 11)

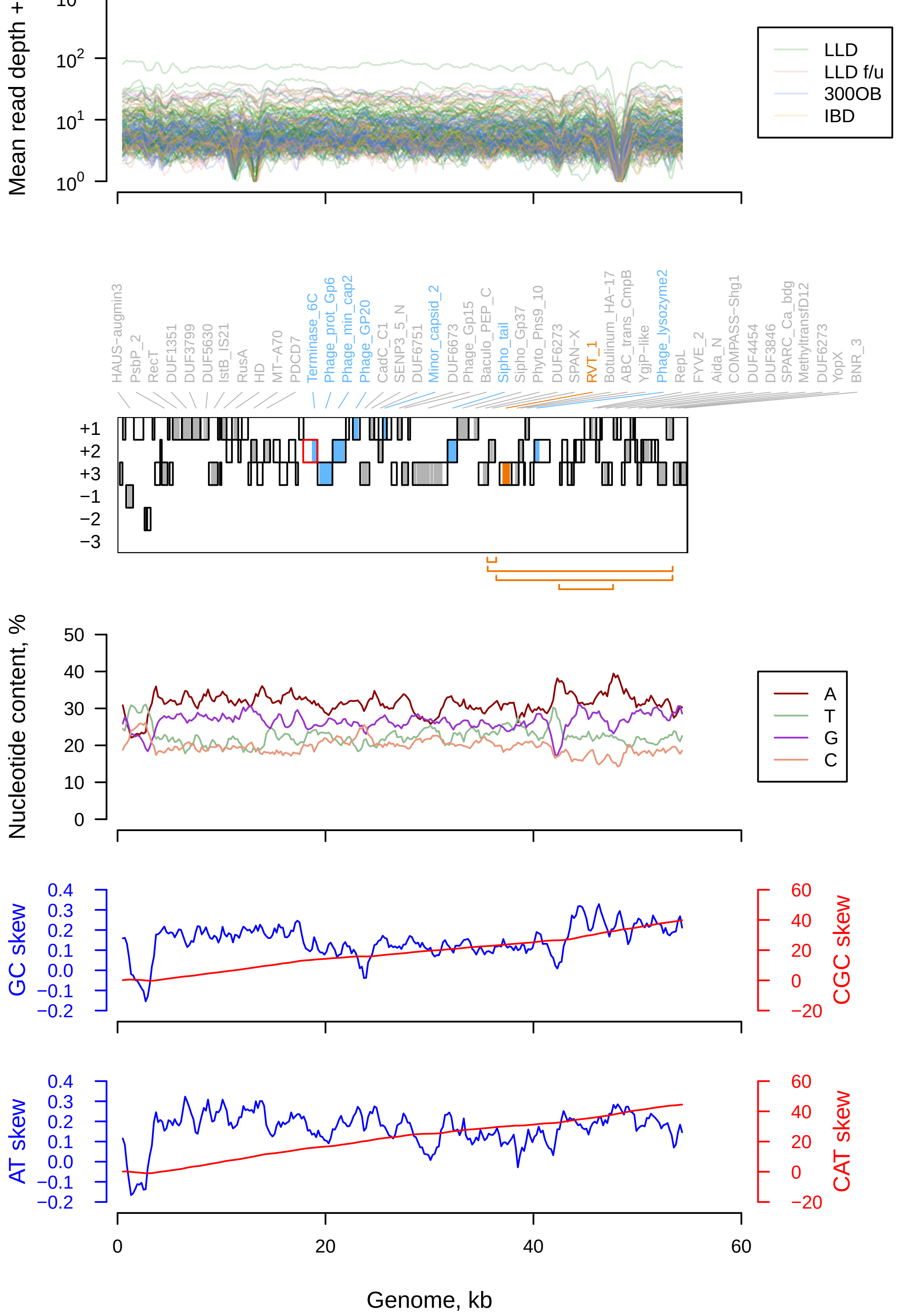

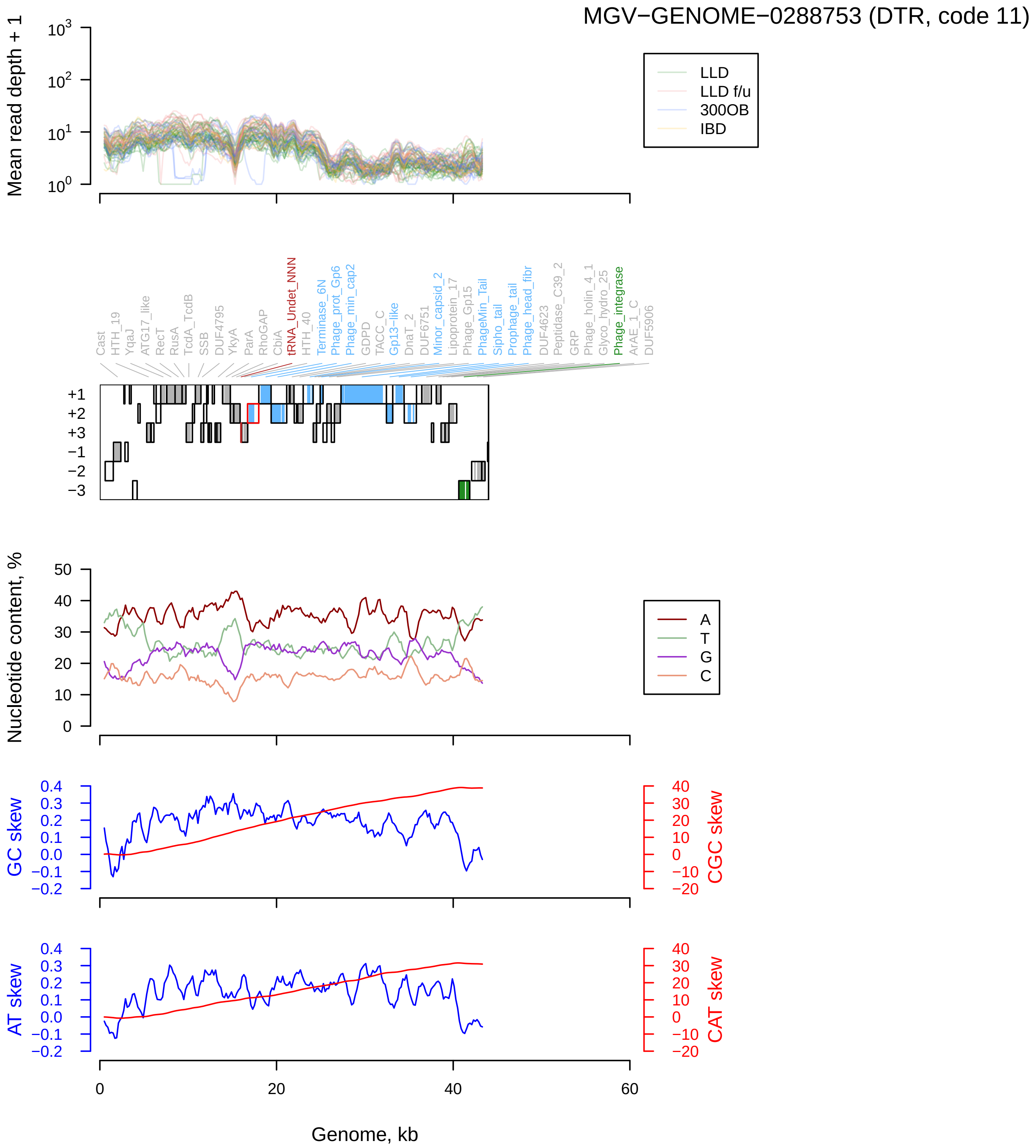

MGV−GENOME−0288753 (DTR, code 11)
Mean read depth + 1
LLD
LLD f/u
300OB
IBD
Cast
HTH_19
YqaJ
ATG17_like
RecT
RusA
TcdA_TcdB
SSB
DUF4795
YkyA
ParA
RhoGAP
CbiA
tRNA_Undet_NNN
HTH_40
Terminase_6N
Phage_prot_Gp6
Phage_min_cap2
GDPD
TACC_C
Gp13−like
DnaT_2
DUF6751
Minor_capsid_2
Lipoprotein_17
Phage_Gp15
PhageMin_Tail
Sipho_tail
Prophage_tail
Phage_head_fibr
DUF4623
Peptidase_C39_2
GRP
Phage_holin_4_1
Glyco_hydro_25
Phage_integrase
ArAE_1_C
DUF5906
Nucleotide content, %
A
T
G
C
GC skew
CGC skew
AT skew
CAT skew
Genome, kb

MGV−GENOME−0289925 (DTR, code 11)

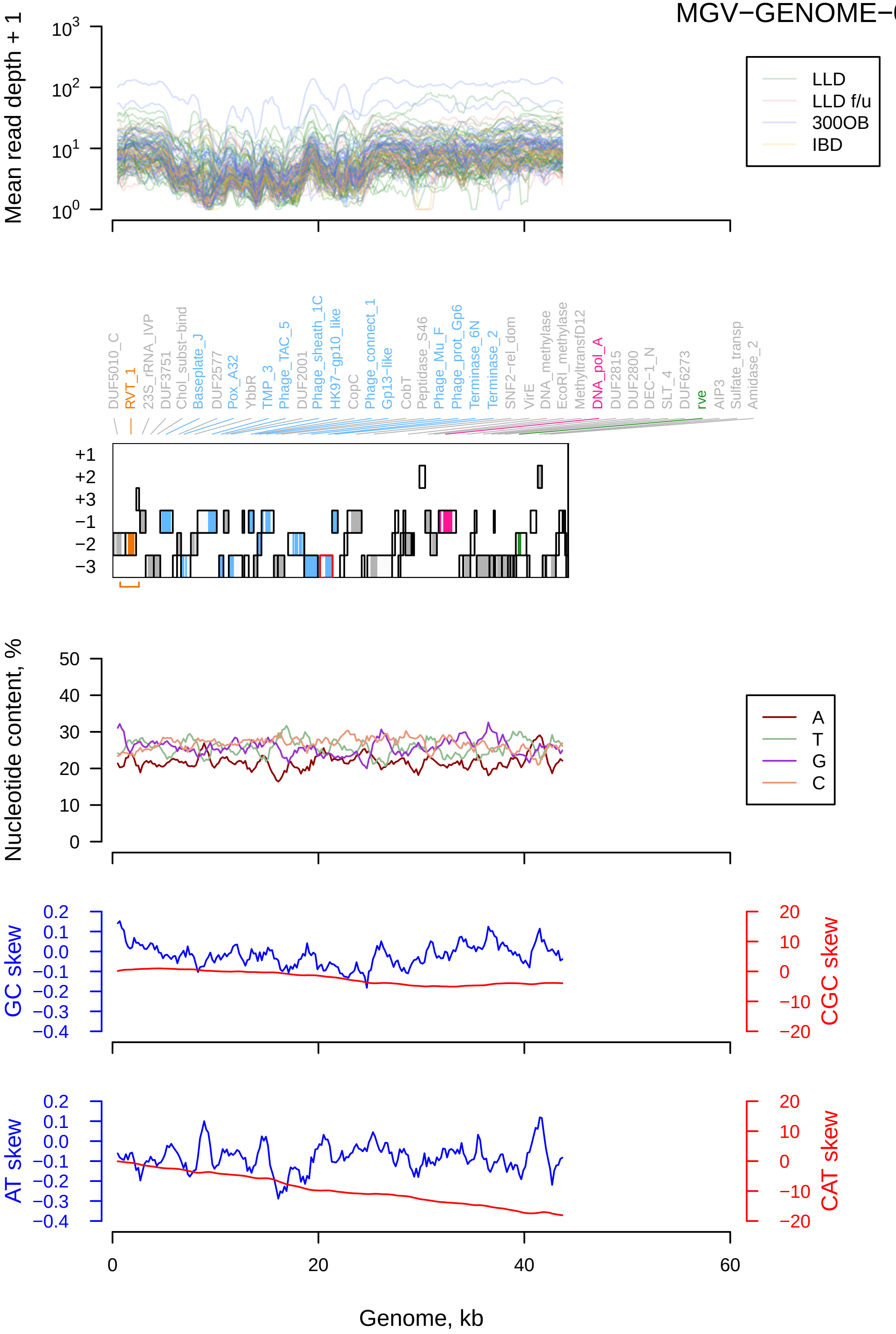

NL_vir036031 (DTR, code 11)

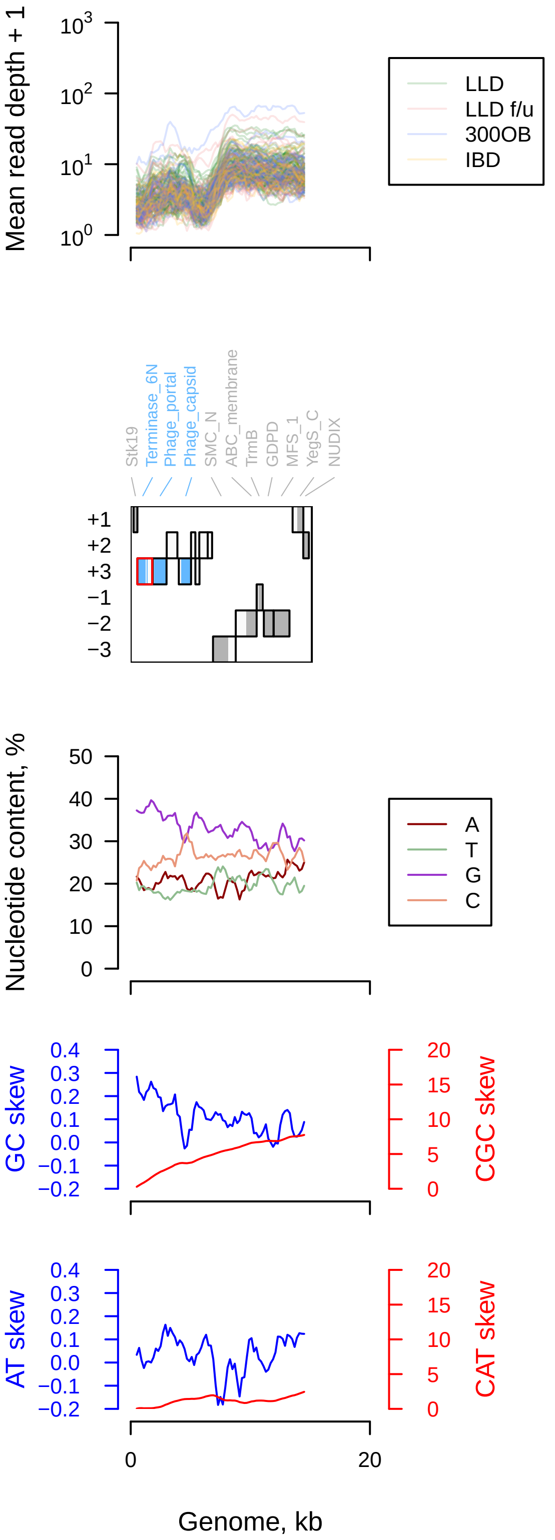

NL_vir053139 (DTR, code 11)

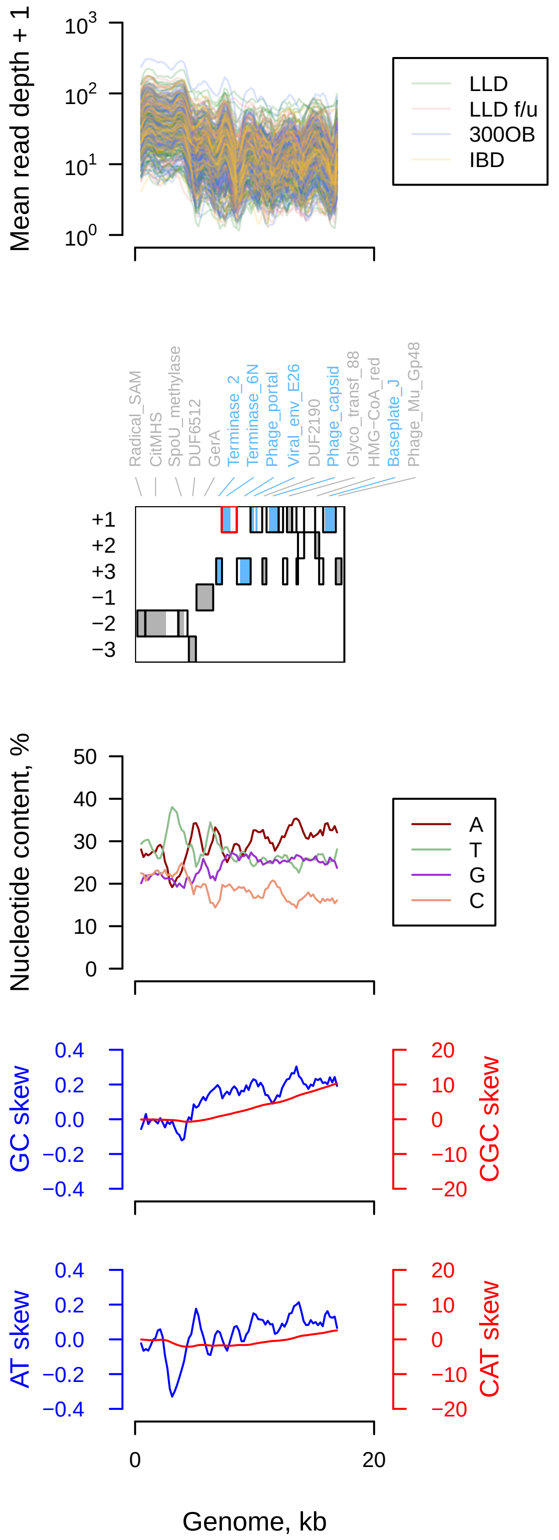

NL_vir018760 (DTR, code 11)

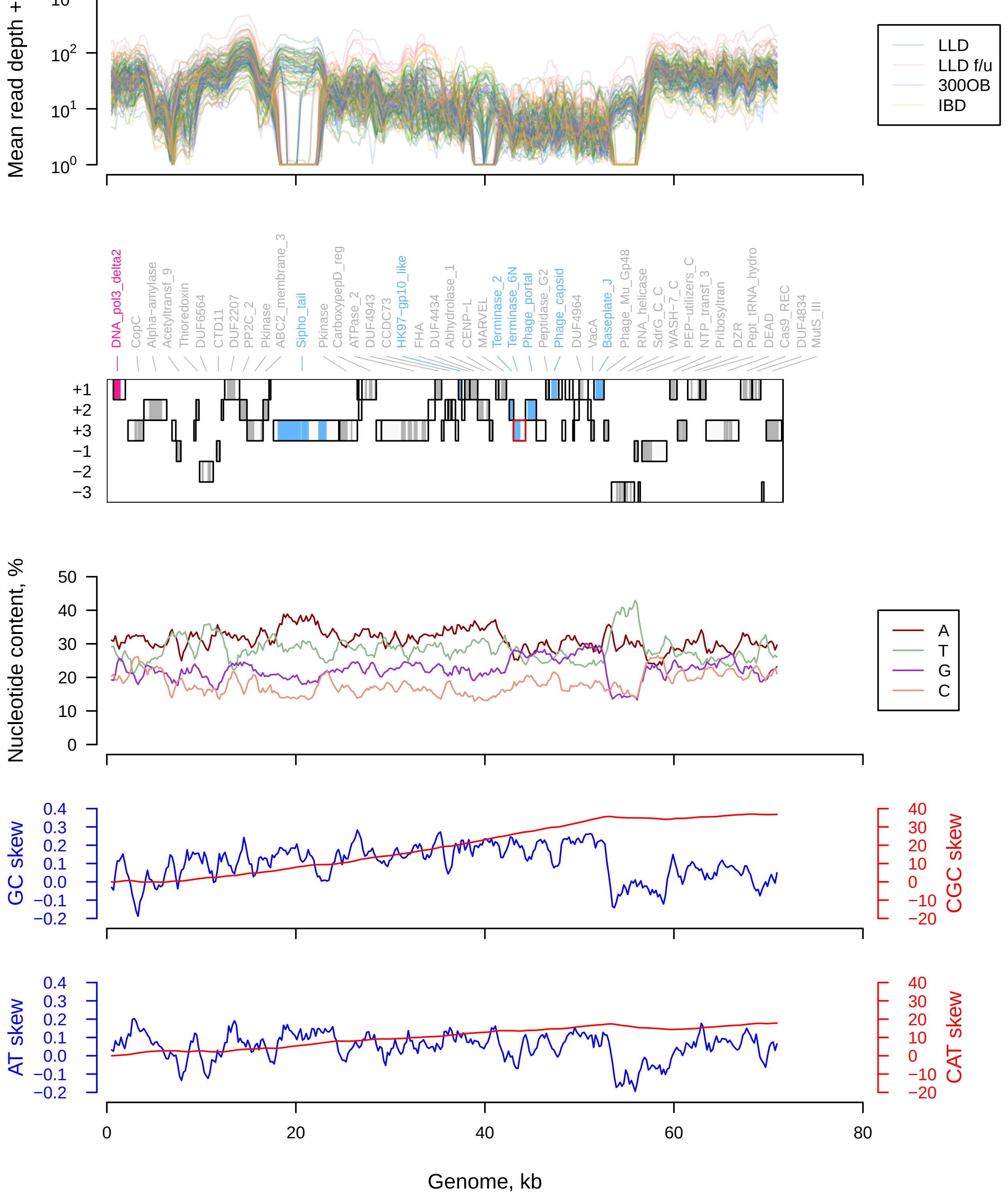

NL_vir026707 (DTR, code 11)

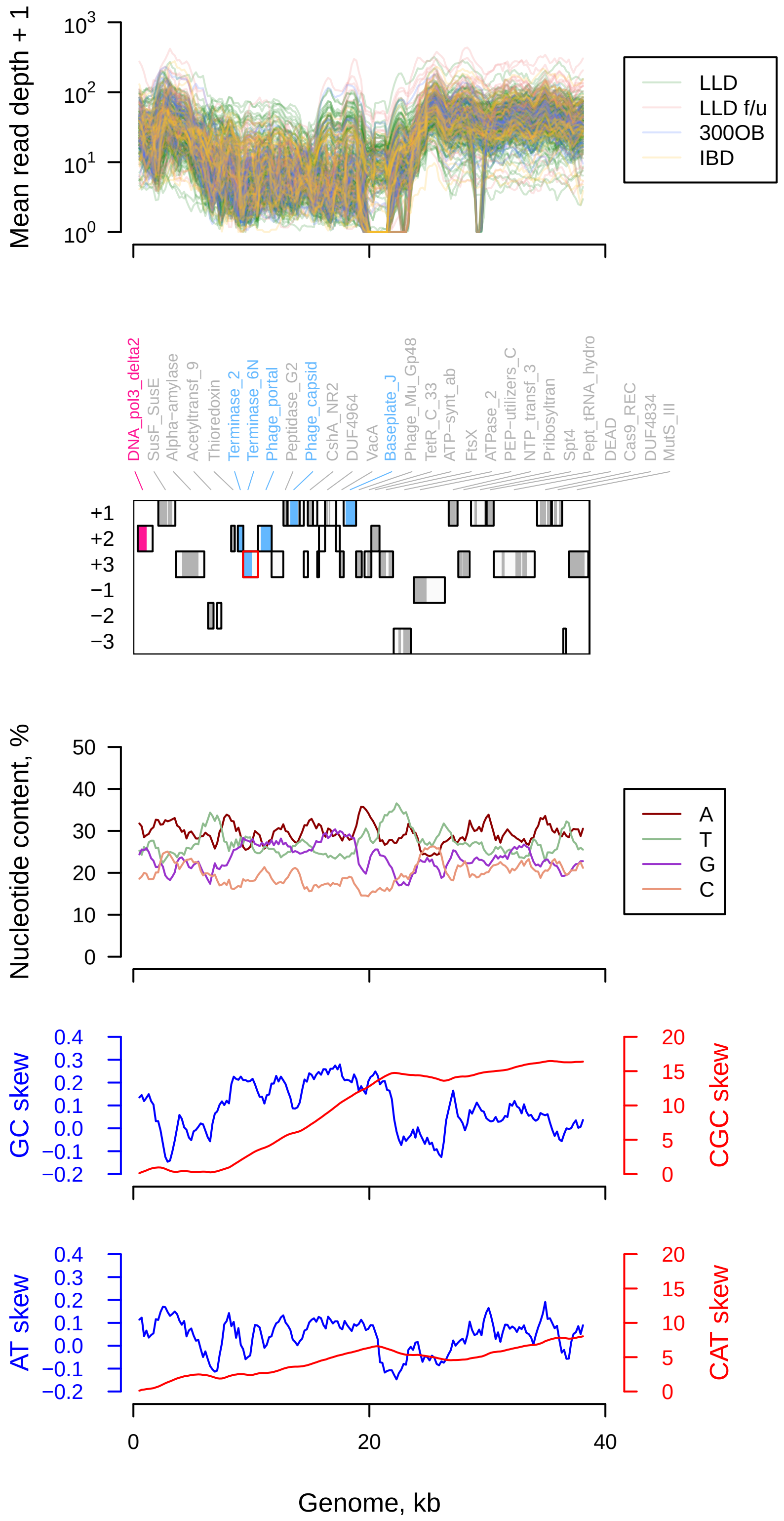

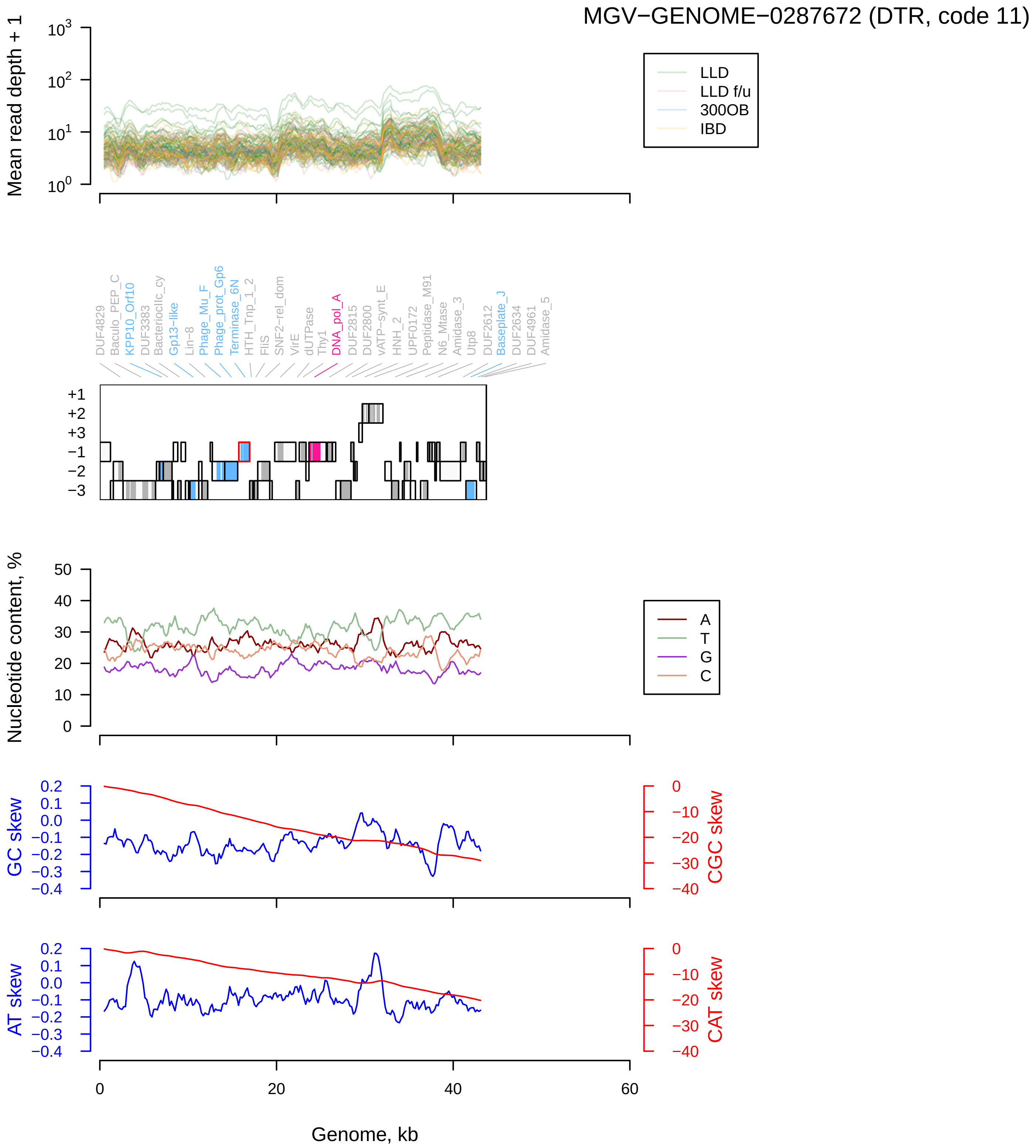
MGV−GENOME−0287672 (DTR, code 11)
Mean read depth + 1
LLD
LLD f/u
300OB
IBD
DUF4829
Baculo_PEP_C
KPP10_Orf10
DUF3383
Bacteriocin_cy
Gp13−like
Lin−8
Phage_Mu_F
Phage_prot_Gp6
Terminase_6N
HTH_Tnp_1_2
FliS
SNF2−rel_dom
VirE
dUTPase
Thy1
DNA_pol_A
DUF2815
DUF2800
vATP−synt_E
HNH_2
UPF0172
Peptidase_M91
N6_Mtase
Amidase_3
Utp8
DUF2612
Baseplate_J
DUF2634
DUF4961
Amidase_5
Nucleotide content, %
A
T
G
C
GC skew
CGC skew
AT skew
CAT skew
Genome, kb

NL_vir014968 (DTR, code 11)

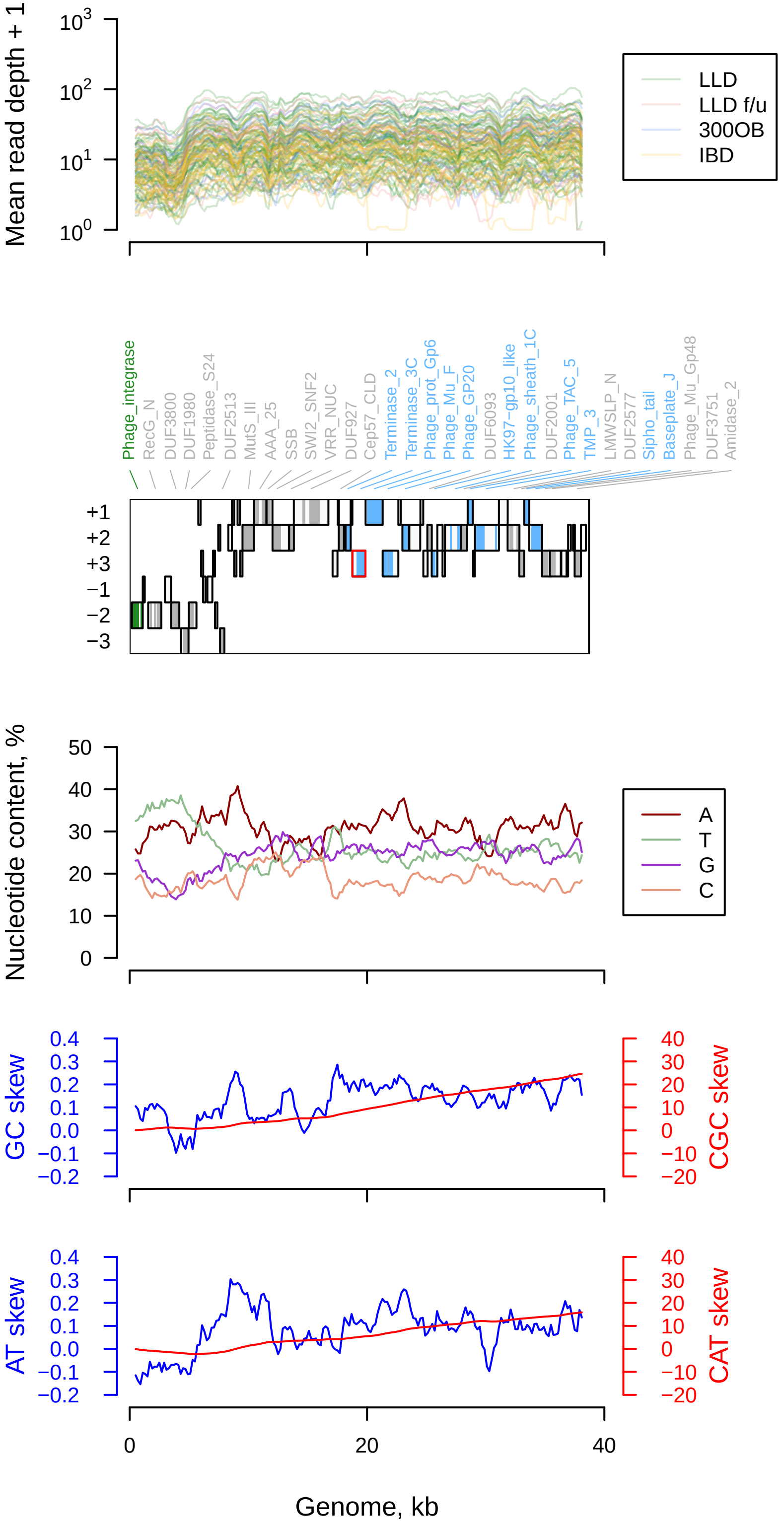

uvig_512347 (DTR, code 11)

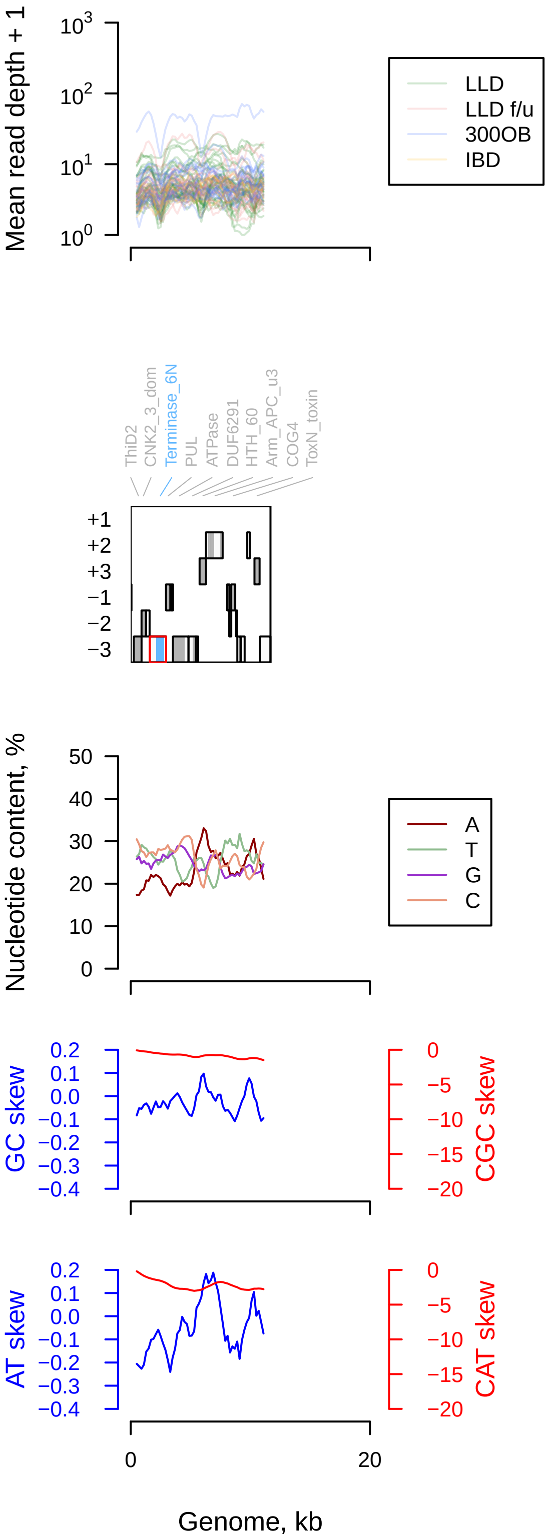

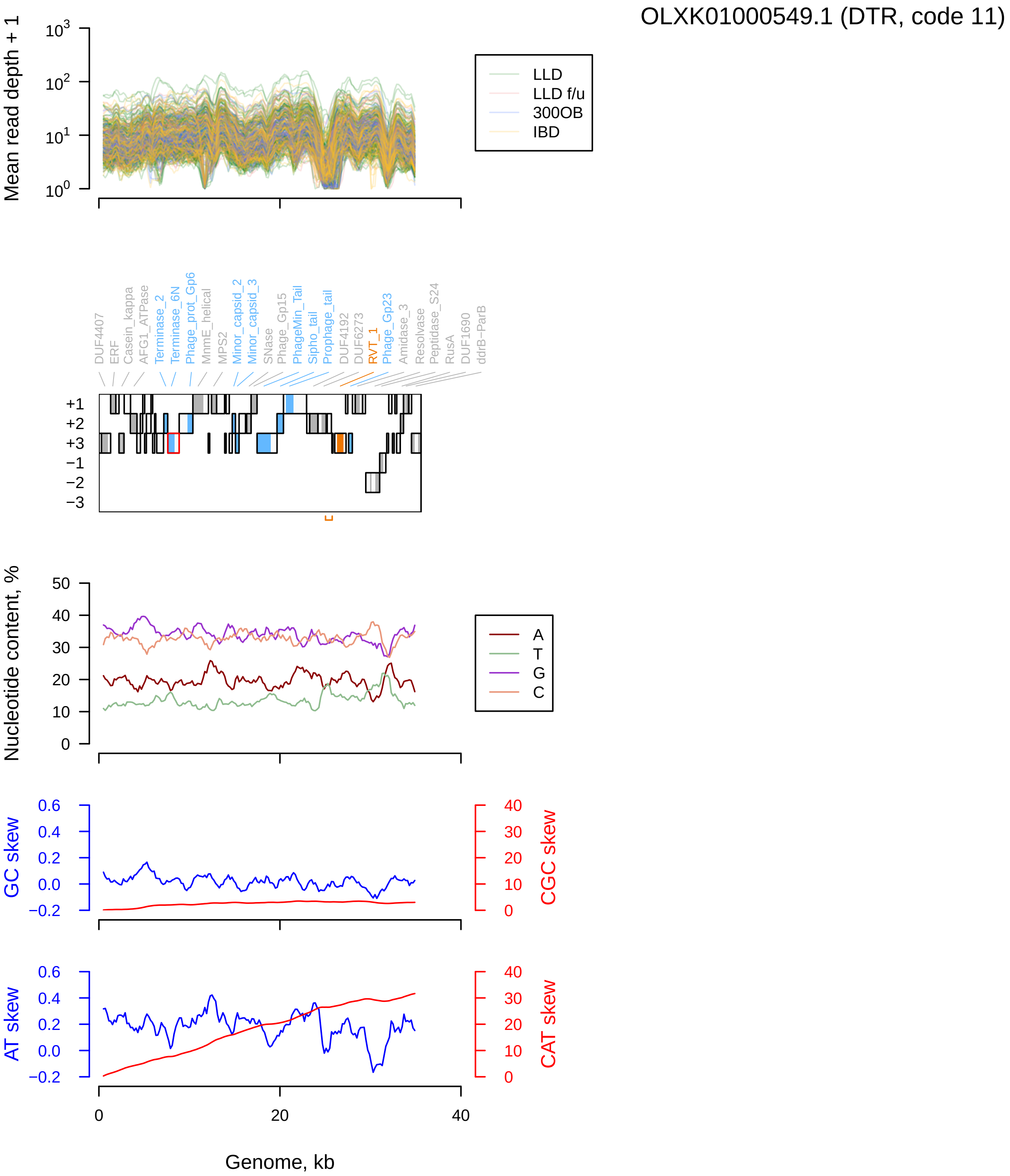

OLXK01000549.1 (DTR, code 11)
Mean read depth + 1
LLD
LLD f/u
300OB
IBD
Nucleotide content, %
A
T
G
C
GC skew
CGC skew
AT skew
CAT skew
Genome, kb

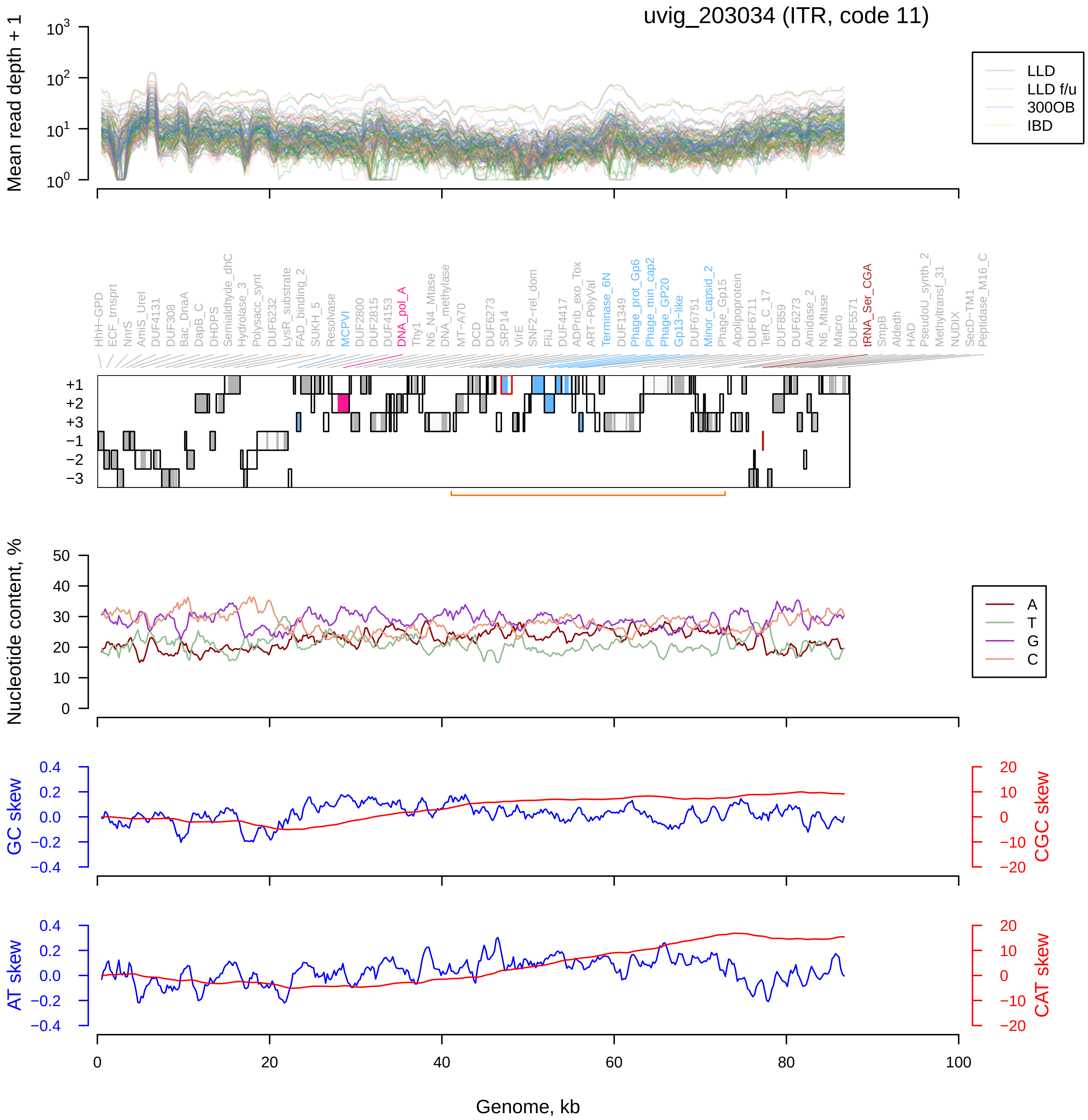

uvig_203034 (ITR, code 11)
Mean read depth + 1
LLD
LLD f/u
300OB
IBD
HhH−GPD
ECF_trnsprt
NnrS
AmiS_UreI
DUF4131
DUF308
Bac_DnaA
DapB_C
DHDPS
Semialdhyde_dhC
Hydrolase_3
Polysacc_synt
DUF6232
LysR_substrate
FAD_binding_2
SUKH_5
Resolvase
MCPVI
DUF2800
DUF2815
DUF4153
DNA_pol_A
Thy1
N6_N4_Mtase
DNA_methylase
MT−A70
DCD
DUF6273
SRP14
VirE
SNF2−rel_dom
FliJ
DUF4417
ADPrib_exo_Tox
ART−PolyVal
Terminase_6N
DUF1349
Phage_prot_Gp6
Phage_min_cap2
Phage_GP20
Gp13−like
DUF6751
Minor_capsid_2
Phage_Gp15
Apolipoprotein
DUF6711
TetR_C_17
DUF859
DUF6273
Amidase_2
N6_Mtase
Macro
DUF5571
tRNA_Ser_CGA
SmpB
Aldedh
HAD
PseudoU_synth_2
Methyltransf_31
NUDIX
SecD−TM1
Peptidase_M16_C
+1
+2
+3
−1
−2
−3
Nucleotide content, %
A
T
G
C
GC skew
CGC skew
AT skew
CAT skew
Genome, kb

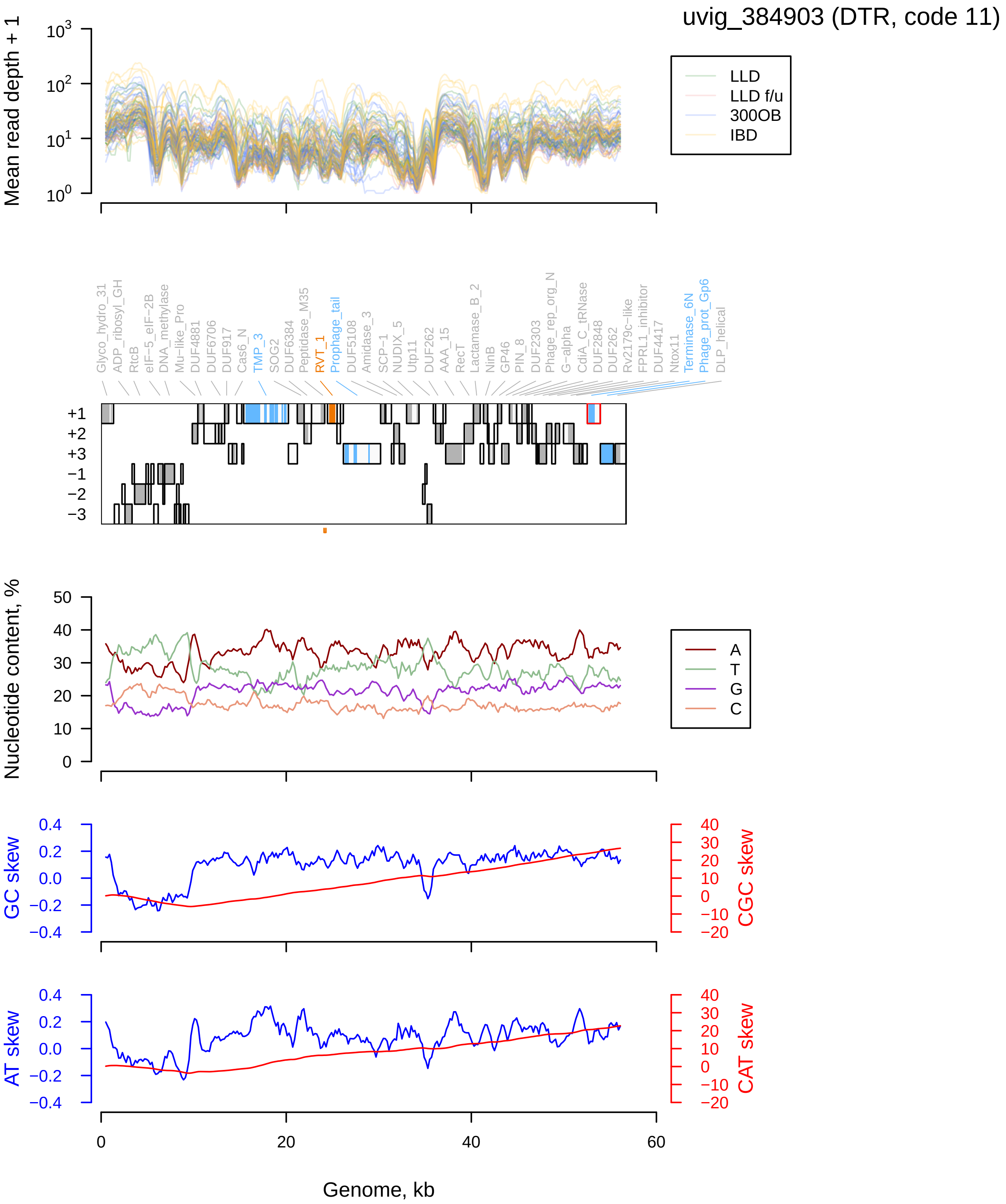
uvig_384903 (DTR, code 11)
Mean read depth + 1
LLD
LLD f/u
300OB
IBD
Glyco_hydro_31
ADP_ribosyl_GH
RtcB
eIF−5_eIF−2B
DNA_methylase
Mu−like_Pro
DUF4881
DUF6706
DUF917
Cas6_N
TMP_3
SOG2
DUF6384
Peptidase_M35
RVT_1
Prophage_tail
DUF5108
Amidase_3
SCP−1
NUDIX_5
Utp11
DUF262
AAA_15
RecT
Lactamase_B_2
NinB
GP46
PIN_8
DUF2303
Phage_rep_org_N
G−alpha
CdiA_C_tRNase
DUF2848
DUF262
Rv2179c−like
FPRL1_inhibitor
DUF4417
Ntox11
Terminase_6N
Phage_prot_Gp6
DLP_helical
Nucleotide content, %
A
T
G
C
GC skew
CGC skew
AT skew
CAT skew
Genome, kb

ERS698742_NODE_4_length_57489_cov_840.739161 (DTR, code 11)

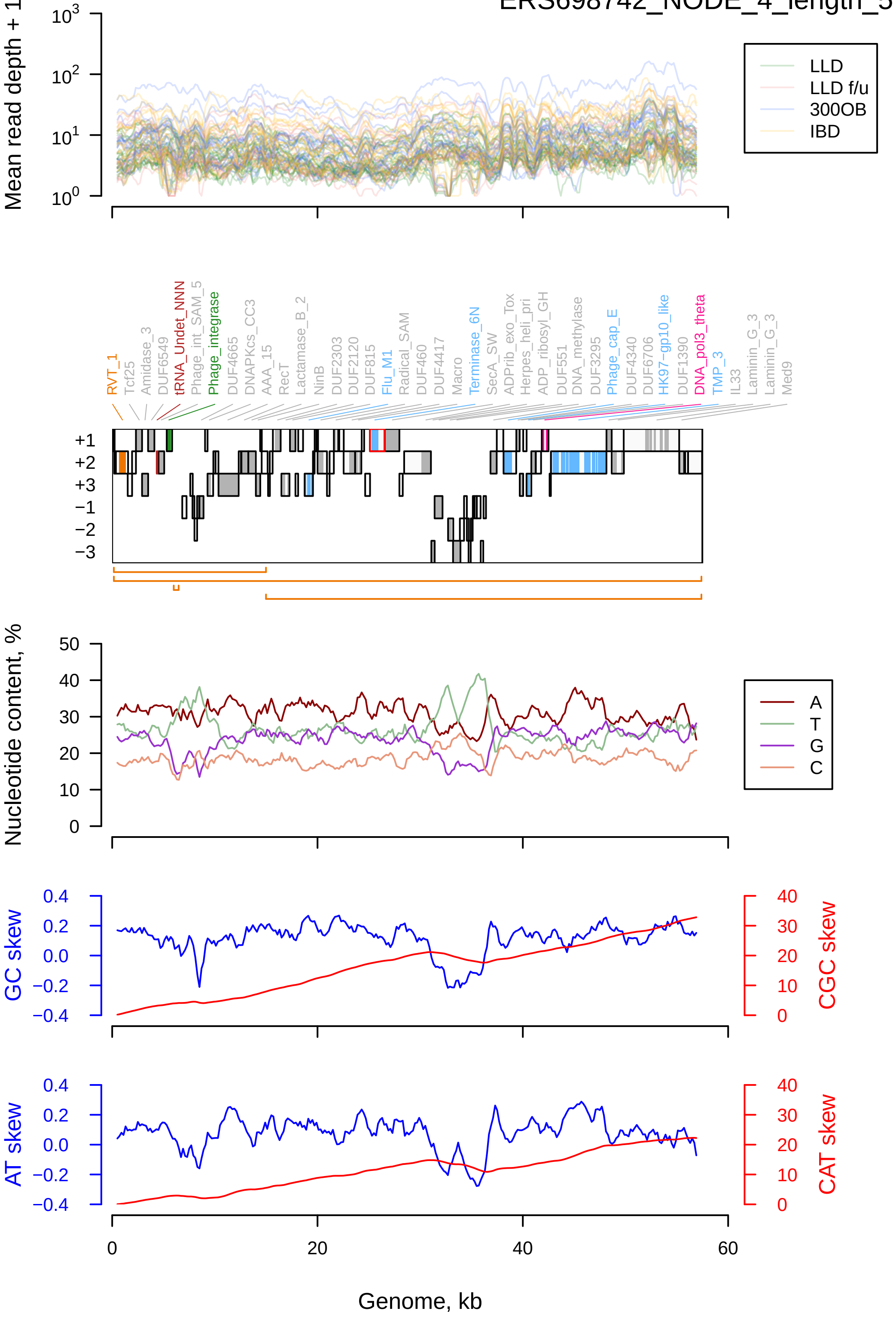

MGV−GENOME−0279285 (DTR, code 11)

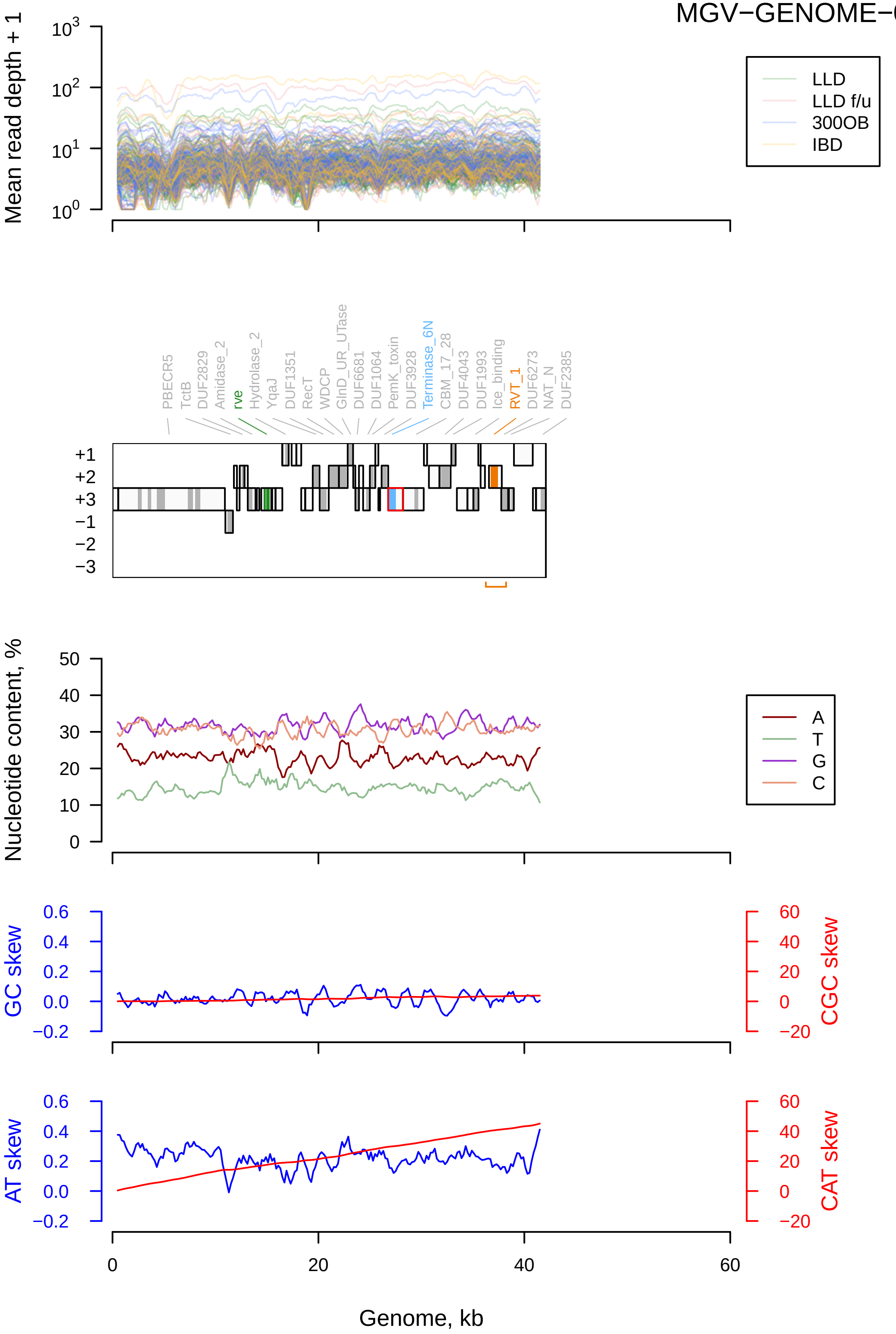

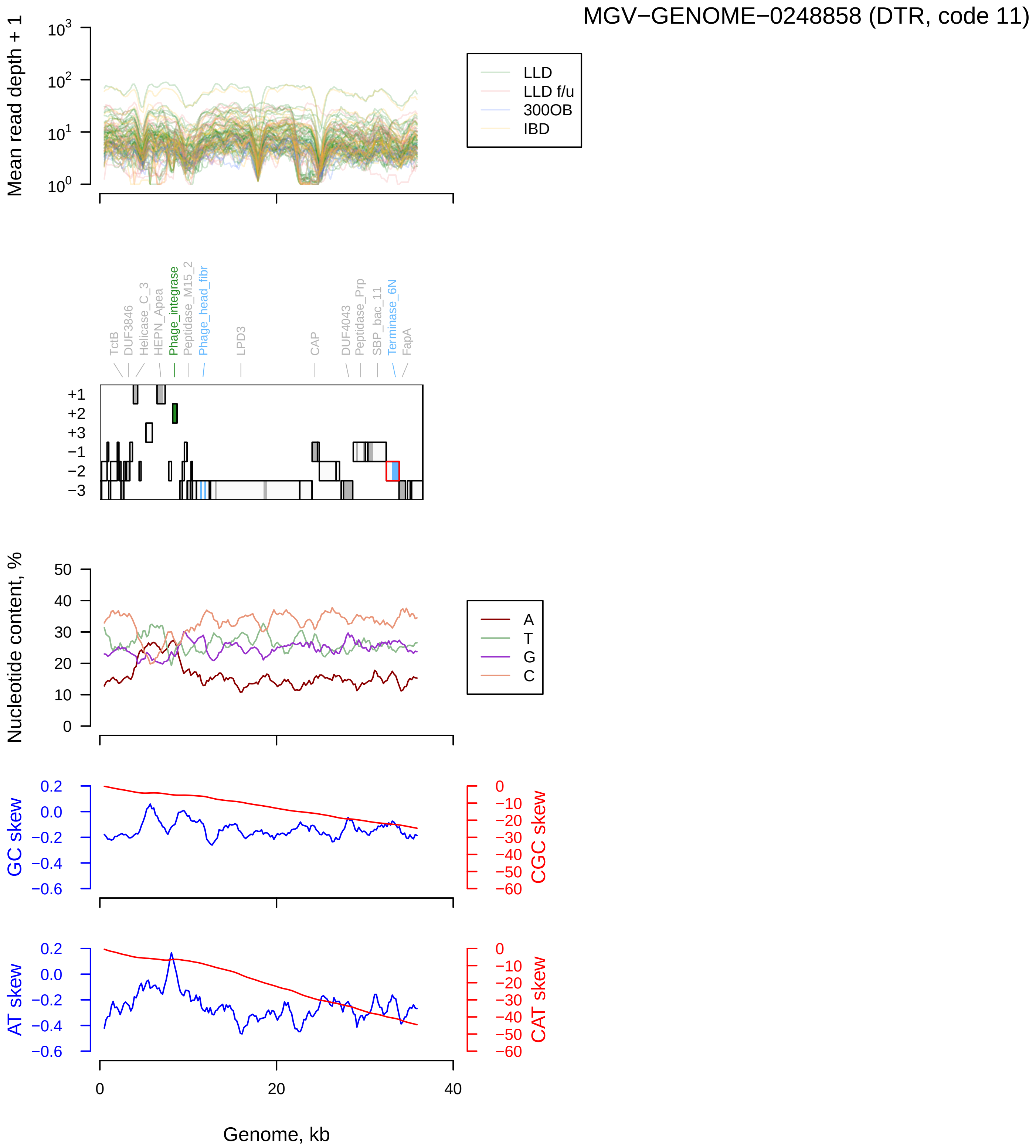
MGV−GENOME−0248858 (DTR, code 11)
Mean read depth + 1
LLD
LLD f/u
300OB
IBD
TctB
DUF3846
Helicase_C_3
HEPN_Apea
Phage_integrase
Peptidase_M15_2
Phage_head_fibr
LPD3
CAP
DUF4043
Peptidase_Prp
SBP_bac_11
Terminase_6N
FapA
Nucleotide content, %
A
T
G
C
GC skew
CGC skew
AT skew
CAT skew
Genome, kb

NL_vir049144 (DTR, code 11)

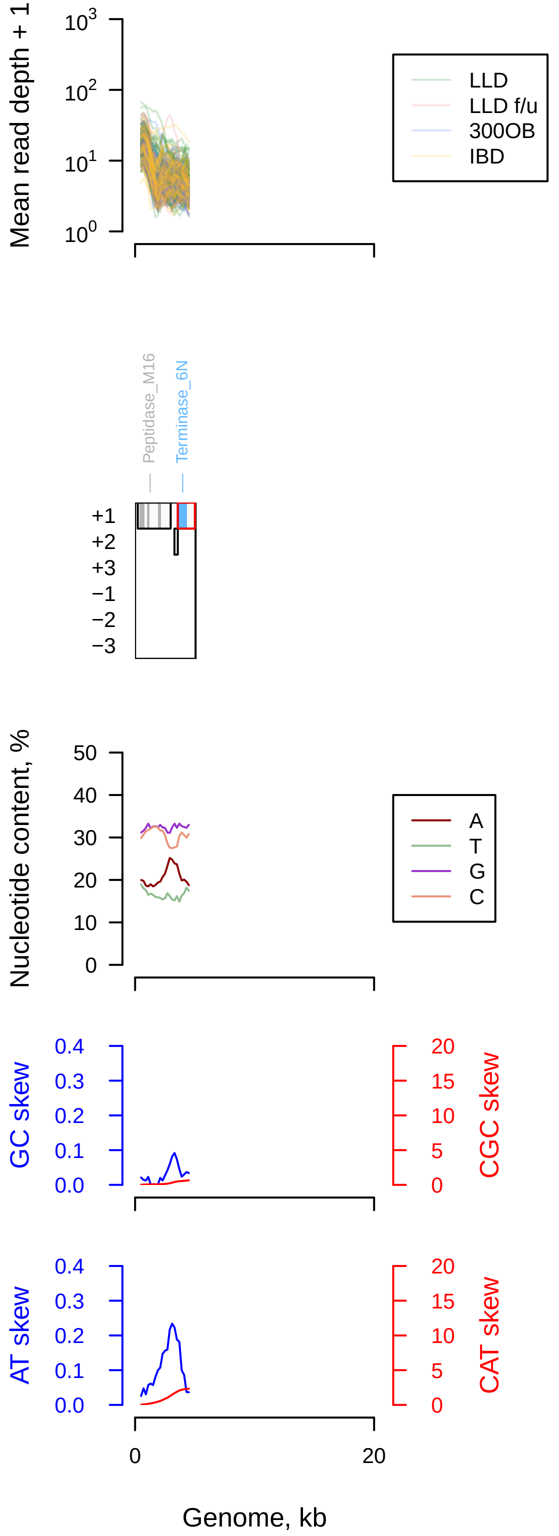

MGV−GENOME−0328053 (DTR, code 11)

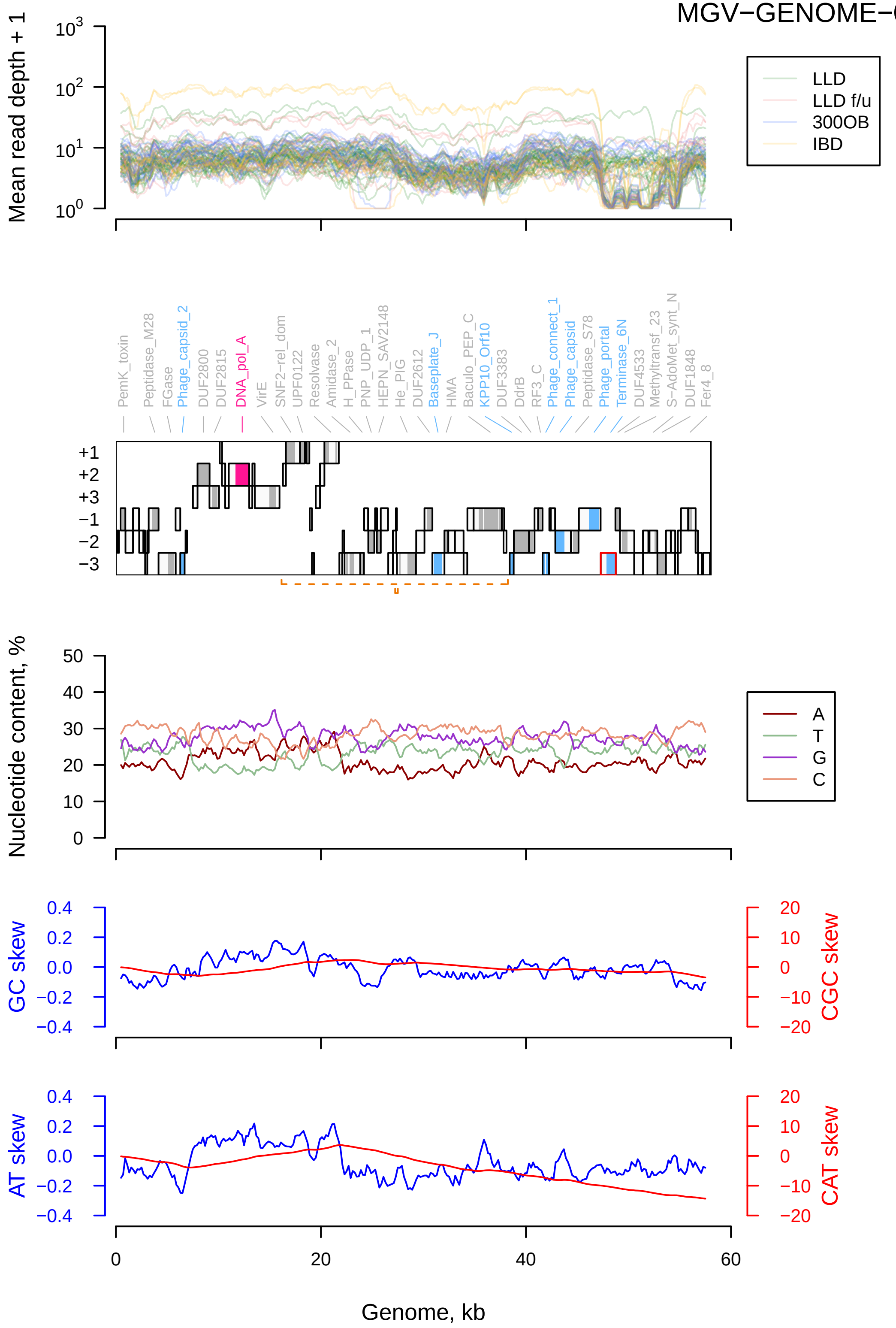

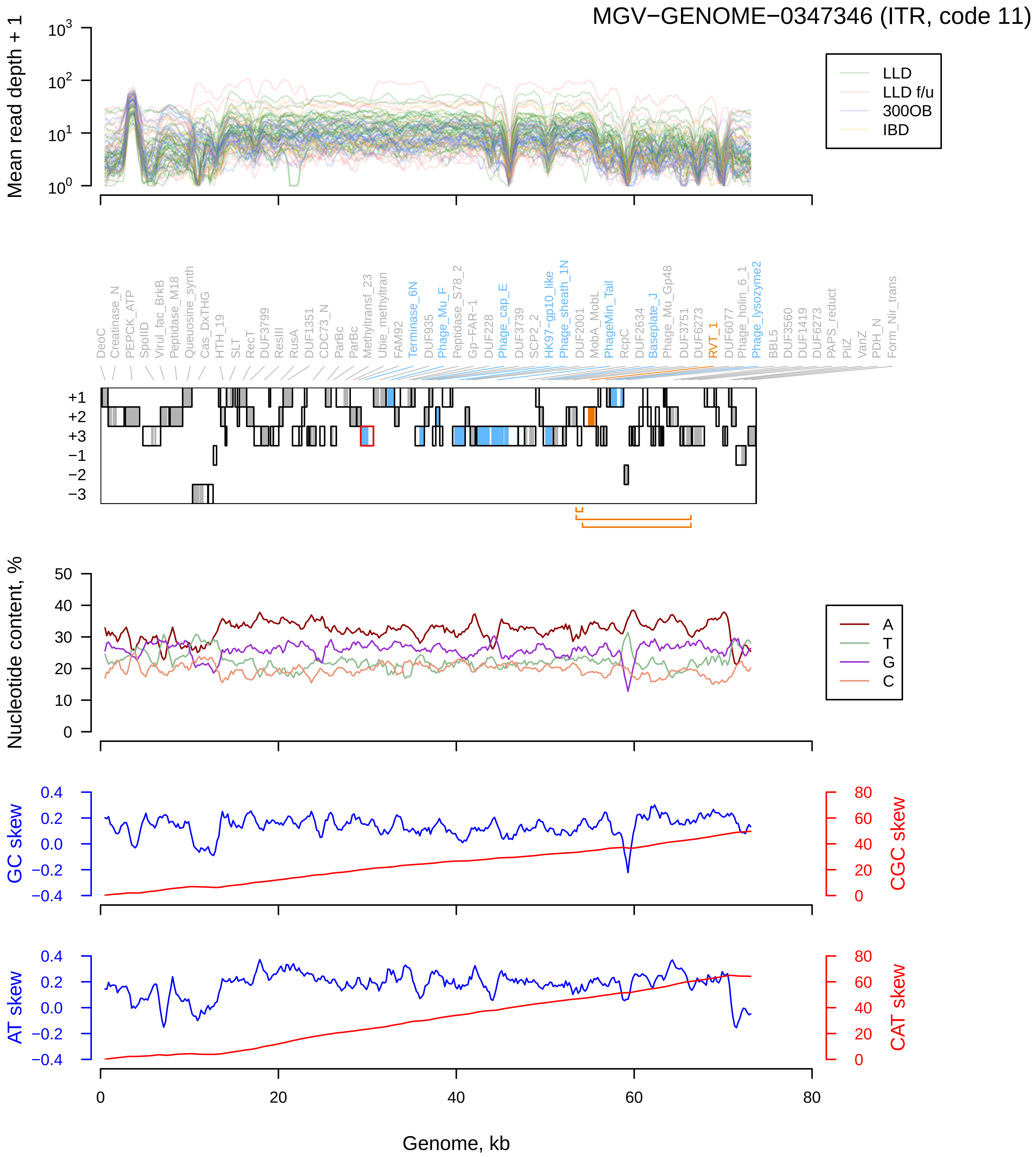

MGV−GENOME−0347346 (ITR, code 11)
Mean read depth + 1
LLD
LLD f/u
300OB
IBD
DeoC
Creatinase_N
PEPCK_ATP
SpoIID
Virul_fac_BrkB
Peptidase_M18
Queuosine_synth
Cas_DxTHG
HTH_19
SLT
RecT
DUF3799
ResIII
RusA
DUF1351
CDC73_N
ParBc
ParBc
Methyltransf_23
Ubie_methyltran
FAM92
Terminase_6N
DUF935
Phage_Mu_F
Peptidase_S78_2
Gp-FAR-1
DUF228
Phage_cap_E
DUF3739
SCP2_2
HK97-gp10_like
Phage_sheath_1N
DUF2001
MobA_MobL
PhageMin_Tail
RcpC
DUF2634
Baseplate_J
Phage_Mu_Gp48
DUF3751
DUF6273
RVT_1
DUF6077
Phage_holin_6_1
Phage_lysozyme2
BBL5
DUF3560
DUF1419
DUF6273
PAPS_reduct
PilZ
VanZ
PDH_N
Form_Nir_trans
Nucleotide content, %
A
T
G
C
GC skew
CGC skew
AT skew
CAT skew
Genome, kb
