# Supplementary material for: Diversity and Ecology of Caudoviricetes Phages with Genome Terminal Repeats in Fecal Metagenomes from Four Dutch Cohorts
Source: Viruses. 2022 Oct 20;14(10):2305. doi: 10.3390/v14102305 (PMC9610469; doi:10.3390/v14102305)
Supplement: Supplementary file 1 [file viruses-14-02305-s001.zip › Supplementary_legends.pdf]

Figure S1. Workflow leading to delineation of the CGTR1899 and CGTR54 databases.

Figure S2. TerL alignment conservation. Mean conservation of the multiple sequence alignment of TerL proteins encoded by the CGTR1899 vOTU representatives (Table S2) was calculated using an 11-column window sliding along the MSA with a 1-column step. MSA columns with ≥ 50% gaps were excluded from consideration. Adenosine triphosphatase motifs Walker A and B and nuclease motifs I, II and III are each highlighted by a distinct color. The sequence logo of each motif is presented below the conservation profile, and the color of the frame designates the motif.

Figure S3. Gene-sharing network of viruses with evolutionary related terminase genes. Genomes representing the CGTR1899 vOTUs, complete genomes of the class *Caudoviricetes* viruses from Viral RefSeq 209, complete genomes of the class *Herviviricetes* viruses from Viral RefSeq 209 and genomes of the recently discovered phylum *Mirusviricota* viruses are represented by green, orange, blue and red dots, respectively. Connections between genomes are indicative of shared gene content.

Figure S4. Sequence similarity between genomes representing the CGTR54 vOTUs and extensively characterized phages. Each dot plot illustrates similarity between a pair of sequences, X-axis corresponds to a sequence from the literature, Y-axis corresponds to a CGTR54 sequence. Coordinates are indicated in kilobases. Every 12-letter word shared by a pair of sequences is presented as a black dot on a dot plot. If a reverse complement of a sequence was analyzed, the letters "RC" are added to the sequence identifier. Contig length and coverage are omitted from the sequence identifiers for brevity, where applicable. The color of the dot plot frame points to a publication about the X-axis phage: blue, *Minot et al. 2012*; green, *Ly et al. 2016*; pink, *Dzunkova et al. 2019*. See Table S4.

Table S1. Properties of the CGTR1899 phage genome sequences. vOTUs are ordered according to their position on the TerL-based phylogenetic tree (Figure 3). Properties of the phage genome sequences identified in the form of prophage contigs were assessed after the fragments of microbial genomes were cleaved off.

Table S2. Properties of the CGTR1899 vOTU representatives. TerL gene coordinates are specified by indicating the genome strand (f, forward or r, reverse) and coordinates separated by semicolons. The number of positive samples is indicated for the four Dutch cohorts and for all and healthy adult (HA) Danish fecal viromes (DFV).

Table S3. Predicted phage hosts. First page, results of the prophage-based host prediction conducted for the CGTR1899 database. Second page, results of the CRISPR-based host prediction conducted for the representatives of the CGTR1899 vOTUs. Third page, associations between the relative abundances of the CGTR54 vOTUs and microbial taxa. Microbial taxa are designated using the MetaPhlAn software format, with "k__", "p__", "c__", "o__", "f__", "g__" and "s__" standing for kingdom, phylum, class, order, family, genus and species, respectively. Fourth page, comparison between host predictions made by the different methods. Data are shown for those CGTR54 vOTUs for which predictions by multiple methods were available. Prophage-, CRISPR- and co-abundance-based predictions

are shown with blue, orange and white backgrounds, respectively. Co-abundance-based predictions that diverge from other predictions at phylum level are highlighted by red font.

Table S4. Extensively characterized phage sequences similar to the CGTR54 vOTU representatives.

Table S5. Associations with human phenotypes. First page, associations between the 207 LLD cohort phenotypes and prevalence of the CGTR54 vOTUs. Second page, definition of the 207 LLD cohort phenotypes considered in the analysis. Subsequent pages, results of the analyses comparing the prevalence of the 54 vOTUs between the following groups: (1) LLD vs. 300OB cohort, (2) LLD vs. IBD cohort, (3) within 300OB cohort: absence vs. presence of metabolic syndrome, (4) within IBD cohort: CD vs. UC, and (5) within IBD cohort: exclusively colonic vs. ileal-inclusive disease location. Significant associations (FDR < 0.05) are highlighted by green background.

Material S1. Characteristics of the CGTR54 genomes. Each page corresponds to a genome representing a CGTR54 vOTU. Genome name, predicted genetic code and type of terminal repeats are indicated at the top of the page. *Top panel*, coverage by reads from the four Dutch cohorts: each line corresponds to a sample and represents mean coverage depth in a sliding window. The color of the line indicates cohort. *Second panel from top*, genome map. Genome is represented by a white bar in black frame. Three forward and three reverse frames are shown. ORFs are represented by light gray bars in black frames. Red frame denotes the TerL ORF key to recognizing the genome as belonging to the class *Caudoviricetes*. Regions of an ORF matching the PFAM profile used for its annotation are indicated by color: blue – structural protein profiles and profiles of proteins implicated in assembly of virus particles, pink – DNA polymerase profiles, green – integrase profiles, orange – reverse transcriptase profiles, and dark gray – all other profiles. Names of the profiles are indicated above the genome map (note that in some cases subdomains justifying the name of the profile may not be part of the alignment between the profile and the ORF product). Predicted tRNA genes are shown by dark red bars, and their names include the tRNA isotype and anticodon. Position of nucleotide repeats is indicated by vertical orange lines below the genome map, with pairs of repeats connected by horizontal orange lines. If one of the two repeats lies in the reverse strand, the orange line is dashed. *Third panel from top*, average content of the four nucleotides along the genome. *Bottom panels*, regular and cumulative GC- and AT-skew. All curves were generated using a 1,001 nt window sliding with a 200 nt step.

Material S2. MSA of RTs from the CGTR54 genomes representing vOTUs. Absolutely conserved residues are shown on red background and partially conserved residues in red font. Conserved motifs are underlined in green. Their names are indicated underneath. Name of each protein sequence consists of a genome name followed by strand (f, forward or r, reverse) and coordinates of the RT gene separated by semicolons. Contig length and coverage are omitted from the genome names for brevity, where applicable.

Text S1. Benchmarking of virus detection and taxonomic assignment.
