# Supplementary figures and images for: Diversity and Ecology of Caudoviricetes Phages with Genome Terminal Repeats in Fecal Metagenomes from Four Dutch Cohorts
Source: Viruses. 2022 Oct 20;14(10):2305. doi: 10.3390/v14102305 (PMC9610469; doi:10.3390/v14102305)

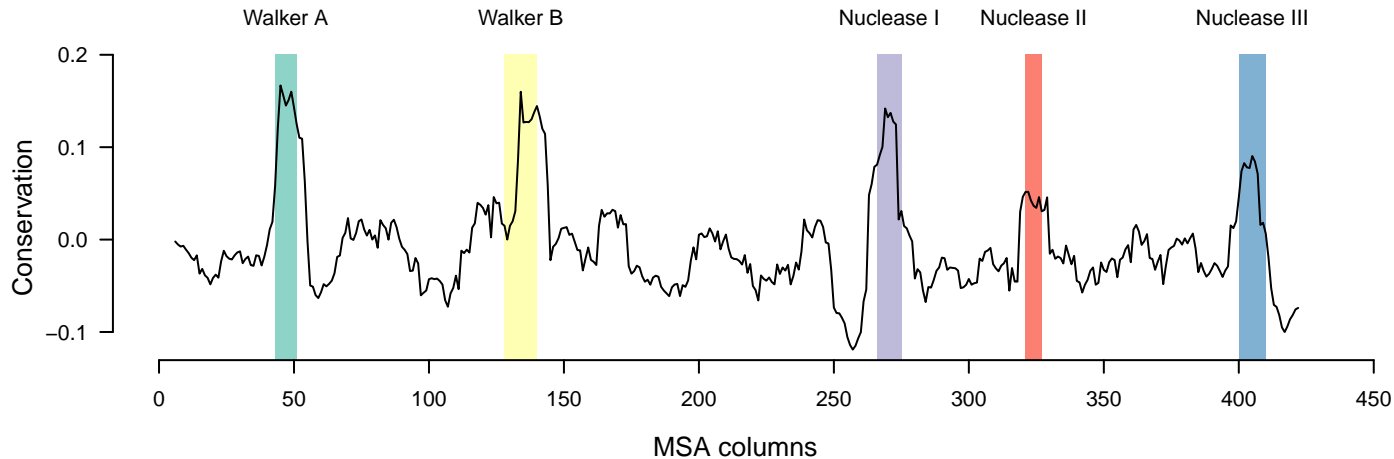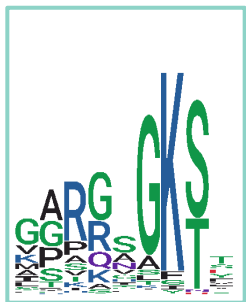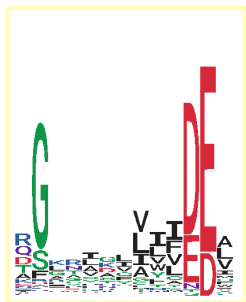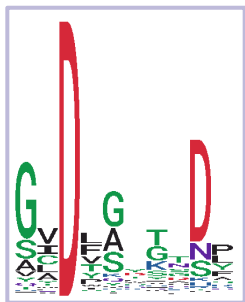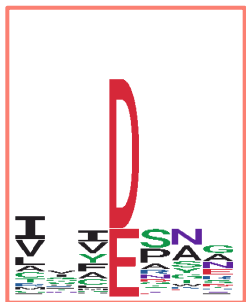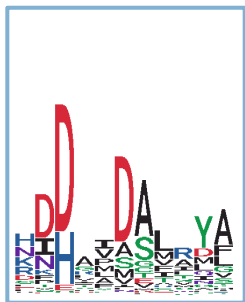

Supplement: Supplementary file 1 [file viruses-14-02305-s001.zip › Figure_S2.pdf]

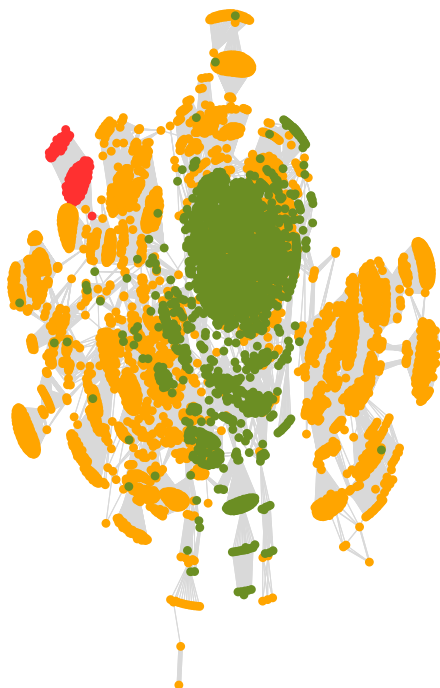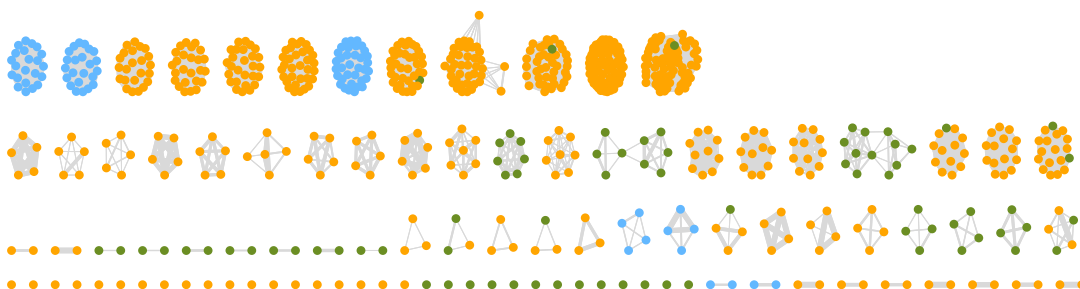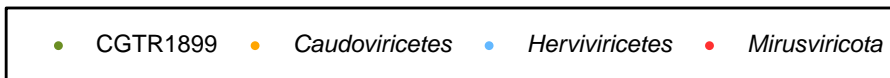

Supplement: Supplementary file 1 [file viruses-14-02305-s001.zip › Figure_S3.pdf]

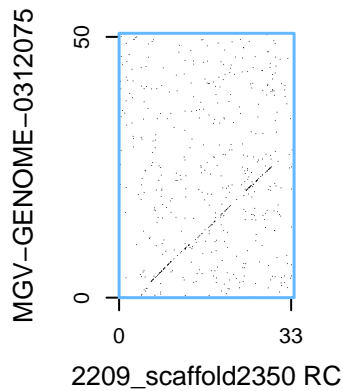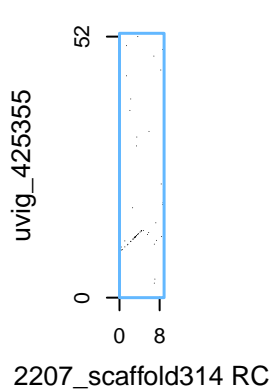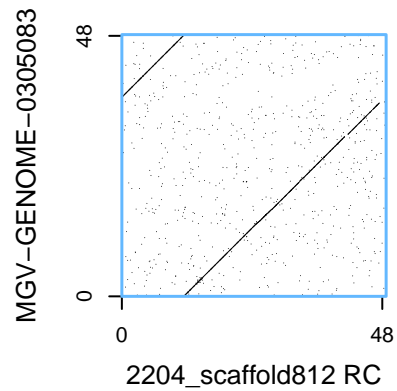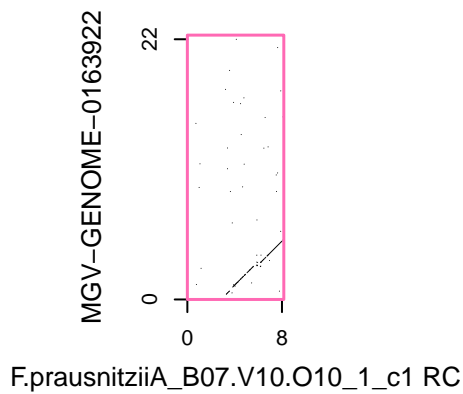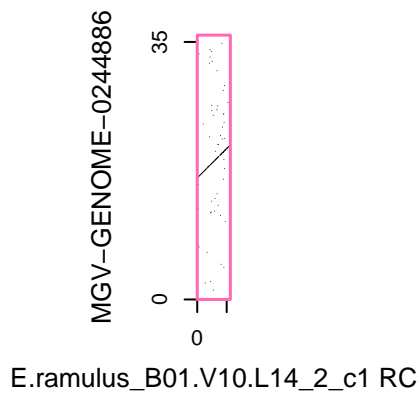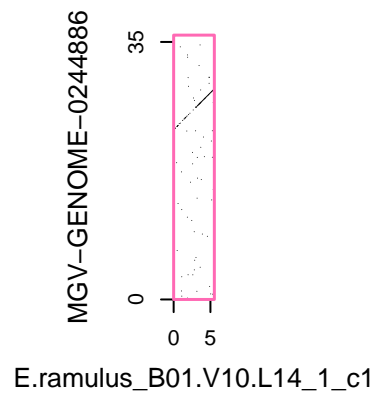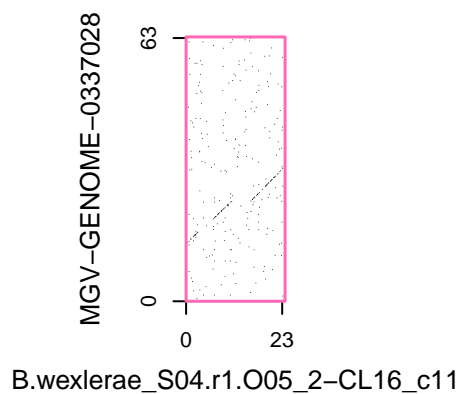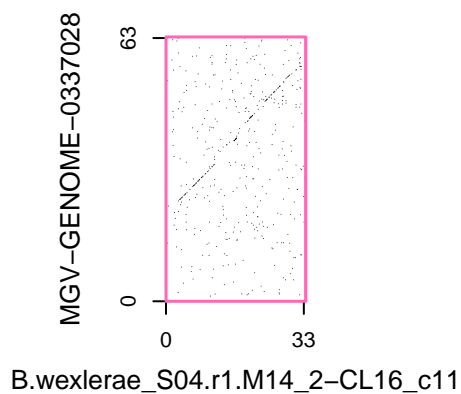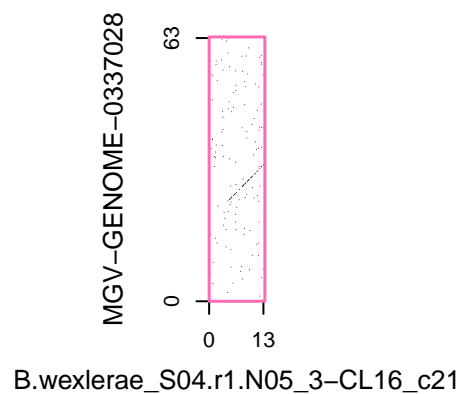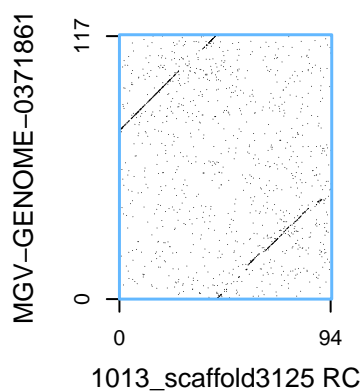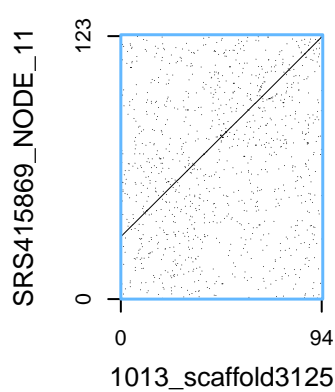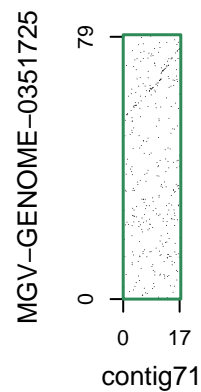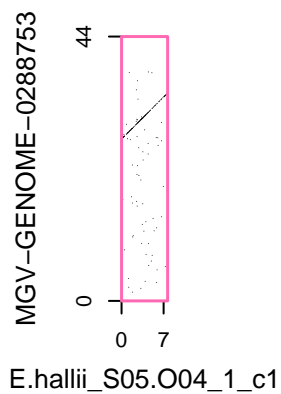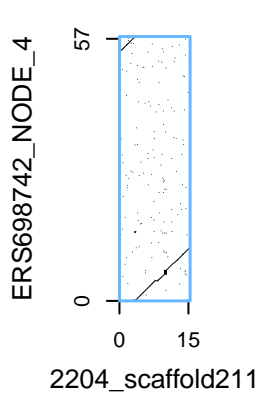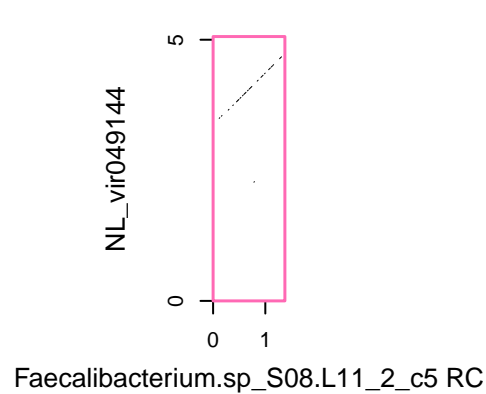

Supplement: Supplementary file 1 [file viruses-14-02305-s001.zip › Figure_S4.pdf]
